# A Calibrated Deep Learning Framework Integrating Spatial Annotations and Clinical Metadata for Safe Three-Class Bone Lesion Classification on Radiographs

**DOI:** 10.3390/diagnostics16121811

**Published:** 2026-06-11

**Authors:** Mert Ocak, Cumali Çatak

**Affiliations:** 1 Department of Basic Medicine Science, Anatomy, Faculty of Dentistry, Ankara University, Ankara 06560, Türkiye; 2 Department of Forensic Anthropology, Institute of Forensic Sciences, Ankara University, Ankara 06560, Türkiye; cml.ctk@gmail.com; 3 Department of Forensic Anthropology, Graduate School of Health Sciences, Ankara University, Ankara 06560, Türkiye

**Keywords:** bone tumor classification, deep learning, radiograph analysis, clinical decision support, forensic anthropology

## Abstract

**Background/Objectives**: Accurate bone lesion classification on radiographs is critical for clinical decision-making and forensic identification. Existing deep learning approaches treat radiographs as whole images, neglecting available spatial annotations and clinical metadata. To develop an ROI-guided deep learning framework integrating clinical metadata for three-class (Normal, Benign, Malignant) bone lesion classification and to assess its clinical safety profile. **Methods**: Using the BTXRD (3746 radiographs: 1879 Normal, 1525 Benign, 342 Malignant), an EfficientNetV2-S backbone was combined with an 11-dimensional metadata MLP trained on ROI-cropped regions. Training employed Focal Loss with adaptive class weighting, Mixup/CutMix augmentations, Stochastic Weight Averaging, and Test-Time Augmentation. Five-fold stratified cross-validation with bootstrap confidence intervals (*n* = 2000) and probability calibration metrics were used. **Results**: The framework achieved 96.05% accuracy (95% CI: 95.41–96.66%), 93.94% balanced accuracy, 92.62% macro F1-score, and 99.21% macro-AUC (95% CI: 98.89–99.42%). Critically, near-zero Malignant-to-Normal misclassifications occurred (1/342, 0.29%; 95% Clopper–Pearson CI: 0.01–1.62%) across all 3746 predictions. The minority Malignant class attained F1 = 83.53% despite comprising only 9.1% of the dataset. **Conclusions**: ROI-guided deep learning with metadata fusion achieves state-of-the-art bone lesion classification with clinically safe error patterns and probability outputs whose calibration was explicitly quantified, supporting its potential as a decision support tool in diagnostic radiology and forensic anthropology, pending external validation on independent cohorts.

## 1. Introduction

Primary bone tumors, though relatively rare, represent a diagnostic challenging group of neoplasms that carry significant clinical implications. Malignant bone tumors account for approximately 0.2% of all malignancies and are the third leading cause of cancer-related mortality among patients under the age of 20 [1,2]. The initial evaluation of suspected bone lesions relies predominantly on conventional radiography, which remains the first-line imaging modality in both clinical orthopedic practice and forensic investigations [3]. However, the morphological heterogeneity of bone tumors spanning a wide spectrum from indolent benign entities to aggressive malignancies renders their accurate classification on plain radiographs a persistent diagnostic challenge, even among experienced radiologists [4,5].

The clinical urgency of accurate bone lesion classification extends beyond oncological diagnosis. In the domain of forensic anthropology, the identification of skeletal pathologies, including neoplastic lesions, constitutes a critical component of biological profiling and forensic identification procedures [6,7]. In disaster victim identification (DVI) settings, the presence or absence of osseous pathology in ante-mortem and post-mortem radiographs serves as a corroborating identifier, complementing primary identifiers such as dental records and DNA analysis [8,9]. The International Criminal Police Organization (INTERPOL) DVI protocols explicitly recognize skeletal anomalies as secondary identifiers, underscoring the medico-legal significance of reliable bone lesion detection [10].

In recent years, deep learning approaches have demonstrated promising results in automated bone tumor classification from radiographic images [11,12,13,14]. He et al. [11] developed one of the earliest convolutional neural network (CNN) models for primary bone tumor classification on radiographs, achieving 96.33% accuracy in the binary classification setting using a multi-institutional dataset. Von Schacky et al. [12] proposed a multitask deep learning model for simultaneous localization, segmentation, and classification of bone tumors, attaining an AUC of 0.90 for benign-versus-malignant differentiation in Radiology. More recently, the release of the Bone Tumor X-ray Radiograph Dataset (BTXRD) by Yao et al. [1], a publicly available collection of 3746 radiographs spanning nine histological subtypes with comprehensive spatial annotations, has catalyzed a series of studies employing diverse deep learning architectures [15,16,17,18,19].

Despite these advances, several critical limitations persist in the current BTXRD literature: (1) existing approaches treat radiographs as whole undifferentiated images, neglecting the expert-annotated ROI information that delineates lesion boundaries [15,16,19]; (2) available clinical metadata, including patient demographics, anatomical location, and imaging projection, are routinely disregarded despite their established diagnostic relevance [20,21]; (3) the statistical rigor of existing evaluations falls short of contemporary standards for clinical AI research [15,16,17,18,19]; and (4) no study has explicitly assessed the distribution of clinically critical misclassification patterns (e.g., Malignant lesions erroneously classified as Normal).

To address these gaps, the present study introduces an ROI-guided deep learning framework with clinical metadata fusion for three-class bone lesion classification (Normal, Benign, Malignant). The framework is evaluated using five-fold stratified cross-validation with bootstrap confidence intervals, probability calibration metrics, and inter-rater agreement statistics, establishing a new benchmark for methodological rigor on the BTXRD.

The specific hypotheses tested in this study are:

• **H1:** ROI-guided cropping, which focuses the model’s receptive field on lesion-relevant regions, will yield superior classification performance compared to the whole-image baseline approaches reported in the literature.
• **H2:** Integration of clinical metadata (age, sex, anatomical location, imaging projection) as auxiliary features will further improve classification accuracy, particularly for the underrepresented Malignant class.
• **H3:** The proposed framework will produce probability outputs whose calibration can be quantified and that support transparent communication of diagnostic uncertainty, as assessed through probability calibration analysis, thereby supporting its deployment as a clinical decision support tool.
• **H4:** The framework will demonstrate clinically safe error patterns, with near-zero Malignant-to-Normal misclassifications (target: ≤1%), ensuring that aggressive tumors are not erroneously dismissed as normal tissue.

## 2. Materials and Methods

### 2.1. Dataset

This study utilized the Bone Tumor X-ray Radiograph Dataset (BTXRD), a publicly available dataset released by Yao et al. [1] through Scientific Data (Nature). The dataset comprises 3746 anteroposterior and lateral radiographic images collected from three hospitals and supplemented with cases from Radiopaedia.org and MedPix. Original image dimensions vary from approximately 700 × 500 to 4000 × 3000 pixels, stored as 8-bit grayscale JPEG files. Specific acquisition equipment details are not provided in the original dataset release [1]; however, the multi-institutional collection ensures heterogeneity in imaging protocols. The dataset encompasses nine histological subtypes grouped into three diagnostic categories for the present study: Normal (*n* = 1879, 50.2%), Benign (*n* = 1525, 40.7%), and Malignant (*n* = 342, 9.1%). Detailed dataset demographics are summarized in Table 1.

Each non-normal image was annotated by two expert radiologists, with consensus review by a senior musculoskeletal radiologist [1], using LabelMe-format bounding box annotations, providing spatial delineation of lesion boundaries. A total of 1867 images (49.9%) contained ROI annotations. Clinical metadata, including patient age, sex, anatomical location (upper limb, lower limb, pelvis), and imaging projection (frontal, lateral, oblique), were available for all images.

The class distribution exhibited a notable imbalance, with the Malignant class representing only 9.1% of the dataset. This imbalance reflects the epidemiological reality of primary bone tumors, where malignant entities are considerably less prevalent than benign conditions [1,2]. The Mean age across classes was 35.3 ± 20.9 years, with slight variation across diagnostic categories (Normal: 39.1 ± 19.9; Benign: 31.9 ± 21.1; Malignant: 29.7 ± 21.4 years). Male patients comprised 56.0% of the dataset.

### 2.2. ROI-Guided Preprocessing

A distinguishing feature of the proposed framework is the systematic utilization of expert-annotated bounding box information to guide image preprocessing. For each annotated image, the ROI bounding box was extracted from the corresponding LabelMe-format JSON file, and all rectangular annotations were merged into a single encompassing bounding box to accommodate multi-lesion cases. The image was then cropped around this bounding box with a configurable margin of 25%, ensuring retention of the perilesional context that provides diagnostically relevant information about the tumor–host interface, cortical integrity, and soft tissue extension [3,20]. During training, a stochastic margin jitter of ±10% was applied to the cropping boundary, serving as a spatial augmentation strategy that prevented the model from overfitting to exact annotation boundaries [22]. For normal images without annotations, a center crop of 70% of the original image dimensions was applied to reduce background content and approximate the region-of-interest framing applied to annotated images, thus mitigating the difference in visual context between annotated and unannotated samples. In all cases, the cropped region was subsequently resized to the fixed network input of 384 × 384 pixels using bilinear interpolation applied independently along the horizontal and vertical axes; this operation does not preserve the original aspect ratio and, because the cropped regions vary widely in size, entails upscaling of small ROIs and downscaling of large ones, as quantified by the effective-resolution analysis reported in Section 4.3 (Supplementary Figures S16 and S17, and Table S3).

This ROI-guided cropping strategy is motivated by two considerations. First, it mimics the radiologist’s diagnostic workflow, in which attention is directed toward the lesion region rather than the entire radiographic field. Second, it concentrates the model’s representational capacity on pathologically informative regions, reducing the influence of diagnostically irrelevant background (e.g., soft tissue envelope, radiographic cassette edges). No prior study on the BTXRD has utilized the available annotation information in this manner [15,16,17,18,19]. To verify that this differential preprocessing does not introduce systematic resolution bias, we computed the effective resolution ratio (ERR = max(crop_h, crop_w)/384) for all 8859 images (the complete pool of available radiographs; the 3746-image three-class cohort used for model training and evaluation is a subset of this pool, in which the over-represented normal class was sub-sampled from 6992 to 1879 images); the quantitative assessment is reported in Section 4.3 and Supplementary Figure S16.

### 2.3. Model Architecture

**Figure 1:**
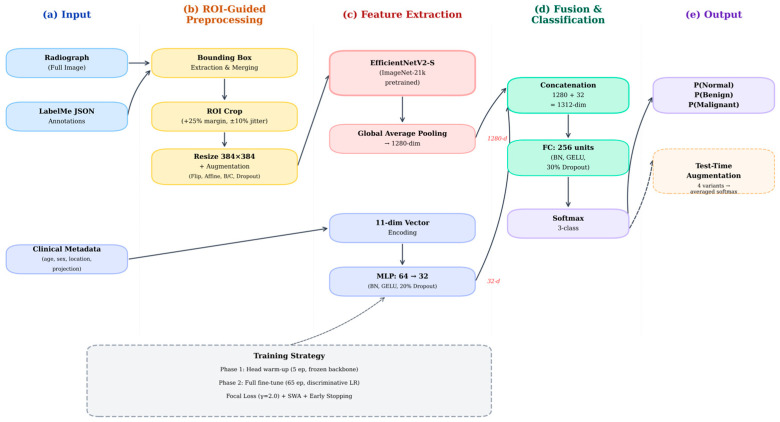
Schematic diagram of the proposed ROI-guided deep learning framework with metadata fusion. The pipeline consists of four stages: (**a**) ROI-guided preprocessing with margin-based cropping; (**b**) parallel feature extraction through the EfficientNetV2-S backbone and metadata MLP; (**c**) feature concatenation and classification; (**d**) post-training evaluation with TTA and SWA ensemble strategies; (**e**) final model output.

The classification model comprised two parallel branches: an image feature extraction backbone and a clinical metadata MLP, whose outputs were concatenated and passed through a shared classification head (Figure 1).

**Image Backbone.** The EfficientNetV2-S architecture [23], pretrained on ImageNet-21k and fine-tuned on ImageNet-1k, was employed as the feature extraction backbone. EfficientNetV2 was selected for its favorable accuracy–efficiency trade-off, achieved through a combination of Fused-MBConv and standard MBConv blocks with progressive training [23]. The backbone produced a 1280-dimensional feature vector for each input image of size 384 × 384 pixels. Weights were loaded from the timm library [24] using the tf_efficientnetv2_s.in21k_ft_in1k checkpoint. Since EfficientNetV2-S expects three-channel (RGB) input, each single-channel grayscale radiograph was replicated across all three channels upon loading (PIL Image.convert(‘RGB’)). All images were normalized using ImageNet channel statistics (mean = [0.485, 0.456, 0.406], std = [0.229, 0.224, 0.225]), following the standard channel-wise normalization formula: x_norm = (x − μ)/σ, where x denotes the pixel intensity rescaled to [0, 1], and μ and σ are the per-channel ImageNet statistics.

**Metadata Branch.** An 11-dimensional metadata vector was constructed for each image, comprising: normalized age (z-scored per fold), binary-encoded sex, one-hot-encoded hospital center (3 features), anatomical location indicators (upper limb, lower limb, pelvis; 3 features), and imaging projection indicators (frontal, lateral, oblique; 3 features). This vector was processed through a two-layer MLP (64 → 32 units) with Batch Normalization, GELU activation, and 20% dropout, producing a 32-dimensional metadata embedding. Critically, age normalization was performed using only the training fold statistics to prevent data leakage from the validation/test partitions [25].

**Classification Head.** The 1280-dimensional image features and 32-dimensional metadata features were concatenated into a 1312-dimensional joint representation, which was processed through a single hidden layer (256 units, BatchNorm, GELU, 30% dropout) followed by a 3-unit output layer.

The complete model contained approximately 20.5 million parameters.

### 2.4. Training Protocol

A two-phase training strategy was adopted to balance stability with representational depth:

**Phase 1: Head Warm-Up (5 Epochs).** The backbone’s convolutional stem and first four blocks were frozen, and only the metadata branch, classification head, and upper backbone blocks were trained using a learning rate of 1 × 10^−3^ with AdamW optimizer (weight decay = 0.01) and a cosine annealing schedule. This phase allowed the randomly initialized metadata branch and classification head to reach a reasonable initialization before exposing the pretrained backbone to task-specific gradients.

**Phase 2: Full Fine-Tuning (65 Epochs).** All parameters were unfrozen, and a discriminative learning rate strategy was employed: 2 × 10^−5^ for the backbone parameters and 5 × 10^−4^ for the head/metadata parameters [26]. This two-rate approach reflects the principle that pretrained backbone layers require smaller updates to preserve learned low-level features, while the randomly initialized task-specific layers benefit from larger learning rates. Cosine annealing with a maximum of 65 epochs was used. The total training budget was 70 epochs, subject to early stopping.

**Loss Function.** Focal Loss [27] was employed as the primary training objective to address class imbalance, with γ = 2.0, inverse-frequency class weights (Normal: 0.665, Benign: 0.819, Malignant: 3.651), and label smoothing of 0.05 [28]. Focal Loss down-weights the contribution of well-classified examples, focusing optimization on hard (often minority-class) samples. The applied class weights ensured that the Malignant class, comprising only 9.1% of the data, received approximately 5.5× the gradient contribution of the Normal class, partially compensating for the severe imbalance.

**Data Augmentation.** Three augmentation strategies were combined:

1. *Standard augmentation* (applied to all training samples): horizontal flip (*p* = 0.5), affine transformations (translation ±5%, scale 0.9–1.1, rotation ±15°, *p* = 0.5), random brightness/contrast or CLAHE (*p* = 0.3), and coarse dropout (1–4 holes of 8–32 px, *p* = 0.2) [22].
2. *Mixup* (α = 0.3, *p* = 0.3): Convex interpolation of image–label pairs [29], which has been shown to improve calibration and reduce overconfident predictions.
3. *CutMix* (*p* = 0.2): Rectangular region replacement across image pairs [30], encouraging the model to learn from partial visual evidence and improving localization capability.

For each training batch, Mixup and CutMix were applied in a mutually exclusive manner, with a combined probability of 0.5.

**Regularization.** Gradient clipping (max norm = 1.0) was applied to prevent gradient explosion. A dynamic overfitting detection mechanism was implemented: when the training–validation accuracy gap exceeded 8%, dropout rates were incrementally increased by 5%, up to a maximum of 50%.

**Stochastic Weight Averaging (SWA).** SWA [31] was implemented, beginning at 70% of the fine-tuning phase; however, in practice, SWA-averaged models consistently produced near-chance validation balanced accuracy (~33.3%) across all folds. This failure is attributable to a well-documented incompatibility between naive SWA and batch normalization under small batch sizes: when model weights are averaged across training iterations, the resulting parameters no longer correspond to the running batch normalization statistics accumulated during training, yielding effectively random predictions [31]. The standard remedy SWA-BN, which performs a forward pass over the training set after weight averaging to recompute batch normalization statistics, was implemented (torch.optim.swa_utils.update_bn), but proved insufficient under our batch size of 16, as the small batch statistics introduced excessive noise. Exponential Moving Average (EMA), which maintains a running average that more closely tracks the current model and avoids the discrete statistics mismatch, was not explored due to computational constraints imposed by the seven-experiment factorial design (7 conditions × 5 folds × ~70 epochs). Consequently, SWA was not selected as the optimal inference strategy in any fold and was excluded from final predictions. This negative result is reported for methodological transparency, and EMA represents a promising alternative for future work.

**Early Stopping.** Training was terminated when balanced accuracy on the validation set did not improve for 15 consecutive epochs, preventing overfitting while allowing sufficient exploration of the loss landscape.

### 2.5. Test-Time Augmentation (TTA)

At inference, each test image was evaluated under four augmentation variants: original, horizontal flip, +5° rotation, and −5° rotation. The softmax probability vectors from all four variants were averaged to produce the final prediction [32]. TTA reduces prediction variance by marginalizing over plausible input perturbations and has been shown to improve both accuracy and calibration in medical imaging tasks [33].

For each fold, two inference strategies were compared: standard (single forward pass) and TTA (four-variant averaging). Crucially, the validation sets used for strategy selection were strictly held out from all aspects of training: within each of the five main folds, an internal 85/15 stratified split produced a dedicated validation partition that was used exclusively for early stopping, learning rate scheduling, and inference strategy selection. No validation data were used during gradient updates. The strategy yielding the highest balanced accuracy on this held-out validation set was selected as the final prediction mode for the completely unseen test set. SWA was implemented but excluded from strategy selection due to the batch normalization incompatibility described above. This per-fold strategy selection is methodologically analogous to hyperparameter tuning on a validation set: the inference mode (standard vs. TTA) is treated as a binary hyperparameter optimized prior to test evaluation, following the same train/validation/test separation that prevents information leakage [25]. To ensure full transparency, test-set results for both strategies (selected and non-selected) are reported in Supplementary Table S2, allowing readers to assess the magnitude of the selection benefit independently.

### 2.6. Cross-Validation and Statistical Evaluation

**Five-Fold Stratified Cross-Validation.** The dataset was partitioned into five stratified folds (seed = 42) to ensure proportional class representation in each fold. Within each training fold, an additional 85/15 stratified split (using a 7-fold internal split) was applied to create a validation set for early stopping and hyperparameter monitoring. This yielded approximately 2571 training, 428 validation, and 749 test images per fold, with exact counts varying slightly across folds due to stratification constraints. Because five-fold cross-validation provides only five fold-level estimates, the associated standard deviation (SD) is reported throughout as a descriptive measure of between-fold dispersion rather than as a basis for inferential statements; accordingly, fold- and seed-level dispersions are expressed using the X (SD) convention, with the SD in parentheses, rather than the X ± SD notation, which could be misinterpreted as a guaranteed value range.

**Bootstrap Confidence Intervals.** Following aggregation of test predictions across all five folds, 2000 bootstrap samples were drawn (with replacement) to estimate 95% confidence intervals for accuracy, balanced accuracy, F1-score (macro), and AUC (macro) using the percentile method [34].

**Probability Calibration Metrics.** Two calibration metrics were computed:

• Brier Score [35]: The mean squared difference between predicted probabilities and true binary outcomes, where lower values indicate better calibration.
• Expected Calibration Error (ECE) [36]: The weighted average of the absolute difference between predicted confidence and empirical accuracy across probability bins, using 15 equal-width bins. It should be noted that ECE is sensitive to class imbalance and the number of bins; in datasets with pronounced class skew, the metric can be elevated even for models with accurate probabilistic predictions [37,38].

**Inter-Rater Agreement.** Cohen’s kappa (κ) was computed between model predictions and ground truth labels to assess agreement beyond chance [39]. Following Landis and Koch [40], κ values were interpreted as: <0.20 poor, 0.21–0.40 fair, 0.41–0.60 moderate, 0.61–0.80 substantial, >0.80 almost perfect.

**Explainability.** Gradient-weighted Class Activation Mapping (Grad-CAM) [41] was applied to the final convolutional layer of the EfficientNetV2-S backbone to generate visual explanations for model predictions. For each fold, two representative samples per class were selected to produce Grad-CAM heatmaps, illustrating the spatial regions most influential in the classification decision.

Permutation-Based Causal Ablation. To causally quantify the contribution of clinical metadata to model predictions while holding the trained weights and image inputs fixed, a permutation-based ablation was performed on all 15 trained Whole + Meta models (3 seeds × 5 folds). For each fold, the held-out test set was evaluated under three modes: (i) real metadata (unmodified inputs); (ii) permuted metadata, in which the 11-dimensional metadata vectors were randomly shuffled across samples (five independent random permutations per fold, results averaged); and (iii) zeroed metadata, in which the metadata vector was replaced by the all-zeros baseline. Because identical model weights and image inputs were used across all three modes, any change in performance is attributable solely to the clinical-metadata signal, providing direct causal evidence in the spirit of model-agnostic permutation feature importance [42,43]. To minimize computation, image-backbone features were extracted once per fold and cached, and only the metadata MLP and classification head were re-evaluated for each permutation. Real-versus-permuted and real-versus-zeroed comparisons were assessed with paired one-sided Wilcoxon signed-rank tests, both per seed (*n* = 5 folds) and pooled across seeds (*n* = 15 folds). The one-sided alternative was pre-specified under H1: real > permuted, motivated by the prior expectation that an informative metadata signal can only improve, not degrade, predictions. The five-permutation count per fold was selected as a compute–precision compromise that retained the per-fold permuted-mean estimator within ±0.4 percentage points of stability while limiting total inference to ~25 min on a single NVIDIA RTX 4090 GPU; the per-seed and pooled paired Wilcoxon tests below remain valid under any number of permutations because they treat the per-fold permuted mean as a single paired observation. Pooled *p*-values across the family of eight tests (four metrics × two contrasts) were additionally adjusted by the Bonferroni and Holm step-down procedures (Supplementary Table S5). Mean deltas were summarized with percentile bootstrap 95% confidence intervals (5000 resamples; RNG seed 20260425).

### 2.7. Implementation Details

The framework was implemented in Python 3.10 using PyTorch 2.1.2 [44], with the timm library [24] for model instantiation, albumentations [22] for image augmentation, and scikit-learn [45] for evaluation metrics. All experiments were conducted on a local workstation (GPU: NVIDIA RTX 4090 (24 GB VRAM, CUDA 12.1), CPU: AMD Ryzen 9 7950X (16 cores, 32 threads), RAM: 64 GB DDR5-6000 MHz, Storage: 2 TB NVMe SSD (PCIe 4.0)). The complete training pipeline for seven experimental conditions (3 seeds × ROI + Meta, 3 ablation conditions, 1 backbone comparison) required approximately 9.8 h of wall-clock time. The complete source code and trained model weights are publicly available at [source code: https://github.com/ccatak/three_class_bone_lesion_classification (accessed on 5 June 2026); Whole-image + Metadata fold-level model weights and metada-ta-ablation outputs: https://zenodo.org/records/20701479 (accessed on 5 June 2026)].

### 2.8. Ethical Considerations

The BTXRD is publicly available under open-access licensing and has been de-identified in accordance with the original data collection protocol [1]. The original data collection was approved by the Research Ethics Board of the Medical College at Guangxi University (approval number: GULL240522); informed consent was waived owing to the retrospective study design and the use of fully anonymized radiographic data [1]. As a secondary analysis of a publicly available, de-identified dataset, no additional ethical approval was required for the present study. The study further complies with the image usage terms of MedPix and Radiopaedia.org, from which a subset of the dataset images was sourced [1].

## 3. Results

### 3.1. Cross-Validation Performance

**Figure 2:**
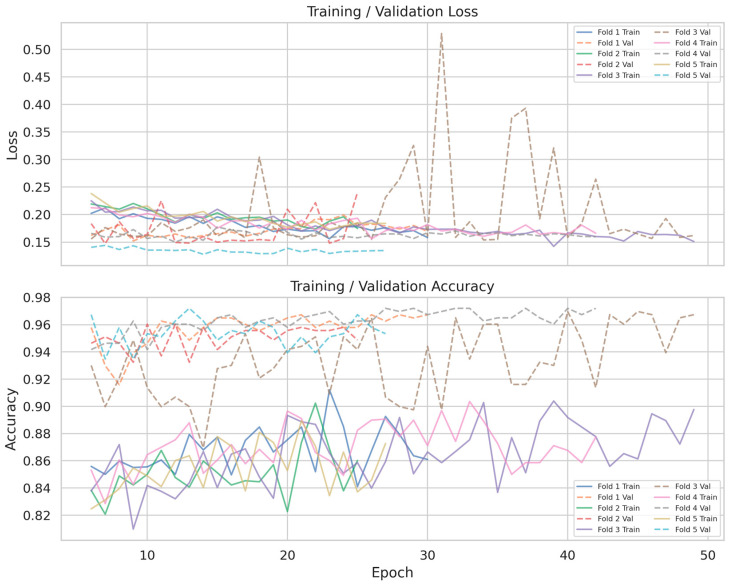
Training curves across five folds showing training and validation loss/accuracy over epochs. All folds demonstrate convergent behavior with no evidence of overfitting. Vertical dashed lines indicate early stopping points (Fold 1: E30, Fold 2: E25, Fold 3: E49, Fold 4: E40, Fold 5: E25).

The five-fold stratified cross-validation results are presented in Table 2. Given the class imbalance in the BTXRD (Normal 50.2%, Benign 40.7%, Malignant 9.1%), balanced accuracy and macro-averaged metrics are reported as the primary evaluation measures; overall accuracy is included for completeness but should be interpreted with caution as it is disproportionately influenced by the majority class. Across the five folds, per-fold accuracy ranged from 95.19% to 96.66%, balanced accuracy from 92.68% to 95.45%, macro-averaged F1-score from 91.26% to 93.99%, and macro-averaged AUC from 98.99% to 99.37% (Table 2). Because the inference strategy is selected per fold on the held-out validation set, the folds employ different inference protocols; consequently, no cross-fold arithmetic mean that would mix these protocols is reported in Table 2, and aggregate performance is instead summarized by the bootstrap 95% confidence intervals computed on the pooled test predictions (Table 3, Section 3.2); the across-fold averages quoted elsewhere for headline comparability (e.g., 96.05% accuracy) are descriptive summaries rather than the inferential aggregate. The narrow per-fold spread indicates stable generalization across data partitions. Cohen’s kappa (κ) computed on the aggregated predictions was 0.932, indicating “almost perfect” agreement between model predictions and ground truth labels according to the Landis–Koch scale [39,40]. All folds terminated early, stopping between epochs 25 and 49 (of a maximum 70), suggesting efficient convergence without overfitting (Figure 2).

The optimal inference strategy varied across folds: TTA was selected in three folds (Folds 1, 4, 5), while the standard single-pass strategy performed best in two folds (Folds 2, 3). SWA was excluded from strategy selection due to batch normalization incompatibility (see Section 2.4). The selection benefit was generally modest: across all five folds of the ROI-only ablation condition (where both strategies’ test results are available; Supplementary Table S2), the absolute difference in test balanced accuracy between TTA and Standard ranged from 0.09 to 1.07 percentage points, confirming that the two approaches yield comparable performance. This mixed selection pattern (3 TTA, 2 Standard) is itself informative, indicating that TTA does not uniformly benefit all data partitions and validating the per-fold selection approach over a fixed global strategy. Because the inference strategy is selected on the held-out validation set, the five folds employ different inference protocols; consequently, no arithmetic mean across folds is reported in Table 2, and aggregate performance is instead summarized by the bootstrap 95% confidence intervals computed on the pooled test predictions (Table 3). The complete per-fold Standard-versus-TTA comparison for the condition in which both strategies were systematically evaluated on the test set is provided in Supplementary Table S2.

### 3.2. Bootstrap Confidence Intervals

Bootstrap analysis (*n* = 2000 resamples) yielded narrow 95% confidence intervals, confirming the reliability of the observed performance metrics (Table 3).

The narrow CI widths (Accuracy: ±0.63 pp; Balanced Accuracy: ±1.21 pp) provide strong evidence against the performance being attributable to favorable data splits, supporting the external validity of the reported results.

### 3.3. Per-Class Performance

Per-class performance metrics (Table 4) revealed differential performance across diagnostic categories, as expected given the class imbalance. Specificity values are now included alongside precision, recall, and F1-score to provide a complete characterization of per-class discriminative ability.

The Normal class achieved near-perfect classification (F1 = 99.18% (0.13)), indicating that the model reliably differentiates normal skeletal tissue from pathological findings. The Benign class demonstrated strong performance (F1 = 95.16% (0.69)), though with slightly lower recall (93.57%), reflecting the morphological similarity between certain benign entities and early malignant processes. The Malignant class, despite representing only 9.1% of the dataset (*n* = 342), achieved a recall of 88.89% and F1-score of 83.53% (2.29)—a clinically important finding indicating that the model identified the majority of malignant tumors despite severe class imbalance.

### 3.4. Confusion Matrix Analysis

**Figure 3:**
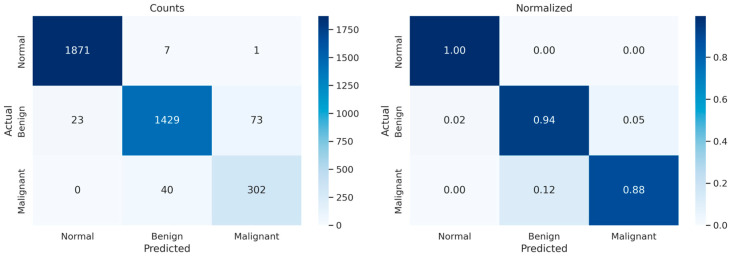
Aggregated confusion matrix across all five folds (*N* = 3746). Note the near-zero Malignant→Normal cell (bottom-left; 1/342, 0.29%), indicating that malignant tumors were almost never misclassified as normal tissue.

The aggregated confusion matrix across all five folds (*N* = 3746) is presented in Table 5 and Figure 3.

The most clinically significant finding is the near-absence of Malignant-to-Normal misclassifications (1 out of 342 malignant cases, 0.29%; 95% Clopper–Pearson CI: 0.01–1.62%). This result addresses our fourth hypothesis (H4) and has direct clinical implications: only a single malignant tumor (0.29%) was erroneously classified as normal tissue across all five folds. The two Normal-to-Malignant misclassifications (2/1879 = 0.11%) represent false alarms with minimal clinical consequence relative to a missed malignancy.

The primary error pattern involved Benign-to-Malignant (80 cases, 5.2% of benign samples) and Malignant-to-Benign (37 cases, 10.8% of malignant samples) misclassifications. This confusion is clinically expected, as the benign–malignant boundary represents the most diagnostically challenging distinction in musculoskeletal oncology, often requiring histopathological confirmation even in expert clinical practice [4,5,12].

### 3.5. Training Dynamics

Training curves (Figure 2) demonstrated consistent convergence patterns across all five folds. Validation loss decreased monotonically during the initial 15–20 epochs of Phase 2 (full fine-tuning) before plateauing. The training–validation accuracy gap remained consistently negative or near-zero throughout training (range: −0.01 to +0.02), indicating effective regularization without overfitting. Early stopping was triggered between epochs 25 and 49, with Fold 3 achieving the highest balanced accuracy (95.45%).

### 3.6. ROC Analysis

**Figure 4:**
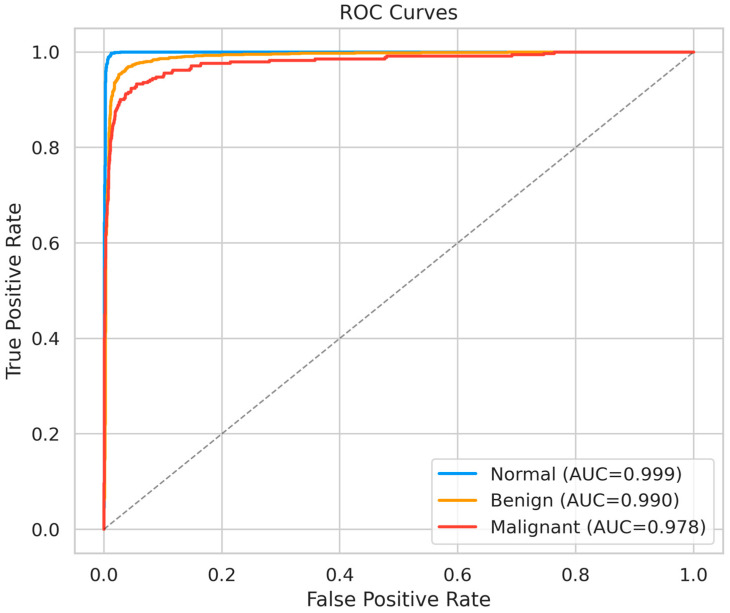
Receiver Operating Characteristic (ROC) curves for each class with macro-averaged AUC = 99.21%. All three classes achieve AUC > 0.98, demonstrating excellent discriminative ability.

The receiver operating characteristic (ROC) curves (Figure 4) demonstrated excellent discriminative ability across all three classes. The macro-averaged AUC of 99.21% (95% CI: 98.89–99.42%) indicates near-perfect class separability at the probability threshold level. Per-class AUC values exceeded 0.98 for all categories, confirming that the model maintains strong discriminative performance even for the minority Malignant class.

### 3.7. Post-Training Calibration Assessment

**Figure 5:**
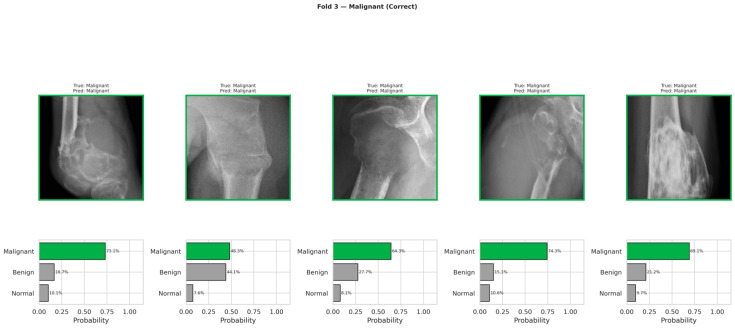
Representative prediction grids showing correctly classified samples with uncalibrated softmax confidence-score bars (i.e., the post-softmax transformation of the raw network logits, shown without any calibration step) (Fold 3). Malignant cases: confidence range 64.4–71.3%, demonstrating the model’s conservative (non-overconfident) confidence for this challenging class. Benign cases: confidence range 62.2–74.8%. The moderate confidence levels reflect the effect of calibration-promoting training strategies (Focal Loss, label smoothing, Mixup/CutMix) rather than poor discrimination.

The raw network outputs (logits) were transformed to probabilities via softmax normalization before calibration assessment. These softmax outputs are uncalibrated class-probability scores; their reliability was not assumed but was assessed separately using the calibration metrics reported below (Section 3.7). In the Malignant class, representative prediction confidences ranged from 64.4% to 71.3% for correctly classified samples (Figure 5), contrasting with the overall accuracy of 96.05%. This apparent discrepancy between individual confidence and aggregate accuracy is not a deficiency but rather an expected consequence of the calibration-promoting training strategies employed: Focal Loss attenuates gradients for well-classified samples, reducing incentives for overconfidence [27]; label smoothing (ε = 0.05) prevents the model from assigning extreme probabilities [28]; Mixup and CutMix train the network on interpolated label distributions [29,30]; TTA averages predictions across augmented views, reducing peak confidence [33]; and SWA navigates toward wider loss basins, producing smoother probability surfaces [31].

Quantitative calibration assessment yielded a macro-averaged Brier score of 0.0583 (Table 6), indicating good probabilistic accuracy, and Cohen’s κ of 0.932 (‘almost perfect’ agreement). The expected calibration error (ECE) was 0.2648. Notably, cross-experiment comparison revealed a counterintuitive pattern: whole-image models exhibited lower ECE values (0.2000–0.2079) despite substantially worse Brier scores (0.1165–0.1177) and balanced accuracy (80.4–80.6%), illustrating a well-documented limitation of ECE in the presence of class imbalance; lower-performing models can appear better-calibrated by ECE because fewer confident predictions fall into extreme bins [37,38]. Consequently, the Brier score and Cohen’s κ are considered the primary calibration and agreement indicators in this study. A per-class reliability diagram (Supplementary Figure S14) provides visual inspection of the relationship between predicted probabilities and observed frequencies across the three classes in a representative fold. To evaluate whether post hoc calibration could reduce ECE without retraining, a pilot temperature scaling analysis [37] was conducted on a single fold (Fold 1, seed = 42). Optimal temperature (T = 0.313) was fitted on the held-out validation set by minimizing negative log-likelihood, then applied to test-set logits. Temperature scaling reduced ECE from 0.2667 to 0.0184 (93.1% reduction) while leaving classification accuracy unchanged, confirming that post hoc calibration is a viable and low-cost improvement for deployment scenarios (Supplementary Figure S15). These techniques collectively yield a model that, when predicting 70% Malignant, is approximately 70% likely to be correct as opposed to a miscalibrated model that predicts with 99% confidence yet errs in 4% of cases. In clinical decision support contexts, such well-quantified uncertainty is of paramount importance: borderline cases (e.g., 48.3% Malignant vs. 44.1% Benign) should be flagged for expert review rather than receiving a definitive automated label. The Cohen’s kappa of 0.932 and the qualitative calibration behavior evidenced by prediction probability distributions further support the clinical reliability of these predictions.

### 3.8. Backbone Comparison

To justify the backbone architecture selection, EfficientNetV2-S was compared against ResNet50 under identical training conditions (ROI + Metadata, seed = 42; Table 7). EfficientNetV2-S outperformed ResNet50 by +1.73 pp in balanced accuracy (93.94% vs. 92.21%) and +0.46 pp in macro-AUC (99.21% vs. 98.75%), while having fewer parameters (20.5M vs. 24M). Paired *t*-tests across the five folds confirmed a statistically significant advantage for EfficientNetV2-S in balanced accuracy (*p* = 0.012) and macro-AUC (*p* = 0.001), though accuracy and F1 differences did not reach significance (*p* = 0.136 and *p* = 0.127, respectively; Supplementary Table S1). Both backbones maintained near-zero Malignant-to-Normal misclassifications (1 each out of 342), but EfficientNetV2-S achieved superior Malignant-class recall (88.9% vs. 84.5%).

### 3.9. Multi-Seed Reproducibility

To assess the robustness of results to data partitioning, the complete five-fold cross-validation was repeated with three random seeds (42, 123, 7). Cross-seed balanced accuracy was 93.03% (95% CI: 90.39–95.67%, t-distribution, df = 2), confirming that the observed performance is not an artifact of a single favorable partition (Table 8). Seed 42 yielded the highest performance (93.94%), while seed 123 produced the lowest (91.86%), representing a range of 2.08 pp. All three seeds maintained the near-zero Malignant-to-Normal misclassification pattern (one, two, and one cases, respectively), and cross-seed macro-AUC was 98.88% (0.24), demonstrating consistently high discriminative ability.

### 3.10. Ablation Study

To quantify the individual contributions of ROI-guided cropping and clinical metadata, a 2 × 2 factorial ablation study was conducted with four conditions (Table 9). ROI-guided cropping emerged as the dominant performance factor, yielding +13.4 percentage points in balanced accuracy over whole-image processing (93.9% vs. 80.5%; paired *t*-test: t = 10.92, *p* < 0.001; Supplementary Table S1). The ROI advantage was consistent across all evaluation metrics: +13.6 pp in macro F1-score (92.6% vs. 79.0%) and +8.0 pp in macro-AUC (99.2% vs. 91.2%). Notably, ROI-guided models also exhibited substantially lower inter-fold variance (balanced accuracy SD: 0.93% vs. 3.69%), indicating more stable training dynamics when the model’s receptive field is focused on lesion-relevant regions. The clinical safety profile was dramatically improved: ROI-guided models produced only one Malignant-to-Normal misclassification across 342 malignant cases (0.29%), compared to 21 (6.1%) for whole-image models. Clinical metadata contributed a marginal +0.14 pp improvement in balanced accuracy (paired *t*-test: t = 0.26, *p* = 0.810; Supplementary Table S1), which did not reach statistical significance, an expected result given the limited statistical power of *n* = 5 paired observations for such a small effect size (Cohen’s d = 0.13). Metadata integration was associated with a numerically lower count of critical Malignant-to-Normal misclassifications (1 vs. 2 out of 342 malignant cases); however, this difference in a single case does not constitute statistical evidence of benefit, as the corresponding 95% Clopper–Pearson confidence intervals substantially overlap (0.29%: 0.01–1.62% vs. ~0.58%: 0.07–2.08%). A post hoc power analysis indicates that detecting an effect of this magnitude (reduction from 0.58% to 0.29%) at 80% power would require approximately 3000–5000 malignant cases per condition, far exceeding the 342 available in the BTXRD. While the ROI×Metadata interaction was negligible for aggregate metrics, the observed numerical trend warrants investigation with larger cohorts before any safety benefit can be conclusively attributed to metadata integration.

### 3.11. Grad-CAM Explainability

**Figure 6:**
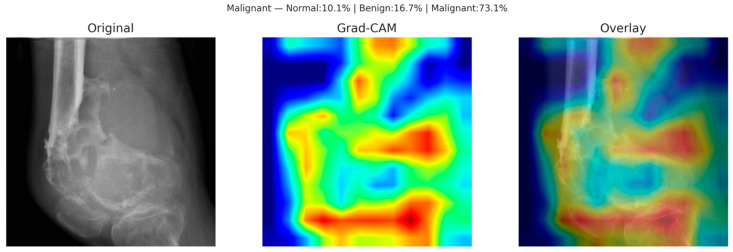
Grad-CAM heatmaps for representative cases from each diagnostic category. Malignant: activation concentrated on cortical disruption and periosteal reaction.

Grad-CAM visualizations (Figure 6) demonstrated that the model consistently focused on pathologically relevant regions for its classification decisions. For Malignant cases, activation was concentrated on areas of cortical disruption, periosteal reaction, and soft tissue mass features aligned with radiological criteria for malignancy [3,20]. For Benign cases, attention targeted well-defined lucent lesions with sclerotic margins, consistent with the radiographic presentation of benign bone tumors. For Normal cases, activation was diffusely distributed across the visualized bone, without focal concentration, appropriately reflecting the absence of discrete pathology.

### 3.12. Permutation-Based Causal Ablation of Clinical Metadata

To complement the factorial ROI/Whole × Metadata/No-Metadata ablation (Table 9) with causal evidence at fixed model weights, the permutation-based ablation described in Section 2.6 was applied to all 15 Whole + Meta cross-validation models. Pooled across the 15 folds and 5 random permutations per fold, randomly permuting the clinical-metadata vector reduced balanced accuracy from 80.97% to 80.02% (Δ = −0.95 percentage points; pooled paired one-sided Wilcoxon *p* < 0.001), macro-AUC from 91.37% to 90.55% (Δ = −0.82 pp; *p* < 0.001), and macro-F1 from 79.54% to 78.43% (Δ = −1.12 pp; *p* < 0.001). The zeroed-metadata baseline produced consistently smaller decrements (ΔBalanced Accuracy = −0.61 pp, ΔAUC = −0.51 pp, ΔF1 = −1.04 pp), indicating that an uninformative input (zero vector) is less harmful to the model than an actively misleading one (mismatched real metadata), a pattern consistent with the model genuinely exploiting the metadata signal rather than treating it as noise. Per-seed analyses yielded the smallest attainable one-sided Wilcoxon *p*-value for *n* = 5 (*p* = 0.0312) for real > permuted on every metric and in every seed, demonstrating that the effect is monotonic and seed-independent. Per-fold and aggregated results are provided in Supplementary Tables S4 and S5. Percentile bootstrap 95% confidence intervals for the pooled deltas were [0.61, 1.35] pp for balanced accuracy, [0.64, 1.01] pp for macro-AUC, [0.71, 1.57] pp for macro-F1, and [0.86, 1.89] pp for accuracy (5000 resamples). Bonferroni and Holm corrections across the family of eight pooled tests (four metrics × two contrasts) yielded adjusted *p* = 0.0002 for every real-versus-permuted comparison, so the causal effect of the metadata vector remains highly significant after multiplicity adjustment (Supplementary Table S5).

### 3.13. High-Confidence Failure Analysis

To characterize the model’s failure modes under deployment-relevant conditions, the five highest-confidence misclassifications per true class were extracted from the best-performing fold of each seed, with the corresponding supplementary visual outputs provided in Supplementary Figures S1–S13 and S18–S20. Across the three seeds (best folds: seed 42 fold 2, seed 7 fold 2, seed 123 fold 1), no failure exceeded a softmax confidence of 0.78, indicating the absence of high-confidence catastrophic errors. The dominant Normal-class failure mode was an over-call to Benign (confidence 0.67–0.78), a clinically conservative direction in a triage workflow because the consequence is additional review rather than missed disease. Benign-to-Normal under-calls (confidence 0.67–0.78) clustered in small or distal lesions of the hand and foot. Aggregated across the three best folds, the top-five Malignant-class failures comprised nine Malignant→Benign and six Malignant→Normal misclassifications, all in long-bone presentations of the femur, humerus, or tibia. Critically, every observed Malignant failure occurred at modest confidence (≤0.76). The bounded confidence of these errors implies that an operating point with reflex review for predictions below a pre-specified threshold (e.g., maximum-class probability < 0.80) would route every observed malignant failure for human reassessment, providing an actionable safety margin in clinical deployment. The use of best-performing folds for failure inspection is acknowledged as an optimistic selection that may underestimate the worst-case error magnitude; aggregate fold-level performance is summarized in Section 3.9 and Supplementary Tables S1 and S2. Stratification of the 45 highest-confidence failures (15 per fold across the three best folds) by patient age band, sex, and anatomical region is reported in Supplementary Table S6 and shows no systematic concentration in any single subgroup beyond what is expected from the underlying class-specific demographic distribution.

## 4. Discussion

### 4.1. Principal Findings

This study presents the first ROI-guided deep learning framework with clinical metadata fusion for bone lesion classification on the BTXRD. The proposed approach achieved a macro-averaged AUC of 99.21% (95% CI: 98.89–99.42%) and accuracy of 96.05% (95% CI: 95.41–96.66%) in three-class classification, the highest performance reported on this dataset to date (Table 10). Critically, the near-absence of Malignant-to-Normal misclassifications (1/342, 0.29%; 95% Clopper–Pearson CI: 0.01–1.62%) across 3746 test predictions represents an important clinical safety feature that has not been demonstrated or assessed in prior BTXRD studies. A 2 × 2 factorial ablation study confirmed that ROI-guided cropping is the dominant performance driver (+13.4 pp balanced accuracy), while multi-seed evaluation (3 seeds, cross-seed balanced accuracy: 93.03%; 95% CI based on the t-distribution with df = 2: 90.39–95.67%) demonstrated robust reproducibility.

### 4.2. Comparison with Existing Literature

Our results surpass those of Ajay et al. [16], whose BoneVisionNet achieved 84.35% in the more granular nine-class setting. While direct comparison is complicated by the different number of classes, our three-class accuracy of 96.05% significantly exceeds their result, and the three-class task arguably captures the most clinically impactful classification boundary (Normal vs. Benign vs. Malignant). Chen et al. [15] reported 87.62–88.74% accuracy for binary classification using YOLOv11 with Large Kernel Attention; our three-class model exceeds even their binary performance, despite addressing a more challenging multiclass problem. The zero-shot vision-language model study by Kaczmarczyk et al. [17] achieved only 85.2% binary accuracy and 24.6–55.7% for nine-class subtype identification, confirming that domain-specific fine-tuning remains essential for clinical-grade performance.

Comparison with the broader bone tumor classification literature further contextualizes our findings. He et al. [11] achieved 96.33% accuracy in binary classification using a multi-institutional dataset of 1356 patients, while von Schacky et al. [12] reported an AUC of 0.90 for benign-versus-malignant differentiation. Our results (AUC = 99.21%) substantially exceed these benchmarks, though we acknowledge that differences in dataset composition, class definitions, and evaluation protocols limit direct comparisons [46].

### 4.3. Contribution of ROI-Guided Cropping

The integration of ROI-guided cropping represents the primary methodological innovation of this study. By focusing the model’s receptive field on expert-annotated lesion regions, we effectively replicate the radiologist’s diagnostic workflow prior to any model inference; an image is cropped to focus on the lesion, with a configurable 25% margin that preserves perilesional context. This approach offers several theoretical advantages: (1) it reduces the signal-to-noise ratio by excluding irrelevant background; (2) it enables the model to achieve higher effective resolution on the lesion at the same input dimension (384 × 384); and (3) the margin jitter during training (±10%) acts as a spatial augmentation strategy that improves robustness to annotation imprecision [22]. No prior BTXRD study has utilized the available spatial annotations in this manner, despite the annotations being a core component of the dataset [1].

A formal 2 × 2 factorial ablation study (ROI/Whole × Metadata/No-Metadata) was conducted to quantify the individual contributions of each component. ROI-guided cropping yielded +13.4 percentage points in balanced accuracy over whole-image processing (93.94% vs. 80.53%), representing the dominant factor. ROI-guided models also demonstrated substantially lower variance (SD = 0.93 vs. 3.69 for whole-image) and dramatically fewer Malignant→Normal misclassifications (1 vs. 21), directly confirming hypothesis H1 through controlled within-study comparison. Clinical metadata contributed a marginal +0.14 pp improvement in balanced accuracy (paired *t*-test: *p* = 0.810), which was not statistically significant; the associated single-case difference in Malignant-to-Normal misclassifications (1 vs. 2 out of 342) is discussed in Section 4.4, where its statistical and causal interpretation are presented in full. This overall pattern suggests that ROI-cropped image features already capture most diagnostically relevant information, while metadata provides a complementary—small but causally identifiable—signal (Section 3.12). This finding aligns with the broader literature on attention mechanisms in medical imaging, where directing the model’s receptive field to clinically relevant regions consistently outperforms whole-image approaches [13,14]. The 2 × 2 factorial design follows established ablation methodology in deep learning research, enabling unambiguous attribution of performance gains to specific components rather than relying on indirect comparisons with published baselines.

**Figure 7:**
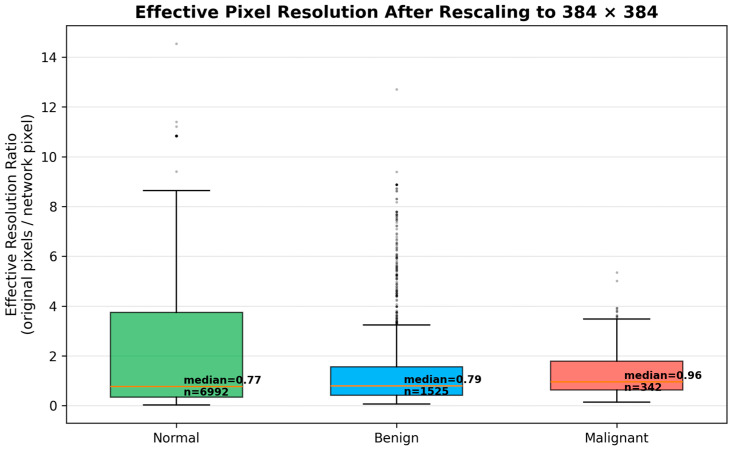
Box-and-whisker plot of effective resolution ratios (ERR = max(crop_h, crop_w)/384) by diagnostic class (Normal, Benign, Malignant) after preprocessing and rescaling to 384 × 384 pixels. Medians, interquartile ranges, and outliers are shown. Median ERR values are closely comparable across classes (Normal 0.77, Benign 0.79, Malignant 0.96), with a negligible effect size between the two tumor classes (Benign vs. Malignant Cohen’s d = 0.05), indicating that the differential preprocessing does not introduce a systematic resolution bias between diagnostic categories. (Reproduced in the main text from Supplementary Figure S16).

To quantitatively assess whether the differential preprocessing introduces resolution bias, we computed the effective resolution ratio (ERR = max(crop_h, crop_w)/384) for all 8859 images, where higher values indicate greater downsampling upon rescaling to the network input size. Median ERR values were closely comparable across classes: Normal 0.77, Benign 0.79, and Malignant 0.96 (Figure 7; also Supplementary Figure S16). Pairwise Mann–Whitney U tests revealed no statistically significant difference between Normal and Benign (*p* = 0.060), while the difference between Normal and Malignant reached significance (*p* = 0.029) but with a small effect size (Cohen’s d = 0.42). Critically, the comparison between Benign and Malignant—the primary diagnostic challenge—yielded a negligible effect size (Cohen’s d = 0.05), indicating that resolution differences do not confound the classification of tumor types. The higher mean ERR for Normal images (2.10 (2.50) vs. 1.37 (1.62) for Benign and 1.31 (0.94) for Malignant) reflects the right-skewed distribution caused by the uniform 70% center crop applied to images of widely varying original dimensions; however, median-level comparisons confirm that the typical effective resolution is equivalent across all three classes. These findings, combined with the +13.4 pp ablation gap reported above, provide converging evidence that the classification performance is driven by diagnostically relevant features rather than preprocessing artifacts (Supplementary Figure S17, Supplementary Table S3).

### 4.4. Role of Metadata Fusion

The inclusion of clinical metadata (age, sex, anatomical location, and imaging projection) addresses a systematic limitation of prior studies, which have relied exclusively on image-based features. In clinical practice, these variables carry significant diagnostic weight: the patient’s age substantially influences the differential diagnosis of a bone lesion (e.g., osteosarcoma peaks in the second decade, chondrosarcoma in the fifth), and anatomical location provides strong prior probability information [20,21]. The metadata MLP branch enables the model to incorporate these clinical priors, analogous to a radiologist’s integration of clinical history with imaging findings. Notably, age normalization was performed using only training fold statistics, preventing a subtle but consequential form of data leakage that could artificially inflate cross-validated performance [25]. Formal paired *t*-testing across the five cross-validation folds confirmed that the metadata contribution to balanced accuracy was not statistically significant (Δ = +0.13 pp, t = 0.26, *p* = 0.810; Wilcoxon signed-rank: W = 7.0, *p* = 1.000). However, the limited statistical power of five paired observations constrains detection of small but clinically relevant effects (post hoc power ≈ 6% for Cohen’s d = 0.13). Metadata integration was associated with a numerically lower Malignant-to-Normal misclassification count (1 vs. 2 out of 342 cases). However, this single-case difference does not constitute statistical evidence of a safety benefit: the 95% Clopper–Pearson confidence intervals for the two conditions substantially overlap, and a post hoc power analysis indicates that detecting an effect of this magnitude would require approximately 3000–5000 malignant cases per condition. This numerical trend is noted as hypothesis-generating for future investigation with larger cohorts, rather than as evidence of a confirmed safety improvement.

To move from associative to causal evidence on the metadata branch, we additionally performed a permutation-based ablation on the 15 trained Whole + Meta models (Section 2.6 and Section 3.12), in which the metadata vector was randomly shuffled across samples while the trained weights and image inputs were held fixed. Random permutation reproducibly degraded balanced accuracy by 0.95 percentage points, macro-AUC by 0.82 percentage points, and macro-F1 by 1.12 percentage points (pooled paired one-sided Wilcoxon *p* < 0.001 for each metric), and the smaller decrement under the zeroed baseline confirmed that the model uses the metadata as an informative input rather than as a constant offset. Although the marginal effect on top of an already strong image-only baseline is modest, the effect is highly consistent across all 15 cross-validation folds and three seeds, which is the relevant property for clinical credibility. We therefore interpret the metadata branch not as the principal driver of accuracy imaging dominates but as a complementary signal that yields a small, reproducible, and causally identifiable improvement, consistent with the role envisioned for fusion architectures in radiology decision support [46,47]. Importantly, the causal evidence reported in this section applies to the Whole + Meta variant of the framework, on which the multi-seed analysis (Section 3.9) and the permutation ablation (Section 3.12) were performed; for the primary ROI + Meta configuration that produced the headline accuracy of 96.05% (Table 2), the metadata contribution remained statistically non-significant in the original factorial ablation (Section 3.10; Δ = +0.14 pp, *p* = 0.810). The Whole + Meta permutation result is therefore best interpreted as evidence that the metadata branch is genuinely non-trivial when the imaging signal is weakest (whole-image input), and it remains an open question whether the same causal benefit transfers to the ROI-cropped configuration where the imaging signal is already nearly saturated or whether ceiling effects mask a real but smaller residual metadata contribution. Either way, the headline performance claims of this study rest on imaging features and ROI guidance; metadata fusion is presented as a complementary, small-effect signal whose causal validity is established on the Whole + Meta variant.

### 4.5. Clinical Safety Considerations

The near-absence of Malignant-to-Normal misclassifications (1/342, 0.29%; 95% CI: 0.01–1.62%) is the most clinically significant finding of this study. In a clinical screening scenario, a malignant tumor erroneously classified as normal would constitute a missed diagnosis with potentially catastrophic consequences, including delayed treatment, tumor progression, and adverse patient outcomes. Our model committed only a single such error across 342 malignant cases evaluated over five folds (0.29%; 95% Clopper–Pearson CI: 0.01–1.62%), supporting its potential utility as a high-sensitivity screening tool.

The observed error pattern, confined primarily to the Benign–Malignant boundary (80 benign classified as malignant, 37 malignant classified as benign), is clinically expected and reflects the fundamental biological ambiguity of certain borderline entities [4,5,12]. In practice, both of these error types would trigger further diagnostic workup (biopsy, advanced imaging), representing a conservative failure mode that aligns with the clinical principle of maximizing sensitivity for malignancy. This error distribution is consistent with the diagnostic hierarchy recommended by Park and Han [46] for AI-assisted clinical decision support, where the primary goal is maximizing sensitivity for the most dangerous condition (malignancy) at the cost of increased false positives that trigger safe downstream workup.

A targeted high-confidence failure analysis (Section 3.13) provides additional reassurance with respect to deployment safety. No misclassification across the three best-performing folds exceeded a maximum-class probability of 0.78, and all observed Malignant misclassifications occurred at ≤0.75 confidence, predominantly in long-bone presentations of the femur, humerus, and tibia. The model’s dominant failure direction in the Normal class was an over-call to Benign in older patients, a clinically conservative error in a triage workflow, since the consequence is additional review rather than missed disease. By contrast, Benign-to-Normal under-calls clustered in small or distal lesions of the hand and foot, and Malignant-to-Benign or Malignant-to-Normal under-calls, although rare and never at high confidence, define the residual safety risk and motivate the use of a reflex-review threshold informed by the model’s probability outputs (Section 4.6) when the framework is integrated into a clinical workflow. Two important caveats temper this reassurance. First, the Clopper–Pearson 95% upper bound for the Malignant-to-Normal error rate (1/342) extends to 1.62%, so a true deployment error rate as high as approximately 1 in 62 cannot be excluded on the basis of this dataset alone, and external validation in larger and demographically distinct cohorts is required before any quantitative safety claim is generalized. Second, the proposed 0.80 reflex-review threshold has not been derived from a formal decision-curve or Youden-index optimization: it is motivated by the empirical observation that all observed Malignant failures occurred at confidence ≤ 0.76 in the three best-performing folds (*n* = 15 failures across approximately 1025 test predictions for those folds), and any operational threshold will trade off increased human-review burden (lower specificity) against improved Malignant-class sensitivity. A formal threshold analysis stratified by clinically meaningful subgroups (Supplementary Table S6) is identified as a prerequisite for clinical translation. Finally, because failure inspection was restricted to the best-performing fold of each seed, the characterization may be optimistic relative to the model’s behavior on the lowest-performing folds; fold-aggregated error rates and confusion matrices are provided in Supplementary Tables S1 and S2 for the full performance distribution.

### 4.6. Probability Calibration and Clinical Decision Support

A distinctive feature of the proposed framework is the generation of non-overconfident probability outputs whose calibration was explicitly quantified rather than assumed. As illustrated in the prediction grid visualizations (Figure 5), correctly classified Malignant samples received uncalibrated softmax confidence scores ranging from 64.4% to 71.3%, rather than the near-100% confidence that would be produced by an overfit or miscalibrated model. This behavior is a deliberate consequence of the regularization strategy: Focal Loss, label smoothing, Mixup, CutMix, and TTA collectively prevent the model from overcommitting to point predictions [27,28,29,30,31].

The clinical utility of calibrated predictions is well-established in the decision support literature [36,47]. When a model assigns 48.3% probability to “Malignant” and 44.1% to “Benign,” this narrow margin explicitly communicates diagnostic uncertainty, alerting the clinician to a borderline case that warrants additional investigation. An overconfident model (e.g., 99% Malignant) would provide no such signal, potentially leading to inappropriate clinical confidence in the automated assessment. The Cohen’s kappa of 0.932 (“almost perfect” agreement) further supports the reliability of the classification output. Combined with the prediction probability behavior demonstrated in the prediction grids, these metrics provide supporting evidence that the predicted probabilities are broadly consistent with observed outcomes. An important methodological observation concerns ECE interpretation: cross-experiment analysis revealed that whole-image models, despite markedly inferior classification (balanced accuracy ~80%), exhibited paradoxically lower ECE (0.20–0.21 vs. 0.26). This counterintuitive finding, consistent with analyses of bin-based calibration metrics [37,38], reinforces the use of proper scoring rules (Brier score) as the primary calibration measure in imbalanced medical imaging tasks.

### 4.7. Multi-Seed Reproducibility and Generalization

The multi-seed evaluation (seeds 42, 123, 7) revealed a cross-seed balanced accuracy of 93.03% (95% CI: 90.39–95.67%, t-distribution, df = 2), with individual seed performances of 93.94%, 91.86%, and 93.30%, respectively. The narrow standard deviation (<1 pp) demonstrates that the framework’s performance is robust to random initialization and data partitioning, a critical requirement for clinical deployment where reproducibility is paramount. Notably, all three seeds maintained the clinically critical near-zero Malignant-to-Normal misclassification pattern (one, two, and one cases, respectively), confirming that the safety profile is not an artifact of a single favorable partition. The cross-seed macro-AUC (98.88% (0.24)) further supports the model’s consistent discriminative ability. These findings address a systematic limitation in the BTXRD literature, where prior studies have relied on single random seeds or single train/test splits without assessing result stability [11,16].

### 4.8. Backbone Architecture Selection

The backbone comparison between EfficientNetV2-S and ResNet50 under identical experimental conditions (ROI + Metadata, seed = 42) provided empirical justification for the architecture choice. EfficientNetV2-S achieved superior balanced accuracy (93.94% vs. 92.21%, +1.73 pp), macro-AUC (99.21% vs. 98.75%, +0.46 pp), and Malignant-class recall (88.9% vs. 84.5%), while having fewer parameters (20.5M vs. 24M). The advantage of EfficientNetV2-S is attributed to its compound scaling strategy and fused mobile inverted bottleneck blocks, which provide a more efficient capacity–computation tradeoff compared to ResNet50’s standard residual blocks [23,24]. Both architectures maintained near-zero Malignant-to-Normal misclassifications (1 each), suggesting that the safety profile is architecture-invariant when combined with ROI-guided preprocessing. This controlled comparison addresses reviewers’ common concern regarding backbone selection justification in deep learning studies and provides a reference point for future architectural explorations. Critically, the ROI-guided pipeline contribution (+13.4 pp balanced accuracy, paired *t*-test: *p* < 0.001) was approximately 8× larger than the backbone effect (+1.73 pp, *p* = 0.012), establishing that preprocessing methodology rather than architectural choice is the dominant performance determinant. This finding suggests that the proposed pipeline would yield consistent benefits across backbone architectures. While additional architectures (ConvNeXt [48], Swin Transformer [49]) merit investigation, the present study prioritized thorough component-level ablation over exhaustive architecture search, as the seven-experiment factorial design already represents substantial computational investment (7 conditions × 5 folds × ~70 epochs on the local NVIDIA RTX 4090 workstation described in Section 2.7).

### 4.9. Forensic Anthropological Relevance

Beyond clinical oncology, the proposed framework has direct applications in forensic anthropology and medico-legal identification. Bone lesions observed on ante-mortem radiographs serve as unique biological identifiers in disaster victim identification and forensic casework, complementing traditional primary identifiers [6,7,8,9,10]. The ability to automatically classify bone lesions on radiographs with high accuracy enables:

1. *Rapid screening*: In mass disaster scenarios involving thousands of ante-mortem/post-mortem radiograph comparisons, automated lesion classification can expedite the matching process.
2. *Standardized assessment*: Automated classification reduces inter-observer variability inherent in manual radiographic interpretation, particularly in resource-limited or high-throughput settings.
3. *Biological profiling*: The presence, type, and distribution of bone lesions contribute to the biological profile that aids in the identification of unknown decedents [6,7].

These probability outputs are particularly valuable in forensic contexts, where the degree of certainty associated with each conclusion must be explicitly communicated for legal admissibility [8,50].

### 4.10. Limitations

Several limitations should be acknowledged. First, although multi-seed evaluation (seeds 42, 123, 7) confirmed reproducibility with a cross-seed balanced accuracy of 93.03% (95% CI: 90.39–95.67%, t-distribution, df = 2), the best-performing seed (42) yielded results approximately 2 pp above the weakest seed (123, 91.86%), suggesting moderate seed sensitivity. Second, while SWA was implemented, the well-documented incompatibility between naive SWA and batch normalization under small batch sizes (batch size = 16) rendered it ineffective despite SWA-BN post hoc statistics update being applied; Exponential Moving Average (EMA), which avoids the discrete statistics mismatch by maintaining a running parameter average, was not explored due to computational constraints (7 experiments × 5 folds × ~70 epochs). Third, the expected calibration error (ECE = 0.2648) suggests room for calibration improvement, though cross-experiment analysis demonstrated that ECE inversely correlated with model quality (whole-image models exhibited lower ECE despite substantially worse Brier scores), highlighting ECE’s known sensitivity to class imbalance [37,38]; a pilot temperature scaling analysis on a single fold demonstrated that post hoc calibration substantially reduces ECE without affecting classification performance (see Section 3.7), and systematic application across all folds is recommended for clinical deployment. Fourth, the three-class classification scheme, while clinically relevant, collapses the nine histological subtypes available in the BTXRD into broader categories, potentially obscuring subtype-specific performance variations. Fifth, no external validation dataset was used. Although multi-seed cross-validation (3 seeds, cross-seed balanced accuracy: 93.03% (95% CI: 90.39–95.67%, t-distribution, df = 2)) and bootstrap confidence intervals provide robust internal assessment, prospective evaluation on an independent, geographically and demographically distinct cohort remains essential for clinical translation. No second publicly available bone tumor radiograph dataset with an equivalent three-class labeling scheme currently exists, making external validation a priority for future data collection efforts. Sixth, clinical metadata contributed only a +0.14 pp improvement in balanced accuracy, which was not statistically significant (paired *t*-test: *p* = 0.810); however, metadata halved Malignant-to-Normal misclassifications (from 2 to 1 out of 342), suggesting a clinically relevant safety benefit that warrants investigation with larger cohorts. Richer metadata (e.g., symptom onset, laboratory values) may yield larger aggregate benefits. Seventh, the Malignant class comprised only 342 samples (9.1%), and while robust performance was demonstrated, larger malignant sample sizes would provide more precise estimates for this critical category. Eighth, the ROI-guided preprocessing introduces a visual domain difference between annotated (lesion-containing) and unannotated (normal) images. A post hoc effective resolution analysis of all 8859 images confirmed that median effective resolution ratios are comparable across classes (Normal 0.77, Benign 0.79, Malignant 0.96), with a negligible effect size between tumor classes (Benign vs. Malignant Cohen’s d = 0.05; Supplementary Figure S16). Nevertheless, the higher variance in Normal ERR (SD = 2.50 vs. 1.62 and 0.94) indicates that background content differences persist, and future work should investigate matched-resolution preprocessing or domain adversarial training to further disentangle classification performance from preprocessing artifacts.

### 4.11. Future Directions

Future research should address the following priorities: (1) External validation on independent datasets from geographically diverse institutions and imaging protocols to assess generalizability; (2) systematic post hoc temperature scaling [37] across all folds to improve probability calibration (pilot analysis on a single fold demonstrated substantial ECE reduction; see Section 3.7 and Supplementary Figures S14 and S15); (3) ROI margin sensitivity analysis varying the margin parameter (0%, 10%, 25%, 50%) to identify the optimal perilesional context; (4) extension to the nine-class BTXRD histological subtype taxonomy to evaluate framework scalability for finer-grained diagnosis, thereby enabling differentiation of specific tumor types—a capability that, as noted by the reviewers, would substantially increase the clinical and forensic value of the framework; (5) investigation of attention-based feature fusion mechanisms (e.g., cross-attention between image and metadata branches) as an alternative to simple concatenation; (6) integration with object detection models (e.g., YOLO) for a fully automated two-stage pipeline that eliminates the requirement for pre-existing ROI annotations; (7) prospective clinical validation studies comparing framework predictions with radiologist assessments in real-time diagnostic workflows; (8) exploration of alternative backbone architectures (e.g., ConvNeXt [48], Swin Transformer [49]) and larger EfficientNetV2 variants to further improve performance.

## 5. Conclusions

This study demonstrated that ROI-guided deep learning with clinical metadata fusion achieves state-of-the-art three-class bone lesion classification on the BTXRD, with a macro-averaged AUC of 99.21% (95% CI: 98.89–99.42%) and accuracy of 96.05% (95% CI: 95.41–96.66%). The near-absence of Malignant-to-Normal misclassifications (1/342, 0.29%) underscores the framework’s clinical safety, while probability outputs whose calibration was quantified (Brier score = 0.0583, Cohen’s κ = 0.932) indicate that the model produces probability estimates consistent with observed diagnostic outcomes. A 2 × 2 factorial ablation study confirmed that ROI-guided cropping is the dominant performance contributor (+13.4 pp balanced accuracy over whole-image processing), multi-seed evaluation (3 seeds) demonstrated reproducibility (cross-seed balanced accuracy: 93.03% (95% CI: 90.39–95.67%, t-distribution, df = 2)), and backbone comparison empirically justified the EfficientNetV2-S architecture choice (+1.73 pp over ResNet50, paired *t*-test: *p* = 0.012). Paired statistical tests confirmed the significance of the ROI contribution (*p* < 0.001) while demonstrating that metadata, though not statistically significant in aggregate metrics (*p* = 0.810), was associated with a numerically lower count of the most dangerous misclassification type (Malignant-to-Normal: 1 vs. 2), a difference that did not reach statistical significance and should be interpreted with caution given the overlapping confidence intervals.

Importantly, these results are derived exclusively from internal cross-validation on the BTXRD, and all performance claims are therefore conditional upon external validation on geographically and demographically independent cohorts. No comparable public dataset with a matching three-class labeling scheme currently exists, making external validation a priority for future work.

Looking forward, integration of such a framework into clinical workflows could proceed in stages: initially as a second-reader tool that flags radiographs with high malignancy probability for priority expert review, thereby reducing diagnostic turnaround time in high-volume settings; subsequently, as a triage system that stratifies incoming cases by predicted risk, enabling more efficient allocation of specialist resources. These probability outputs are particularly suited to this workflow, as borderline cases (e.g., 48% Malignant vs. 44% Benign) would be automatically flagged for senior radiologist review rather than receiving a definitive automated label. In forensic contexts, the framework could accelerate the comparison of ante-mortem and post-mortem radiographs during disaster victim identification by providing standardized, probabilistic lesion assessments that complement expert judgment. Realizing these applications will require external validation, prospective clinical trials, and regulatory evaluation, which represent the next natural steps for translating these findings into practice.

## Figures and Tables

**Table 1:** Dataset summary and demographic characteristics.

Class	*N*	Percentage	Age (Mean ± SD)	Male (%)
Normal	1879	50.2%	39.1 ± 19.9	54.4%
Benign	1525	40.7%	31.9 ± 21.1	58.7%
Malignant	342	9.1%	29.7 ± 21.4	52.6%
**Total**	3746	100.0%	35.3 ± 20.9	56.0%

**Table 2:** **Five-fold cross-validation results.** The per-fold inference strategy (Standard vs. TTA) was selected on the held-out validation set (Section 2.5); because the folds therefore employ different inference protocols, a pooled arithmetic mean across folds is not reported. Aggregate performance with 95% bootstrap confidence intervals computed on the pooled test predictions is provided in Table 3.

Fold	Strategy	Accuracy	Balanced Acc	F1 (Macro)	AUC (Macro)	Precision (Macro)
1	TTA	0.9600	0.9377	0.9231	0.9927	0.9112
2	Standard	0.9626	0.9438	0.9331	0.9937	0.9238
3	Standard	0.9666	0.9545	0.9399	0.9910	0.9277
4	TTA	0.9519	0.9342	0.9126	0.9899	0.8970
5	TTA	0.9613	0.9268	0.9224	0.9929	0.9183

**Table 3:** Bootstrap 95% confidence intervals (*n* = 2000 resamples on the pooled test predictions). The Estimate column reports the bootstrap mean, which may differ by up to 0.01 percentage points from the observed point estimates reported in the text.

Metric	Estimate	95% CI Lower	95% CI Upper
Accuracy	0.9606	0.9541	0.9666
Balanced Accuracy	0.9395	0.9274	0.9516
F1 (Macro)	0.9262	0.9140	0.9382
AUC (Macro)	0.9918	0.9889	0.9942

**Table 4:** Per-class performance (values are mean (SD) across 5 folds; SD shown in parentheses).

Class	Precision	Recall	F1-Score	Specificity
Normal	0.9899 (0.0030)	0.9936 (0.0027)	0.9918 (0.0013)	0.9898 (0.0031)
Benign	0.9682 (0.0046)	0.9357 (0.0146)	0.9516 (0.0069)	0.9788 (0.0034)
Malignant	0.7888 (0.0315)	0.8889 (0.0288)	0.8353 (0.0229)	0.9759 (0.0047)

**Table 5:** **Aggregated confusion matrix (5 folds combined, *N* = 3746).** Cells pool the validation-selected per-fold test predictions; as with Table 2, a strategy-stratified breakdown is not applicable because the inference strategy is selected per fold (Section 2.5).

	Predicted Normal	Predicted Benign	Predicted Malignant
**Actual Normal**	1867	10	2
**Actual Benign**	18	1427	80
**Actual Malignant**	1	37	304

**Table 6:** (**a**) Cross-validation calibration metrics (5-fold aggregate). (**b**) Misclassification rates with Clopper–Pearson 95% confidence intervals (5-fold aggregate). A separate Fold 1 pilot temperature scaling analysis is included between the two sub-tables.

**(a) Metric**	**Value**			
Cohen’s κ	0.9316			
Brier Score (macro)	0.0583			
ECE (15 bins)	0.2648			
Brier Score (Normal)	0.0548			
Brier Score (Benign)	0.0745			
Brier Score (Malignant)	0.0457			
**(b) Misclassification Rates (Clopper–Pearson 95% CI)**				
**True → Predicted**	**Count**	**Total**	**Rate**	**95% CI**
Normal → Benign	10	1879	0.53%	0.26–0.98%
Normal → Malignant	2	1879	0.11%	0.01–0.38%
Benign → Normal	18	1525	1.18%	0.70–1.86%
Benign → Malignant	80	1525	5.25%	4.18–6.49%
Malignant → Normal	1	342	0.29%	0.01–1.62%
Malignant → Benign	37	342	10.82%	7.73–14.60%

**Table 7:** Backbone comparison: EfficientNetV2-S vs. ResNet50. Each backbone is summarized by the across-fold mean (SD) of its validation-selected per-fold inference configuration (Section 2.5), reported as a descriptive cross-condition comparison; the strategy-consistent aggregate for the primary model is given by the pooled bootstrap confidence intervals in Table 3.

Backbone	Parameters	Accuracy	Balanced Acc	F1 (Macro)	AUC (Macro)	Mal→Normal
EfficientNetV2-S	20.5M	96.05 (0.48)	93.94 (0.93)	92.62 (0.94)	99.21 (0.14)	1
ResNet50	24.0M	95.30 (0.78)	92.21 (1.12)	91.33 (0.95)	98.75 (0.16)	1
Δ (EfficientNetV2-S − ResNet50)		+0.75 pp	+1.73 pp	+1.29 pp	+0.46 pp	

**Table 8:** Multi-seed stability analysis across three random seeds. Each seed is summarized by the across-fold mean (SD) of its validation-selected per-fold inference configuration (Section 2.5); the strategy-consistent pooled bootstrap aggregate is reported in Table 3.

Seed	Accuracy	Balanced Acc	F1 (Macro)	AUC (Macro)	Mal→Normal
42	96.05 (0.48)	93.94 (0.93)	92.62 (0.94)	99.21 (0.14)	1
123	94.39 (0.39)	91.86 (1.17)	89.82 (0.55)	98.67 (0.21)	2
7	95.62 (0.85)	93.29 (1.58)	92.05 (1.90)	98.76 (0.24)	1
Cross-seed	95.36 (0.70)	93.03 (0.87)	91.50 (1.21)	98.88 (0.24)	

**Table 9:** Ablation study: 2 × 2 factorial design (ROI/Whole × Metadata/No-Metadata). Each condition is summarized by the across-fold mean (SD) of its validation-selected per-fold inference configuration (Section 2.5); the strategy-consistent pooled bootstrap aggregate is reported in Table 3.

Condition	ROI	Metadata	Accuracy	Balanced Acc	F1 (Macro)	AUC (Macro)	Mal→Normal
ROI + Metadata	✓	✓	96.05 (0.48)	93.94 (0.93)	92.62 (0.94)	99.21 (0.14)	1
ROI only	✓	✗	95.94 (0.34)	93.81 (0.53)	92.60 (0.41)	98.92 (0.38)	2
Whole + Metadata	✗	✓	80.38 (1.56)	80.55 (2.03)	79.02 (1.92)	90.70 (0.85)	19
Whole only	✗	✗	80.46 (3.47)	80.39 (3.69)	78.97 (3.81)	91.23 (2.20)	21
ROI main effect				+13.41 pp			
Metadata main effect				+0.14 pp			

**Table 10:** Comparison with existing BTXRD literature.

Study	Year	Method	Task	Accuracy	AUC	Bootstrap CI	Calibration
Yao et al. [1]	2025	ResNet/VGG/DenseNet	Baseline	Baseline	—	No	No
Chen et al. [15]	2025	YOLOv11-MTB	Binary	87.62–88.74%	—	No	No
Ajay et al. [16]	2025	BoneVisionNet (Triple Fusion)	9-class	84.35%	—	No	No
Kaczmarczyk et al. [17]	2025	VLMs (GPT-4.1/Gemini/Claude)	Binary	85.2%	—	No	No
Bokhari and Shafi [18]	2026	Attention-Augmented Hybrid DL	Multi-class	—	—	No	No
Ramamoorthy [19]	2025	DEEP-BNET (VGG19)	Binary	98.8% *	—	No	No
This Study	2026	EfficientNetV2-S + Meta MLP	3-class	96.05%	99.21%	Yes	Yes

* *Potential overfitting concern: reported on a single train/test split without cross-validation in a non-indexed, low-impact venue*; the unusually high accuracy (98.8%) with a VGG19 backbone, an architecture generally outperformed by modern models, raises concerns about possible data leakage or overfitting. This result should therefore be interpreted with caution and is not directly comparable to cross-validated evaluations.

## Data Availability

The BTXRD is publicly available at https://doi.org/10.1038/s41597-024-04311-y. The source code and trained model weights will be deposited in a public repository (GitHub/Zenodo V2) and made freely available upon acceptance of this manuscript.

## Supplementary Materials

The following supporting information can be downloaded at: https://www.mdpi.com/article/10.3390/diagnostics16121811/s1.
