# Supplementary material for: A Calibrated Deep Learning Framework Integrating Spatial Annotations and Clinical Metadata for Safe Three-Class Bone Lesion Classification on Radiographs
Source: Diagnostics (Basel). 2026 Jun 11;16(12):1811. doi: 10.3390/diagnostics16121811 (PMC13297686; doi:10.3390/diagnostics16121811)
Supplement: Supplementary file 1 [file diagnostics-16-01811-s001.zip › Table_S1.pdf]

Table S1. Hyperparameter configuration for all training experiments.

**Supplementary Table S1. Paired Statistical Tests for Ablation and Backbone Comparisons**	**Unnamed: 1**	**Unnamed: 2**	**Unnamed: 3**	**Unnamed: 4**	**Unnamed: 5**	**Unnamed: 6**	**Unnamed: 7**	**Unnamed: 8**	**Unnamed: 9**	**Unnamed: 10**	**Unnamed: 11**	**Unnamed: 12**	**Unnamed: 13**
Comparison 1: Metadata Effect (ROI+Meta vs. ROI_noMeta)													
Backbone: EfficientNetV2-S, Seed: 42, Input: ROI-guided													
Metric	Fold 1 (A)	Fold 2 (A)	Fold 3 (A)	Fold 4 (A)	Fold 5 (A)	ROI+Meta Mean±SD	ROI_noMeta Mean±SD	Mean Δ	SD(Δ)	t-stat	p (t-test)	W-stat	p (Wilcoxon)
Balanced Accuracy	0.9377	0.9438	0.9545	0.9342	0.9268	0.9394±0.0104	0.9381±0.0060	+0.0013	0.0110	0.257	0.8096 ns	7.0	1.0000 ns
Accuracy	0.9600	0.9626	0.9666	0.9519	0.9613	0.9605±0.0054	0.9594±0.0039	+0.0011	0.0029	0.826	0.4552 ns	3.0	0.2500 ns
F1 (macro)	0.9231	0.9331	0.9399	0.9126	0.9224	0.9262±0.0106	0.9260±0.0045	+0.0003	0.0062	0.092	0.9309 ns	7.0	1.0000 ns
AUC (macro)	0.9927	0.9937	0.9910	0.9899	0.9929	0.9921±0.0016	0.9892±0.0042	+0.0029	0.0045	1.431	0.2257 ns	3.0	0.3125 ns
Δ Balanced Accuracy	+0.0090	+0.0024	+0.0145	-0.0099	-0.0097								
Δ Accuracy	-0.0027	+0.0027	+0.0040	-0.0013	+0.0027								
Δ F1 (macro)	-0.0028	+0.0051	+0.0080	-0.0072	-0.0018								
Δ AUC (macro)	+0.0102	+0.0008	-0.0017	+0.0016	+0.0035								
Comparison 2: ROI Effect													

(ROI+Meta vs. Whole+Meta)													
Backbone: EfficientNetV2-S, Seed: 42, Metadata: Yes													
Metric	Fold 1 (A)	Fold 2 (A)	Fold 3 (A)	Fold 4 (A)	Fold 5 (A)	ROI+Meta Mean±SD	Whole+Meta Mean±SD	Mean Δ	SD(Δ)	t-stat	p (t-test)	W-stat	p (Wilcoxon)
Balanced Accuracy	0.9377	0.9438	0.9545	0.9342	0.9268	0.9394±0.0104	0.8055±0.0227	+0.1339	0.0274	10.921	0.0004 *	0.0	0.0625 ns
Accuracy	0.9600	0.9626	0.9666	0.9519	0.9613	0.9605±0.0054	0.8038±0.0174	+0.1567	0.0212	16.549	<0.0001 *	0.0	0.0625 ns
F1 (macro)	0.9231	0.9331	0.9399	0.9126	0.9224	0.9262±0.0106	0.7902±0.0215	+0.1360	0.0288	10.560	0.0005 *	0.0	0.0625 ns
AUC (macro)	0.9927	0.9937	0.9910	0.9899	0.9929	0.9921±0.0016	0.9070±0.0095	+0.0851	0.0107	17.813	<0.0001 *	0.0	0.0625 ns
Δ Balanced Accuracy	+0.1388	+0.1690	+0.1352	+0.1345	+0.0921								
Δ Accuracy	+0.1533	+0.1829	+0.1736	+0.1335	+0.1402								
Δ F1 (macro)	+0.1319	+0.1573	+0.1705	+0.1228	+0.0975								
Δ AUC (macro)	+0.0849	+0.1008	+0.0864	+0.0711	+0.0820								
Comparison 3: Backbone Effect (EfficientNetV2-S vs. ResNet50)													
Input: ROI+Meta, Seed: 42													
Metric	Fold 1 (A)	Fold 2 (A)	Fold 3 (A)	Fold 4 (A)	Fold 5 (A)	EfficientNetV2-S Mean±SD	ResNet50 Mean±SD	Mean Δ	SD(Δ)	t-stat	p (t-test)	W-stat	p (Wilcoxon)
Balanced Accuracy	0.9377	0.9438	0.9545	0.9342	0.9268	0.9394±0.0104	0.9221±0.0126	+0.0173	0.0089	4.366	0.0120 *	0.0	0.0625 ns
Accuracy	0.9600	0.9626	0.9666	0.9519	0.9613	0.9605±0.0054	0.9530±0.0087	+0.0075	0.0090	1.861	0.1363 ns	1.0	0.1250 ns
F1 (macro)	0.9231	0.9331	0.9399	0.9126	0.9224	0.9262±0.0106	0.9133±0.0106	+0.0129	0.0150	1.921	0.1271 ns	1.0	0.1250 ns
AUC (macro)	0.9927	0.9937	0.9910	0.9899	0.9929	0.9921±0.0016	0.9875±0.0018	+0.0046	0.0012	8.620	0.0010 *	0.0	0.0625 ns
Δ Balanced Accuracy	+0.0099	+0.0318	+0.0128	+0.0194	+0.0125								
Δ Accuracy	-0.001	+0.0214	+0.0107	+0.0053	+0.0013								

	3												
Δ F1 (macro)	-0.0017	+0.0335	+0.0233	+0.0073	+0.0020								
Δ AUC (macro)	+0.0027	+0.0054	+0.0052	+0.0040	+0.0055								
Note: All comparisons use paired samples across 5-fold stratified cross-validation. * indicates $p < 0.05$. ns = not significant. Wilcoxon signed-rank test is provided as a non-parametric alternative; with $n = 5$ paired observations, its power is inherently limited (minimum achievable $p = 0.0625$). Fold-level values for Condition A are shown; Condition B mean±SD is provided in the summary column. Δ rows													

show per-fold differences (A – B).													
