# Supplementary material for: A Calibrated Deep Learning Framework Integrating Spatial Annotations and Clinical Metadata for Safe Three-Class Bone Lesion Classification on Radiographs
Source: Diagnostics (Basel). 2026 Jun 11;16(12):1811. doi: 10.3390/diagnostics16121811 (PMC13297686; doi:10.3390/diagnostics16121811)
Supplement: Supplementary file 1 [file diagnostics-16-01811-s001.zip › Table_S2_TTA_vs_Standard.pdf]

**Table S2. Per-fold comparison of Standard and Test-Time Augmentation (TTA) inference for the ROI-only ablation condition (EfficientNetV2-S, seed = 42), the configuration in which both inference strategies were systematically evaluated on the test set.**

All values are computed on the held-out test fold. The final inference strategy was selected exclusively on the internal validation set (Section 2.5); accordingly, the strategy with the higher test balanced accuracy was not always the one selected (e.g., Fold 1), confirming that strategy selection did not optimise on the test set. The absolute between-strategy difference in test balanced accuracy ranges from 0.09 to 1.07 percentage points, indicating that the two strategies yield comparable performance.

| Fold | Standard Acc | Standard Bal. Acc | TTA Acc | TTA Bal. Acc | \|Δ Bal. Acc\| (pp) | Selected (on val.) |
|---|---|---|---|---|---|---|
| 1 | 0.9627 | 0.9287 | 0.9667 | 0.9357 | 0.70 | Standard |
| 2 | 0.9493 | 0.9406 | 0.9599 | 0.9415 | 0.09 | TTA |
| 3 | 0.9626 | 0.9400 | 0.9586 | 0.9293 | 1.07 | Standard |
| 4 | 0.9453 | 0.9374 | 0.9533 | 0.9441 | 0.67 | TTA |
| 5 | 0.9586 | 0.9365 | 0.9613 | 0.9346 | 0.19 | Standard |
| **Mean (SD)** | **0.9557 (0.0071)** | **0.9366 (0.0043)** | **0.9600 (0.0043)** | **0.9370 (0.0052)** | **—** | **3 Std / 2 TTA** |
