# Supplementary material for: A Calibrated Deep Learning Framework Integrating Spatial Annotations and Clinical Metadata for Safe Three-Class Bone Lesion Classification on Radiographs
Source: Diagnostics (Basel). 2026 Jun 11;16(12):1811. doi: 10.3390/diagnostics16121811 (PMC13297686; doi:10.3390/diagnostics16121811)
Supplement: Supplementary file 1 [file diagnostics-16-01811-s001.zip › Table_S3_Resolution_Statistics.pdf]

Table S3. Pairwise statistical comparison of effective resolution ratios (ERR = max(crop_h, crop_w) / 384) between diagnostic classes. Comparisons use the Kolmogorov–Smirnov (KS) test, the Mann–Whitney U (MW) test, and Cohen's d effect size. Sample sizes are n_1 (first class) and n_2 (second class): Normal = 6992, Benign = 1525, Malignant = 342 annotated ROI regions. Effect-size interpretation (Cohen's d): < 0.2 negligible; 0.2–0.5 small; 0.5–0.8 medium; > 0.8 large.

Comparison	KS statistic	KS p-value	MW U statistic	MW p-value	Cohen's d
Normal vs Benign	0.194	1.42×10^{-41}	5495200.5	0.060	0.35
Normal vs Malignant	0.260	6.03×10^{-20}	1112252	0.029	0.42
Benign vs Malignant	0.192	1.71×10^{-9}	219344.5	4.27×10^{-6}	0.05
