# Supplementary material for: A Calibrated Deep Learning Framework Integrating Spatial Annotations and Clinical Metadata for Safe Three-Class Bone Lesion Classification on Radiographs
Source: Diagnostics (Basel). 2026 Jun 11;16(12):1811. doi: 10.3390/diagnostics16121811 (PMC13297686; doi:10.3390/diagnostics16121811)
Supplement: Supplementary file 1 [file diagnostics-16-01811-s001.zip › Table_S4_Permutation_PerFold.pdf]

Table S4 (per_fold). Permutation-based causal ablation — per-fold metrics (real / permuted / zeroed; 3 seeds × 5 folds × 5 permutations).

seed	fold	mode	permutation_index	accuracy	balanced_accuracy	macro_F1	macro_AUC
7	1	permuted	0	0.7533	0.7638	0.7443	0.8886
7	1	permuted	1	0.76	0.772	0.7513	0.8902
7	1	permuted	2	0.7653	0.7745	0.7586	0.8912
7	1	permuted	3	0.756	0.7654	0.7487	0.8898
7	1	permuted	4	0.7573	0.7652	0.7468	0.8919
7	1	real	-1	0.7987	0.7946	0.7821	0.905
7	1	zeroed	-1	0.7613	0.7689	0.7525	0.8944
7	2	permuted	0	0.8425	0.8227	0.8262	0.9372
7	2	permuted	1	0.8478	0.8266	0.8317	0.9362
7	2	permuted	2	0.8451	0.8288	0.8322	0.9357
7	2	permuted	3	0.8491	0.8234	0.8316	0.9336
7	2	permuted	4	0.8398	0.8171	0.8219	0.9339
7	2	real	-1	0.8505	0.8282	0.835	0.9383
7	2	zeroed	-1	0.8491	0.8275	0.8326	0.9357
7	3	permuted	0	0.7837	0.7926	0.7626	0.9059
7	3	permuted	1	0.7944	0.8003	0.7694	0.9028
7	3	permuted	2	0.7944	0.805	0.7757	0.9057
7	3	permuted	3	0.7877	0.7997	0.7703	0.9032
7	3	permuted	4	0.8011	0.8056	0.7771	0.9033
7	3	real	-1	0.8037	0.8074	0.7801	0.913
7	3	zeroed	-1	0.7944	0.797	0.7641	0.9103
7	4	permuted	0	0.8011	0.8078	0.7698	0.895
7	4	permuted	1	0.8024	0.8094	0.7731	0.8985
7	4	permuted	2	0.7877	0.7971	0.7581	0.8974
7	4	permuted	3	0.7957	0.8047	0.7682	0.9012
7	4	permuted	4	0.8024	0.8089	0.7718	0.8985
7	4	real	-1	0.8104	0.8174	0.7803	0.9038
7	4	zeroed	-1	0.7971	0.8042	0.7663	0.9003
7	5	permuted	0	0.8077	0.8269	0.8071	0.9178
7	5	permuted	1	0.7957	0.8187	0.7985	0.917
7	5	permuted	2	0.7971	0.8156	0.7972	0.9139
7	5	permuted	3	0.8011	0.8184	0.8032	0.9168
7	5	permuted	4	0.8011	0.8227	0.8039	0.9165
7	5	real	-1	0.8091	0.828	0.8096	0.9239
7	5	zeroed	-1	0.8144	0.8343	0.8133	0.9187
42	1	permuted	0	0.776	0.7829	0.7574	0.8981
42	1	permuted	1	0.764	0.7739	0.7471	0.8919
42	1	permuted	2	0.7693	0.778	0.7501	0.8912
42	1	permuted	3	0.7693	0.7774	0.7497	0.8903
42	1	permuted	4	0.7707	0.7795	0.7503	0.8956
42	1	real	-1	0.7773	0.7825	0.752	0.906
42	1	zeroed	-1	0.7747	0.7832	0.7517	0.8985
42	2	permuted	0	0.8291	0.8437	0.8239	0.9327
42	2	permuted	1	0.8371	0.8497	0.8313	0.9347
42	2	permuted	2	0.8304	0.8444	0.8261	0.9363
42	2	permuted	3	0.8304	0.8442	0.8246	0.9371
42	2	permuted	4	0.8318	0.8451	0.8269	0.937
42	2	real	-1	0.8344	0.8469	0.8289	0.9392
42	2	zeroed	-1	0.8318	0.8453	0.8242	0.9376
42	3	permuted	0	0.7757	0.7994	0.7631	0.9102
42	3	permuted	1	0.7704	0.8111	0.7646	0.906
42	3	permuted	2	0.7784	0.8103	0.7696	0.9077
42	3	permuted	3	0.7824	0.8163	0.7687	0.9073
42	3	permuted	4	0.7757	0.8117	0.7716	0.9083

42	3	real	-1	0.7984	0.824	0.7864	0.9197
42	3	zeroed	-1	0.781	0.8188	0.7672	0.911
42	4	permuted	0	0.7917	0.7748	0.776	0.9
42	4	permuted	1	0.7904	0.7739	0.7736	0.8994
42	4	permuted	2	0.7931	0.7759	0.7771	0.9
42	4	permuted	3	0.7984	0.7796	0.7768	0.8995
42	4	permuted	4	0.7997	0.7799	0.7786	0.8991
42	4	real	-1	0.8144	0.7951	0.7966	0.9067
42	4	zeroed	-1	0.7957	0.7804	0.7765	0.9025
42	5	permuted	0	0.7931	0.7886	0.7881	0.9018
42	5	permuted	1	0.8104	0.805	0.8032	0.9046
42	5	permuted	2	0.8024	0.7944	0.7931	0.907
42	5	permuted	3	0.8037	0.7961	0.7944	0.9027
42	5	permuted	4	0.7957	0.7904	0.7885	0.9024
42	5	real	-1	0.8064	0.7973	0.7975	0.9125
42	5	zeroed	-1	0.8024	0.8013	0.7941	0.9063
123	1	permuted	0	0.8213	0.8198	0.8083	0.9137
123	1	permuted	1	0.816	0.8123	0.8009	0.9137
123	1	permuted	2	0.8147	0.8119	0.8001	0.9149
123	1	permuted	3	0.8173	0.8172	0.8054	0.9145
123	1	permuted	4	0.8133	0.8106	0.8004	0.9142
123	1	real	-1	0.8227	0.8205	0.8119	0.9228
123	1	zeroed	-1	0.8293	0.8258	0.8115	0.918
123	2	permuted	0	0.7917	0.7815	0.7917	0.8775
123	2	permuted	1	0.7917	0.7847	0.7937	0.876
123	2	permuted	2	0.8024	0.793	0.8034	0.8775
123	2	permuted	3	0.8077	0.797	0.8057	0.8807
123	2	permuted	4	0.7997	0.791	0.8031	0.8814
123	2	real	-1	0.8131	0.8003	0.8144	0.8831
123	2	zeroed	-1	0.8024	0.7914	0.8007	0.8805
123	3	permuted	0	0.8158	0.7947	0.7955	0.9089
123	3	permuted	1	0.8198	0.7974	0.7984	0.909
123	3	permuted	2	0.8117	0.7953	0.7933	0.9093
123	3	permuted	3	0.8158	0.7986	0.7964	0.909
123	3	permuted	4	0.8171	0.7992	0.7958	0.9111
123	3	real	-1	0.8224	0.8032	0.7998	0.9163
123	3	zeroed	-1	0.8198	0.8031	0.8001	0.9102
123	4	permuted	0	0.8211	0.8065	0.7961	0.9178
123	4	permuted	1	0.8211	0.8035	0.7942	0.9151
123	4	permuted	2	0.8158	0.8034	0.7925	0.9173
123	4	permuted	3	0.8198	0.8099	0.795	0.9162
123	4	permuted	4	0.8171	0.8041	0.7908	0.9195
123	4	real	-1	0.8264	0.8067	0.7965	0.9211
123	4	zeroed	-1	0.8184	0.8048	0.7917	0.9195
123	5	permuted	0	0.7517	0.7709	0.7348	0.8804
123	5	permuted	1	0.7543	0.7729	0.7368	0.8778
123	5	permuted	2	0.7597	0.776	0.7402	0.8795
123	5	permuted	3	0.7437	0.765	0.7298	0.8785
123	5	permuted	4	0.761	0.7775	0.7438	0.8777
123	5	real	-1	0.7837	0.7934	0.7604	0.8935
123	5	zeroed	-1	0.7557	0.7678	0.7297	0.8844

Table S4 (legend). Permutation-based causal ablation — per-fold metrics (real / permuted / zeroed; 3 seeds × 5 folds × 5 permutations).

Field	Description

seed	Random seed for the StratifiedKFold split (matches training).
fold	Cross-validation fold index (1–5).
mode	Inference condition: real (unmodified metadata), permuted (random shuffle of metadata vectors across samples), zeroed (metadata = 0).
permutation_index	Index of the permutation replicate (0–4 for permuted; -1 for real/zeroed).
accuracy	Top-1 accuracy on the held-out test fold.
balanced_accuracy	Macro-averaged recall (balanced accuracy) on the held-out test fold.
macro_F1	Macro-averaged F1-score on the held-out test fold.
macro_AUC	Macro-averaged one-vs-rest AUC on the held-out test fold.
