# Supplementary material for: A Calibrated Deep Learning Framework Integrating Spatial Annotations and Clinical Metadata for Safe Three-Class Bone Lesion Classification on Radiographs
Source: Diagnostics (Basel). 2026 Jun 11;16(12):1811. doi: 10.3390/diagnostics16121811 (PMC13297686; doi:10.3390/diagnostics16121811)
Supplement: Supplementary file 1 [file diagnostics-16-01811-s001.zip › Table_S5_Permutation_Summary.pdf]

Table S5 (POOLED_summary_v2). Permutation-based causal ablation — pooled summary with Bonferroni- and Holm-adjusted p-values and percentile-bootstrap 95% confidence intervals.

metric	n_folds	real_mean	permuted_mean	zeroed_mean	delta_real_minus_permuted_mean_pp	delta_real_minus_permuted_95CI_lo_pp	delta_real_minus_permuted_95CI_hi_pp	delta_real_minus_zeroed_mean_pp	delta_real_minus_zeroed_95CI_lo_pp	delta_real_minus_zeroed_95CI_hi_pp	wilcoxon_real_vs_permuted_p_raw	wilcoxon_real_vs_zeroed_p_raw	wilcoxon_real_vs_permuted_p_bonferroni	wilcoxon_real_vs_permuted_p_holm	wilcoxon_real_vs_zeroed_p_bonferroni	wilcoxon_real_vs_zeroed_p_holm
acc	15	0.811439999999999999	0.798134666666666667	0.801833333333333335	1.330533333333334	0.8617266666666684	1.886536666666665	0.9606666666666668	0.4033166666666667	1.565333333333332	3.0517578125e-05	0.003793025771387344	0.000244140625	0.000244140625	0.03034420617109875	0.007586051542774687
bal_acc	15	0.8097	0.800173333333333333	0.803586666666666666	0.9526666666666668	0.6127866666666666	1.349476666666666	0.6113333333333318	0.1539999999999993	1.130666666666664	3.0517578125e-05	0.0250058733012445	0.000244140625	0.000244140625	0.200046986409956	0.0250058733012445
f1_macro	15	0.795433333333333333	0.784264000000000001	0.785080000000000002	1.116933333333333	0.7057033333333316	1.572936666666668	1.035333333333332	0.5006499999999967	1.573333333333333	3.0517578125e-05	0.00762939453125	0.000244140625	0.000244140625	0.006103515625	0.002288818359375
auc_macro	15	0.913660000000000001	0.905518666666666667	0.908526666666666665	0.8141333333333356	0.637060000000003	1.008273333333334	0.5133333333333353	0.376000000000003	0.658683333333336	3.0517578125e-05	0.003258330403069556	0.000244140625	0.000244140625	0.002606664322455645	0.001303332161227822

Table S5 (per_seed_and_pooled_legacy). Permutation-based causal ablation — pooled summary with Bonferroni- and Holm-adjusted p-values and percentile-bootstrap 95% confidence intervals.

seed	metric	real_mean	permuted_mean	zeroed_mean	delta_permuted_mean	delta_zeroed_mean	n_folds
42	acc	0.8062	0.7948	0.7971	-0.0114	-0.0091	5
42	bal_acc	0.8092	0.8011	0.8058	-0.0081	-0.0033	5
42	f1_macr	0.7923	0.783	0.7827	-0.0093	-0.0095	5

	o						
42	auc_macro	0.9168	0.908	0.9112	-0.0088	-0.0056	5
7	acc	0.8145	0.7988	0.8033	-0.0157	-0.0112	5
7	bal_acc	0.8151	0.8037	0.8064	-0.0114	-0.0087	5
7	f1_macro	0.7974	0.784	0.7858	-0.0135	-0.0117	5
7	auc_macro	0.9168	0.9089	0.9119	-0.0079	-0.0049	5
123	acc	0.8137	0.8009	0.8051	-0.0128	-0.0085	5
123	bal_acc	0.8048	0.7958	0.7986	-0.0091	-0.0063	5
123	f1_macro	0.7966	0.7858	0.7867	-0.0107	-0.0099	5
123	auc_macro	0.9074	0.8997	0.9025	-0.0077	-0.0048	5
POOLED	acc	0.8114	0.7981	0.8018	-0.0133	-0.0096	15
POOLED	bal_acc	0.8097	0.8002	0.8036	-0.0095	-0.0061	15
POOLED	f1_macro	0.7954	0.7843	0.7851	-0.0112	-0.0104	15
POOLED	auc_macro	0.9137	0.9055	0.9085	-0.0082	-0.0051	15

Table S5 (legend). Permutation-based causal ablation — pooled summary with Bonferroni- and Holm-adjusted p-values and percentile-bootstrap 95% confidence intervals.

Column	Description
metric	Performance metric (acc, bal_acc, f1_macro, auc_macro).
n_folds	Number of paired observations (15 = 3 seeds × 5 folds).
real_mean	Mean of the metric under each input mode across the 15 folds.
permuted_mean	Mean of the metric under permuted metadata (averaged over 5 permutations per fold).
zeroed_mean	Mean of the metric under zeroed metadata.
delta_real_minus_permuted_mean_pp	Mean drop in metric (percentage points) when metadata is randomly permuted.
delta_real_minus_permuted_95CI_{lo,hi}_pp	Percentile bootstrap (5000 resamples, RNG seed 20260425) 95% CI for the mean delta.
delta_real_minus_zeroed_mean_pp	Mean drop when metadata is zeroed; same units.
delta_real_minus_zeroed_95CI_{lo,hi}_pp	Bootstrap 95% CI for the zeroed delta.
wilcoxon_real_vs_permuted_p_raw	Raw paired one-sided Wilcoxon signed-rank p (H1: real > permuted).
wilcoxon_real_vs_permuted_p_bonferroni	Bonferroni-corrected p across the family of 8 pooled tests (4 metrics × 2 contrasts).
wilcoxon_real_vs_permuted_p_holm	Holm step-down corrected p across the same family.
wilcoxon_real_vs_zeroed_p_{raw,bonferroni,holm}	Same three p-value variants for the real-vs-zeroed contrast.
