# Supplementary material for: A Calibrated Deep Learning Framework Integrating Spatial Annotations and Clinical Metadata for Safe Three-Class Bone Lesion Classification on Radiographs
Source: Diagnostics (Basel). 2026 Jun 11;16(12):1811. doi: 10.3390/diagnostics16121811 (PMC13297686; doi:10.3390/diagnostics16121811)
Supplement: Supplementary file 1 [file diagnostics-16-01811-s001.zip › Table_S6_Failure_Subgroup_Summary.pdf]

Table S6 (by_age_band). High-confidence failure subgroup tabulation — counts and rates by age band, sex, and anatomical region.

true_name	pediatric (≤18)	adult (19–50)	older (>50)
Benign	5	4	6
Malignant	6	5	4
Normal	4	4	7

Table S6 (by_sex). High-confidence failure subgroup tabulation — counts and rates by age band, sex, and anatomical region.

true_name	F	M
Benign	11	4
Malignant	7	8
Normal	8	7

Table S6 (by_anatomy_region). High-confidence failure subgroup tabulation — counts and rates by age band, sex, and anatomical region.

true_name	lower limb	pelvis	upper limb
Benign	6	2	7
Malignant	8	0	7
Normal	8	0	7

Table S6 (by_error_kind). High-confidence failure subgroup tabulation — counts and rates by age band, sex, and anatomical region.

true_name	error_kind	n
Benign	Benign→Malignant	4
Benign	Benign→Normal	11
Malignant	Malignant→Benign	9
Malignant	Malignant→Normal	6
Normal	Normal→Benign	15

Table S6 (raw_top15). High-confidence failure subgroup tabulation — counts and rates by age band, sex, and anatomical region.

seed	fold	image_id	true_name	pred_name	conf	age	age_band	gender	region
42	2	IMG001943.jpeg	Normal	Benign	0.7816433	56	older (>50)	F	upper limb
42	2	IMG002479.jpeg	Normal	Benign	0.72686654	27	adult (19–50)	M	upper limb
42	2	IMG003377.jpeg	Normal	Benign	0.7217373	57	older (>50)	F	lower limb
42	2	IMG003291.jpeg	Normal	Benign	0.7034471	27	adult (19–50)	M	upper limb
42	2	IMG003049.jpeg	Normal	Benign	0.69850445	57	older (>50)	F	lower limb
42	2	IMG000730.jpeg	Benign	Normal	0.7085843	65	older (>50)	F	upper limb
42	2	IMG000729.jpe	Benign	Normal	0.6929898	65	older	F	upper

		g					(>50)		limb
42	2	IMG001839.jpeg	Benign	Malignant	0.68992084	6	pediatric (≤18)	F	upper limb
42	2	IMG000545.jpeg	Benign	Normal	0.6749268	62	older (>50)	F	lower limb
42	2	IMG000690.jpeg	Benign	Normal	0.6676474	11	pediatric (≤18)	F	pelvis
42	2	IMG001748.jpeg	Malignant	Benign	0.75460416	23	adult (19–50)	M	upper limb
42	2	IMG001767.jpeg	Malignant	Benign	0.5990035	25	adult (19–50)	M	upper limb
42	2	IMG001865.jpeg	Malignant	Benign	0.5238353	9	pediatric (≤18)	F	lower limb
42	2	IMG000024.jpeg	Malignant	Normal	0.5193353	67	older (>50)	M	lower limb
42	2	IMG000221.jpeg	Malignant	Benign	0.5055116	55	older (>50)	M	lower limb
7	2	IMG003741.jpg	Normal	Benign	0.7349161	55	older (>50)	M	lower limb
7	2	IMG003377.jpeg	Normal	Benign	0.7073924	57	older (>50)	F	lower limb
7	2	IMG003317.jpeg	Normal	Benign	0.7057157	42	adult (19–50)	F	upper limb
7	2	IMG003374.jpeg	Normal	Benign	0.69250375	55	older (>50)	M	upper limb
7	2	IMG003266.jpeg	Normal	Benign	0.68864334	11	pediatric (≤18)	M	lower limb
7	2	IMG000258.jpeg	Benign	Normal	0.7189569	46	adult (19–50)	F	upper limb
7	2	IMG000785.jpeg	Benign	Malignant	0.70456344	17	pediatric (≤18)	M	lower limb
7	2	IMG000812.jpeg	Benign	Malignant	0.6959718	38	adult (19–50)	F	lower limb
7	2	IMG000492.jpeg	Benign	Normal	0.6937768	70	older (>50)	F	lower limb
7	2	IMG000367.jpeg	Benign	Normal	0.69003946	61	older (>50)	M	upper limb
7	2	IMG001430.jpeg	Malignant	Benign	0.7134161	15	pediatric (≤18)	F	upper limb
7	2	IMG001363.jpeg	Malignant	Normal	0.63530636	10	pediatric (≤18)	M	lower limb
7	2	IMG000193.jpeg	Malignant	Benign	0.631727	18	pediatric (≤18)	F	lower limb
7	2	IMG001740.jpeg	Malignant	Benign	0.61313206	22	adult (19–50)	F	lower limb
7	2	IMG000211.jpeg	Malignant	Benign	0.59139335	6	pediatric (≤18)	F	upper limb
123	1	IMG001882.jpeg	Normal	Benign	0.7063361	29	adult (19–50)	F	lower limb
123	1	IMG003629.jpeg	Normal	Benign	0.6901613	4	pediatric (≤18)	M	upper limb
123	1	IMG003745.jpg	Normal	Benign	0.6796074	8	pediatric (≤18)	M	lower limb
123	1	IMG003086.jpeg	Normal	Benign	0.6747682	5	pediatric (≤18)	F	upper limb
123	1	IMG003377.jpeg	Normal	Benign	0.6744844	57	older (>50)	F	lower limb
123	1	IMG000691.jpeg	Benign	Normal	0.77737683	11	pediatric (≤18)	F	lower limb

123	1	IMG000792.jpeg	Benign	Malignant	0.75272554	47	adult (19–50)	M	pelvis
123	1	IMG000235.jpeg	Benign	Normal	0.74616617	65	older (>50)	F	upper limb
123	1	IMG000437.jpeg	Benign	Normal	0.7385413	35	adult (19–50)	M	upper limb
123	1	IMG000703.jpeg	Benign	Normal	0.7377446	15	pediatric (≤18)	F	lower limb
123	1	IMG001767.jpeg	Malignant	Benign	0.69505894	25	adult (19–50)	M	upper limb
123	1	IMG000015.jpeg	Malignant	Normal	0.6210753	64	older (>50)	M	upper limb
123	1	IMG001363.jpeg	Malignant	Normal	0.6088325	10	pediatric (≤18)	M	lower limb
123	1	IMG001492.jpeg	Malignant	Normal	0.6035822	20	adult (19–50)	F	lower limb
123	1	IMG000149.jpeg	Malignant	Normal	0.5889965	70	older (>50)	F	upper limb

Table S6 (legend). High-confidence failure subgroup tabulation — counts and rates by age band, sex, and anatomical region.

Sheet	Description
by_age_band	Counts of top-5 failures per true class, stratified by age band (pediatric ≤18, adult 19–50, older >50). Aggregated across the three best-performing folds (seed 42 fold 2, seed 7 fold 2, seed 123 fold 1) — total n = 45 (15 per fold).
by_sex	Counts of top-5 failures per true class, stratified by patient sex.
by_anatomy_region	Counts of top-5 failures per true class, stratified by primary anatomical region (upper limb, lower limb, pelvis, other).
by_error_kind	Counts of each error kind (true → predicted class) across the top-5-per-class failures.
raw_top15	Raw top-5-per-class failure rows used to build the cross-tabs.
