# Supplementary figures and images for: A Calibrated Deep Learning Framework Integrating Spatial Annotations and Clinical Metadata for Safe Three-Class Bone Lesion Classification on Radiographs
Source: Diagnostics (Basel). 2026 Jun 11;16(12):1811. doi: 10.3390/diagnostics16121811 (PMC13297686; doi:10.3390/diagnostics16121811)

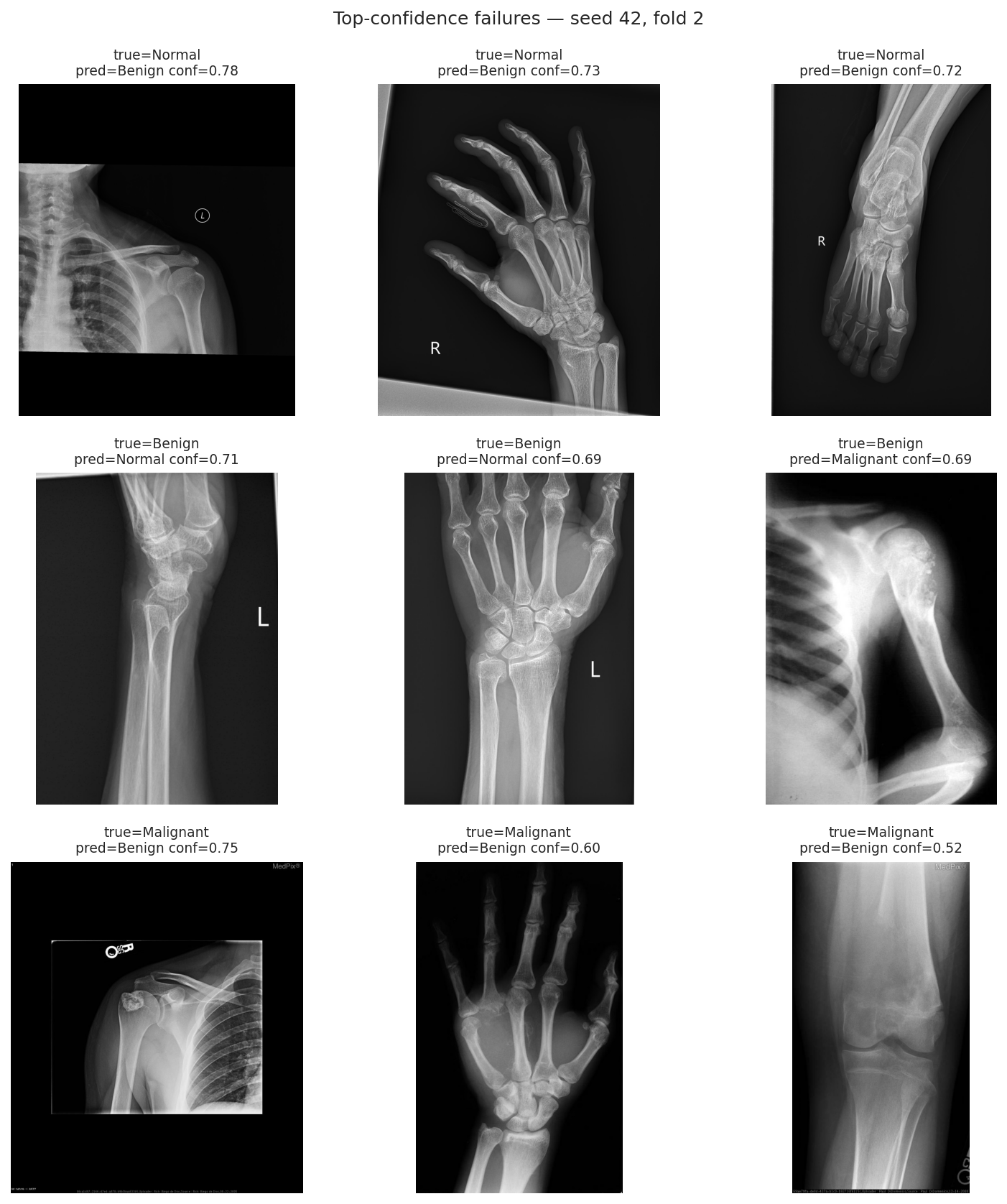

Supplement: Supplementary file 1 [file diagnostics-16-01811-s001.zip › Figure_S18_failure_grid_seed42_fold2.png]

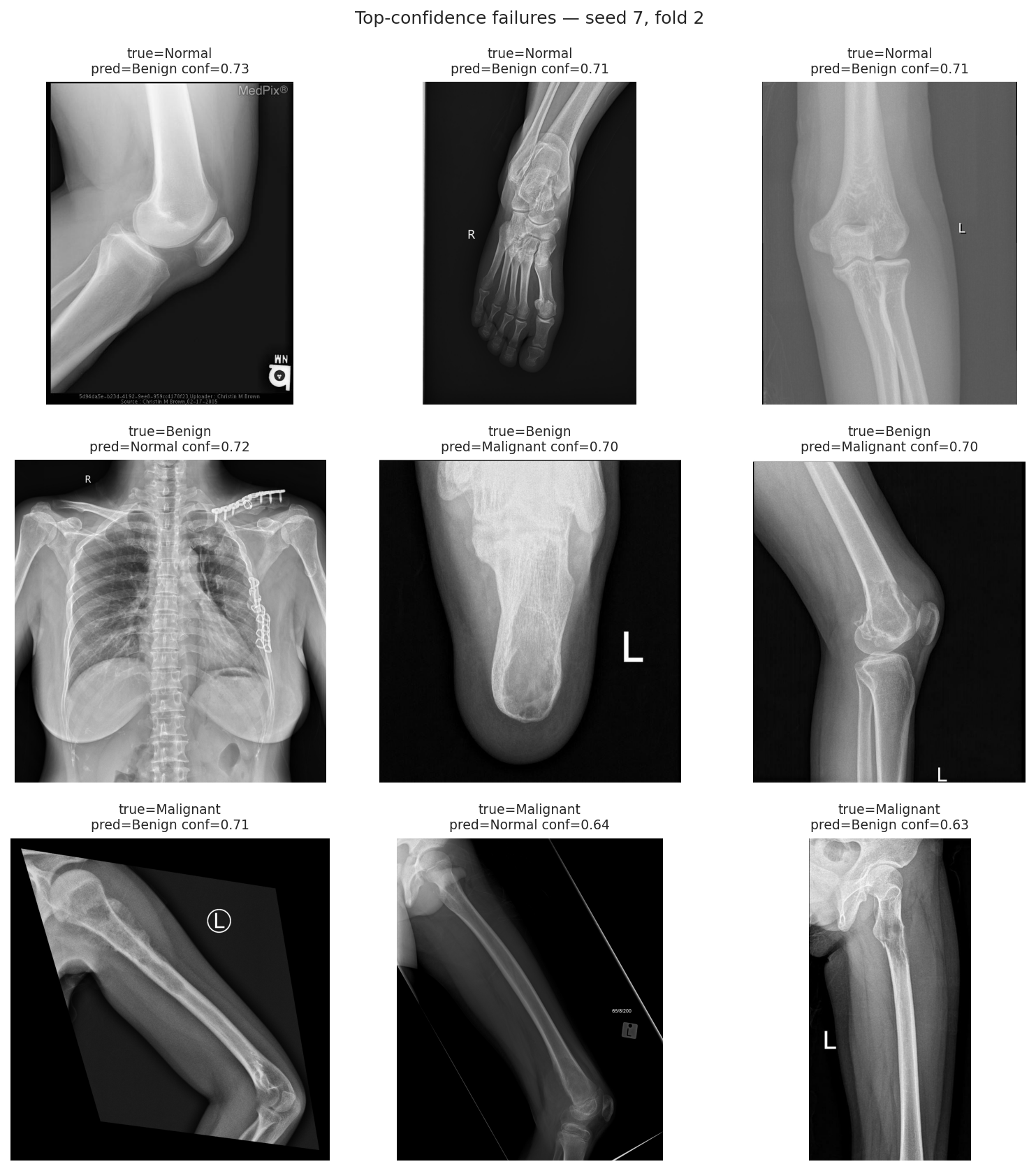

Supplement: Supplementary file 1 [file diagnostics-16-01811-s001.zip › Figure_S19_failure_grid_seed7_fold2.png]

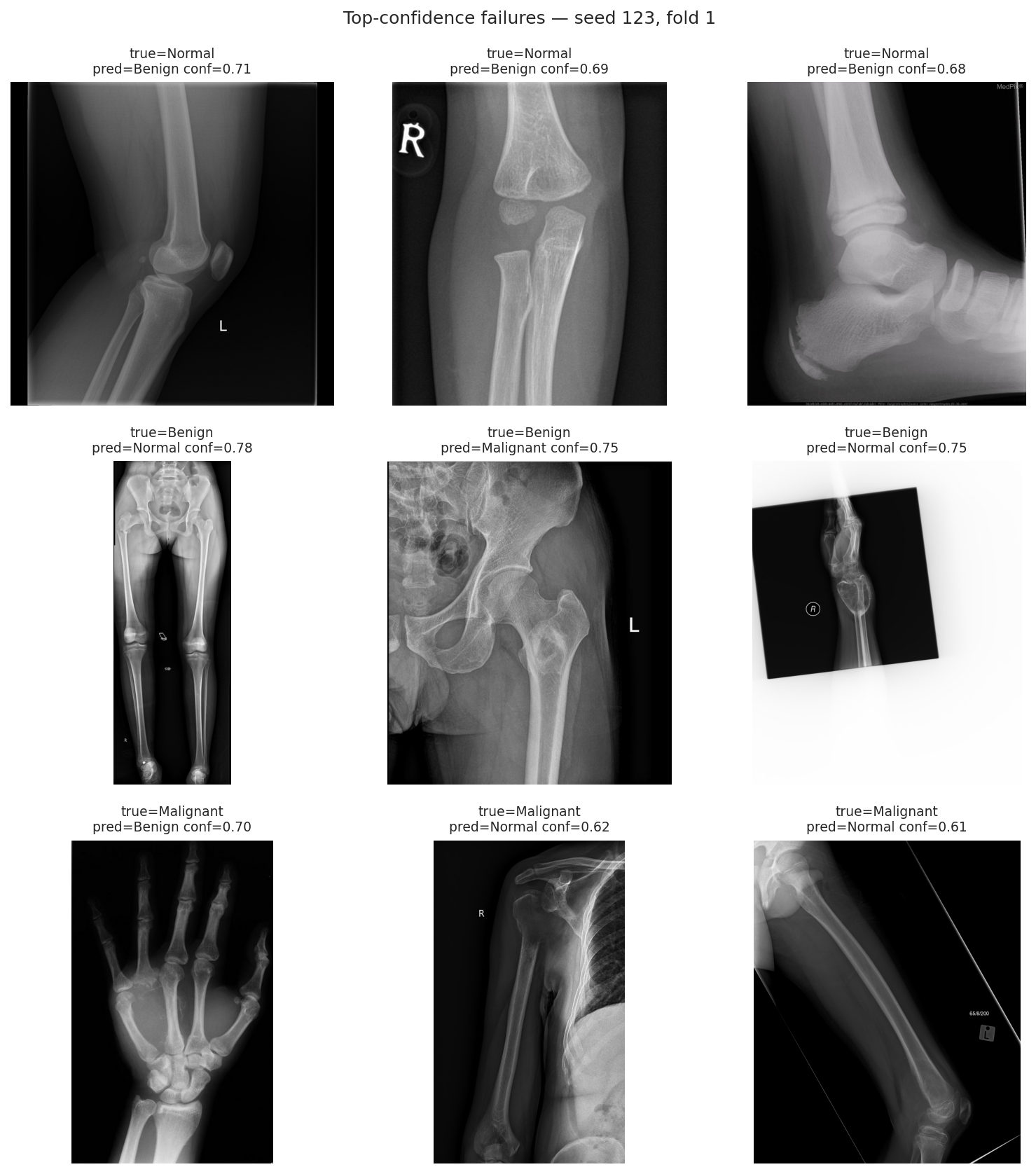

Supplement: Supplementary file 1 [file diagnostics-16-01811-s001.zip › Figure_S20_failure_grid_seed123_fold1.png]

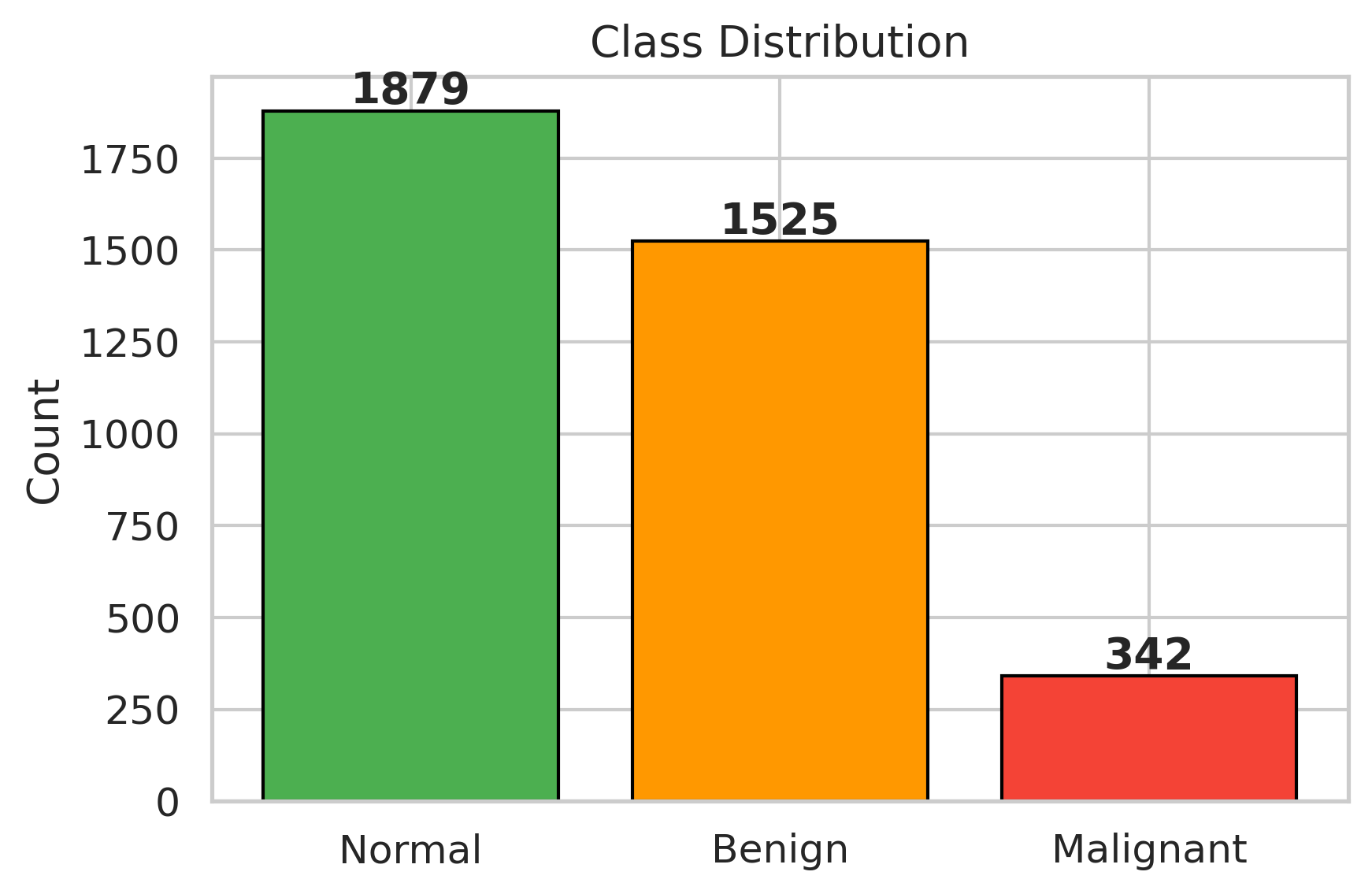

Supplement: Supplementary file 1 [file diagnostics-16-01811-s001.zip › Figure_S1.png]

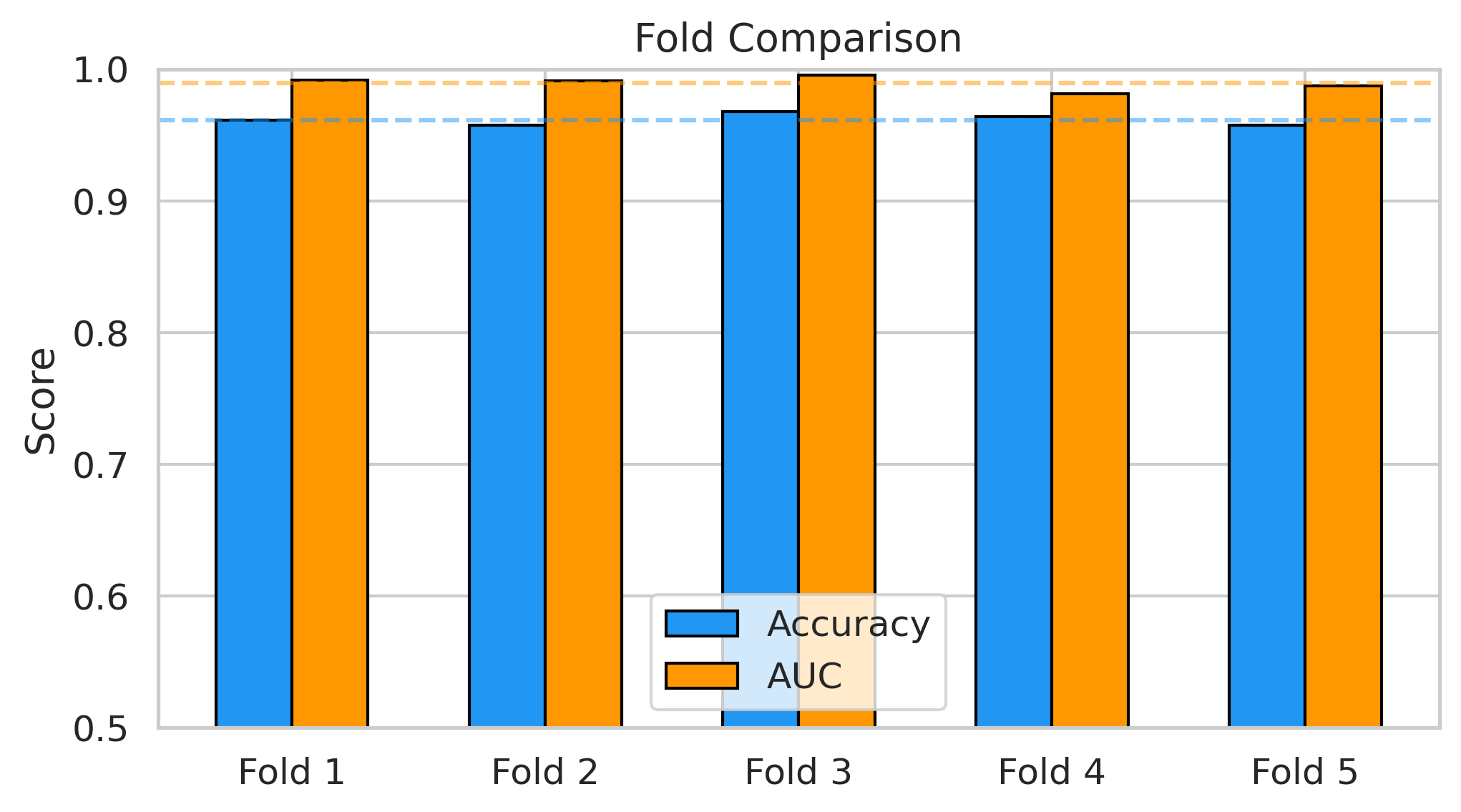

Supplement: Supplementary file 1 [file diagnostics-16-01811-s001.zip › Figure_S2.png]

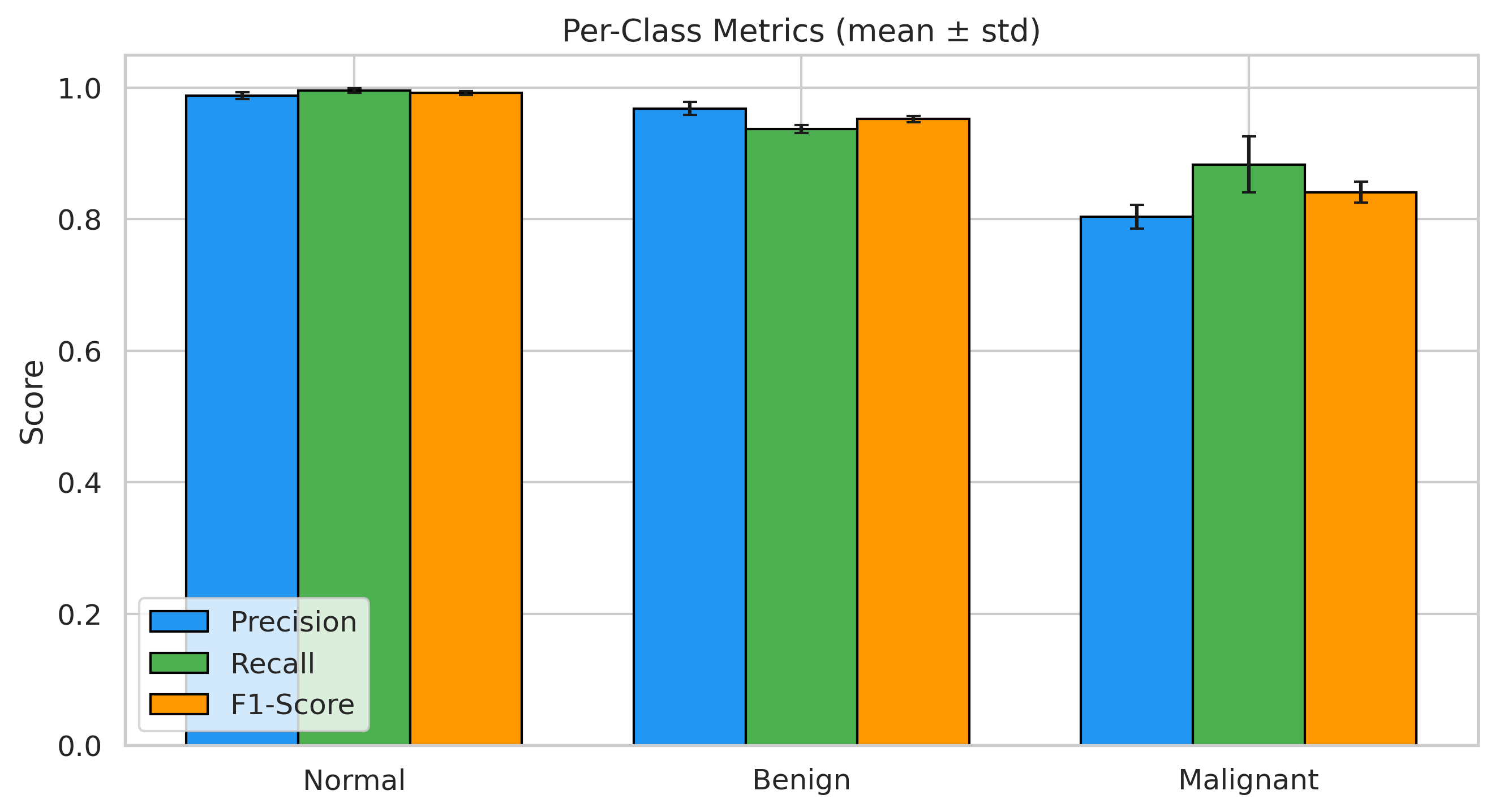

Supplement: Supplementary file 1 [file diagnostics-16-01811-s001.zip › Figure_S3.png]

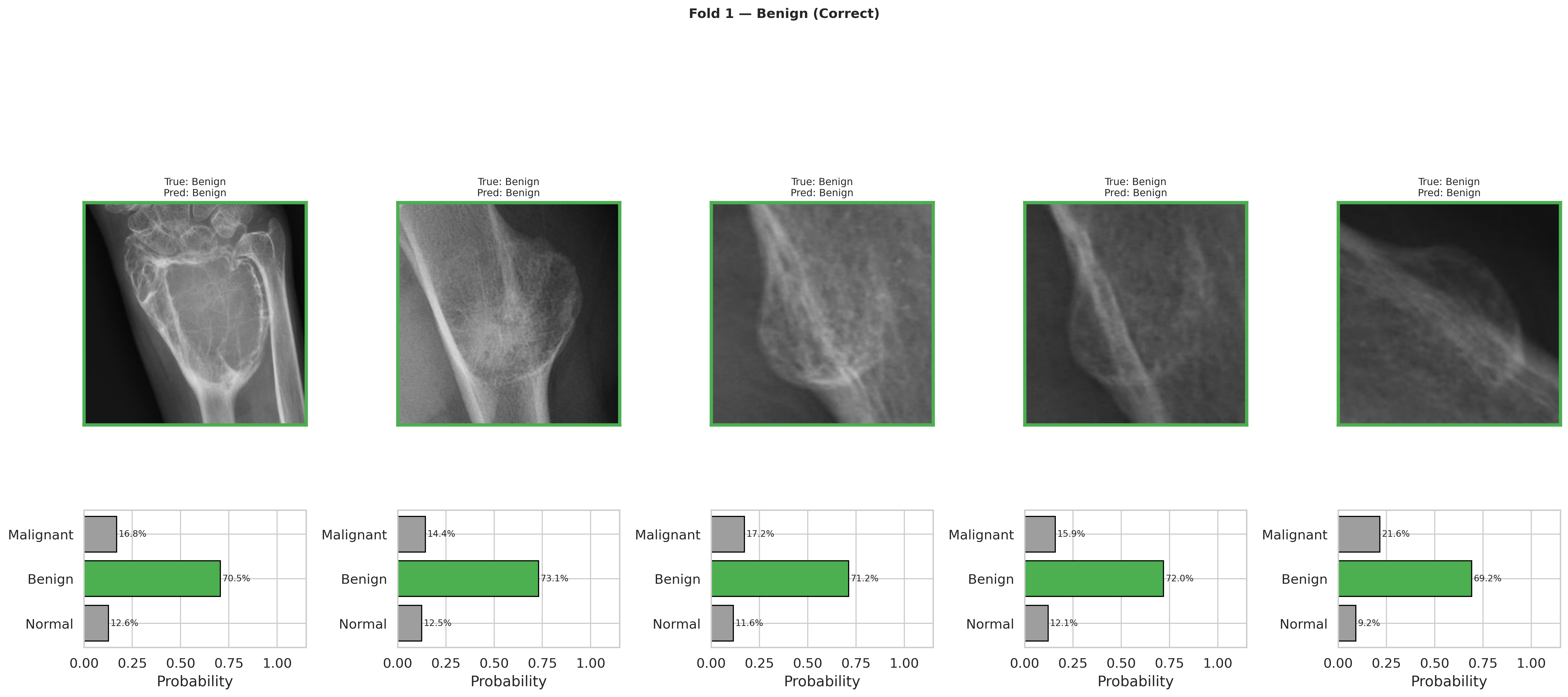

Supplement: Supplementary file 1 [file diagnostics-16-01811-s001.zip › Figure_S4_fold1_Benign_correct.png]

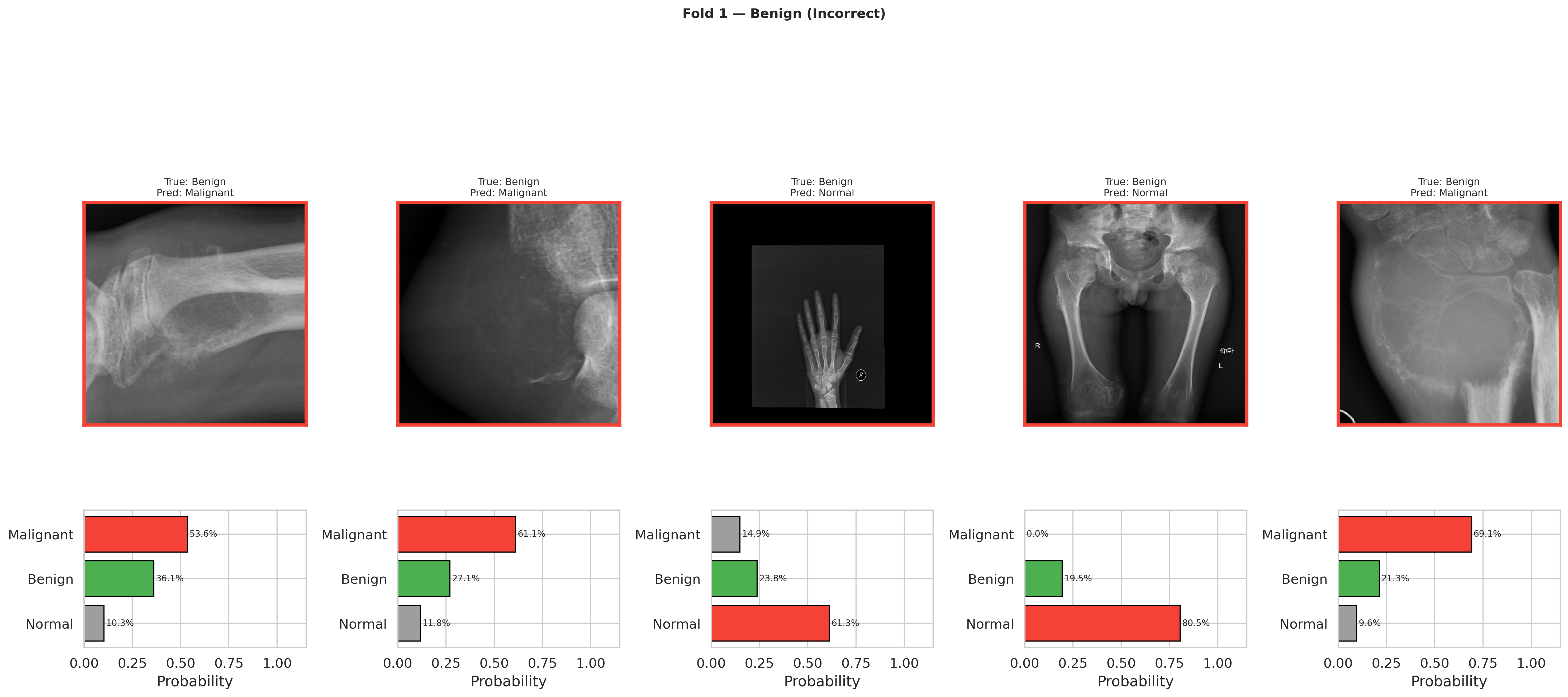

Supplement: Supplementary file 1 [file diagnostics-16-01811-s001.zip › Figure_S4_fold1_Benign_incorrect.png]

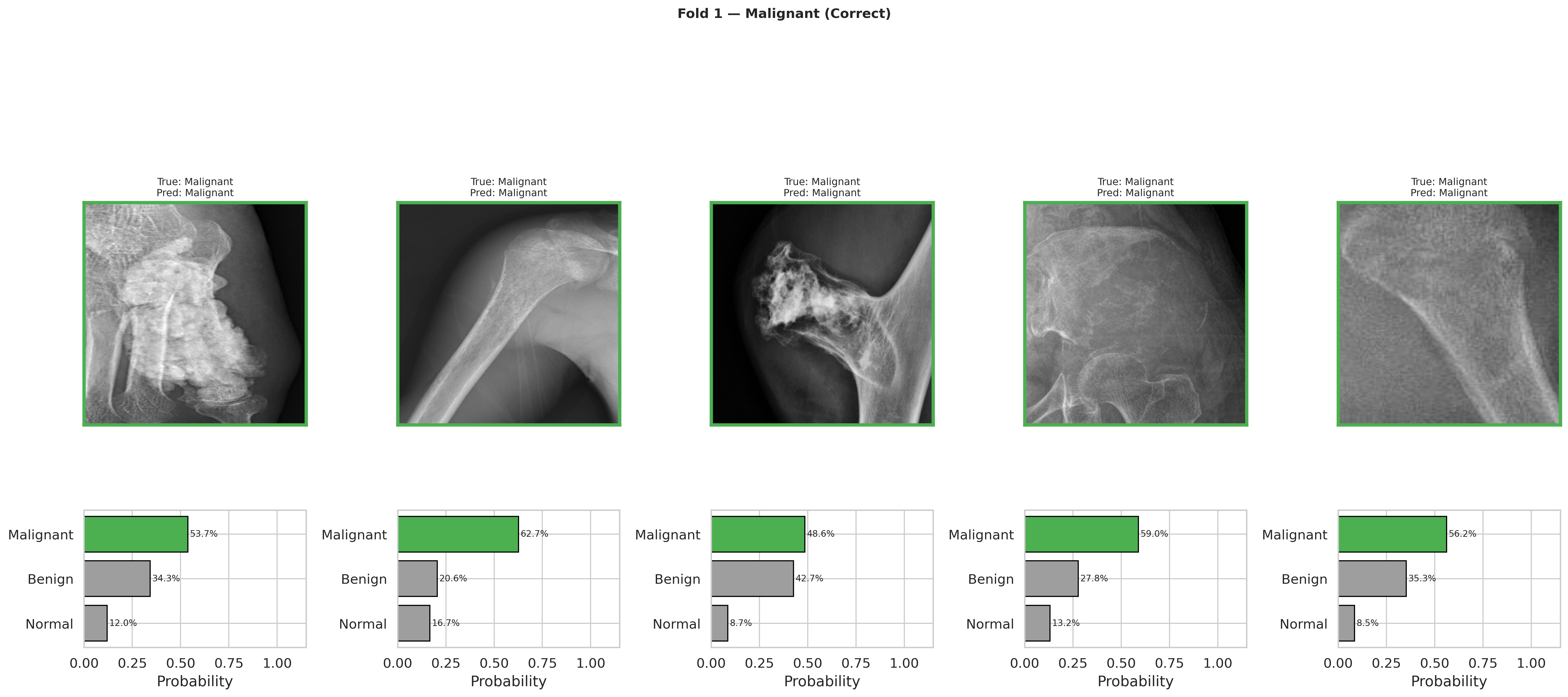

Supplement: Supplementary file 1 [file diagnostics-16-01811-s001.zip › Figure_S4_fold1_Malignant_correct.png]

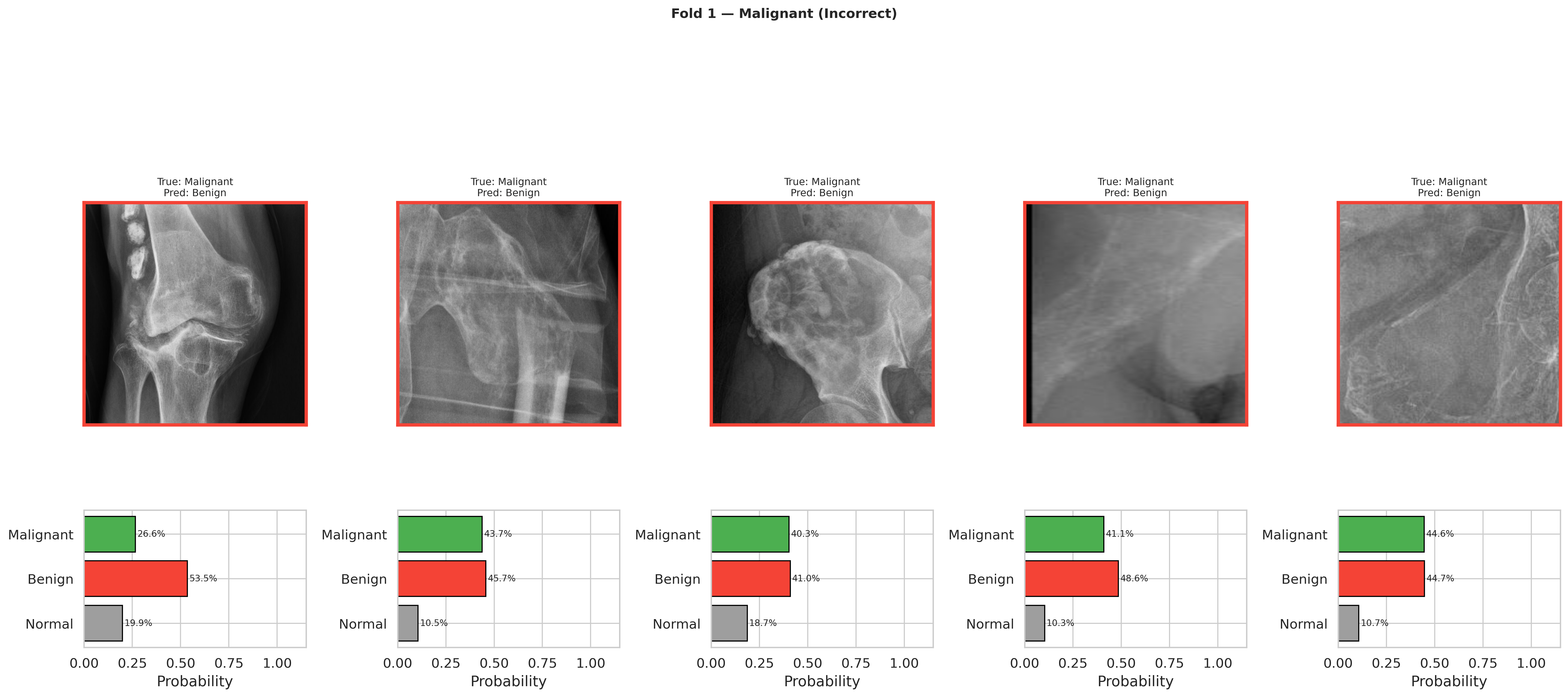

Supplement: Supplementary file 1 [file diagnostics-16-01811-s001.zip › Figure_S4_fold1_Malignant_incorrect.png]

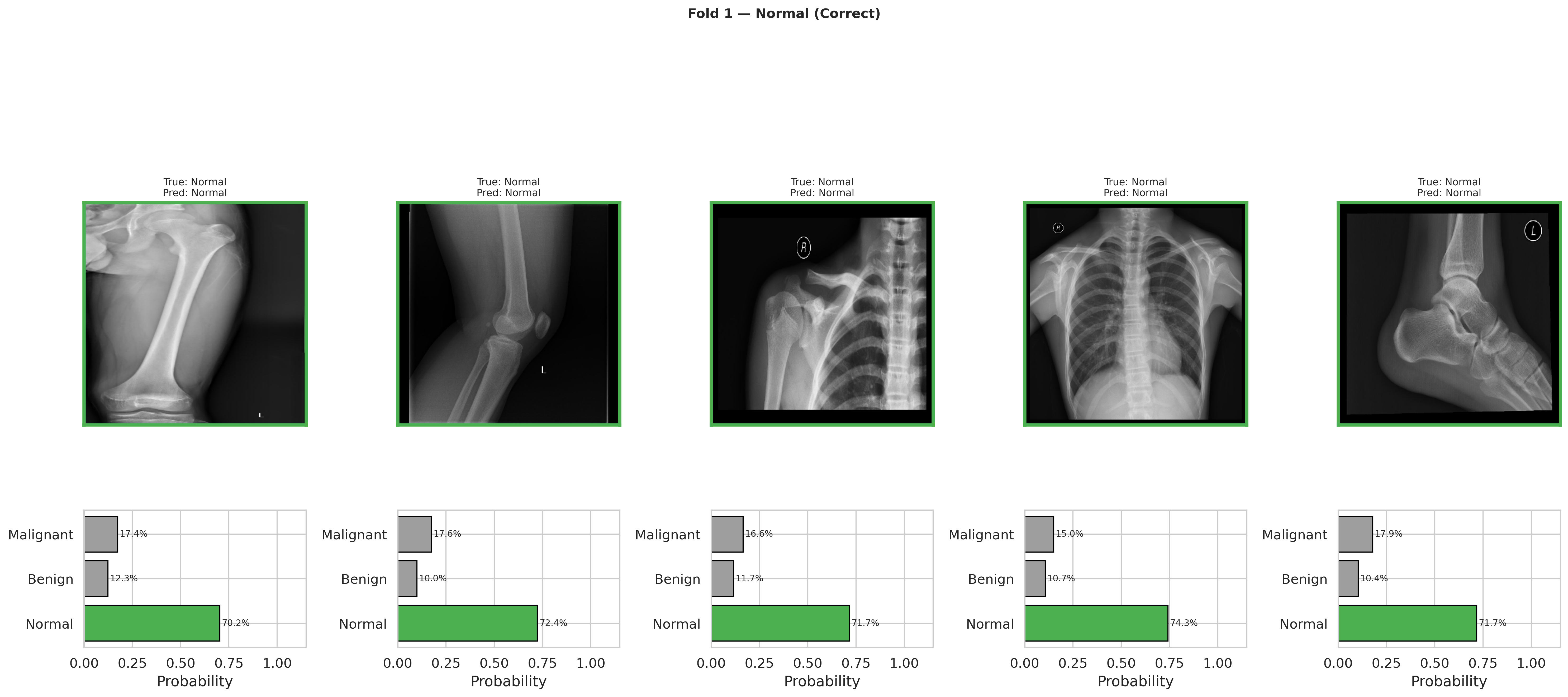

Supplement: Supplementary file 1 [file diagnostics-16-01811-s001.zip › Figure_S4_fold1_Normal_correct.png]

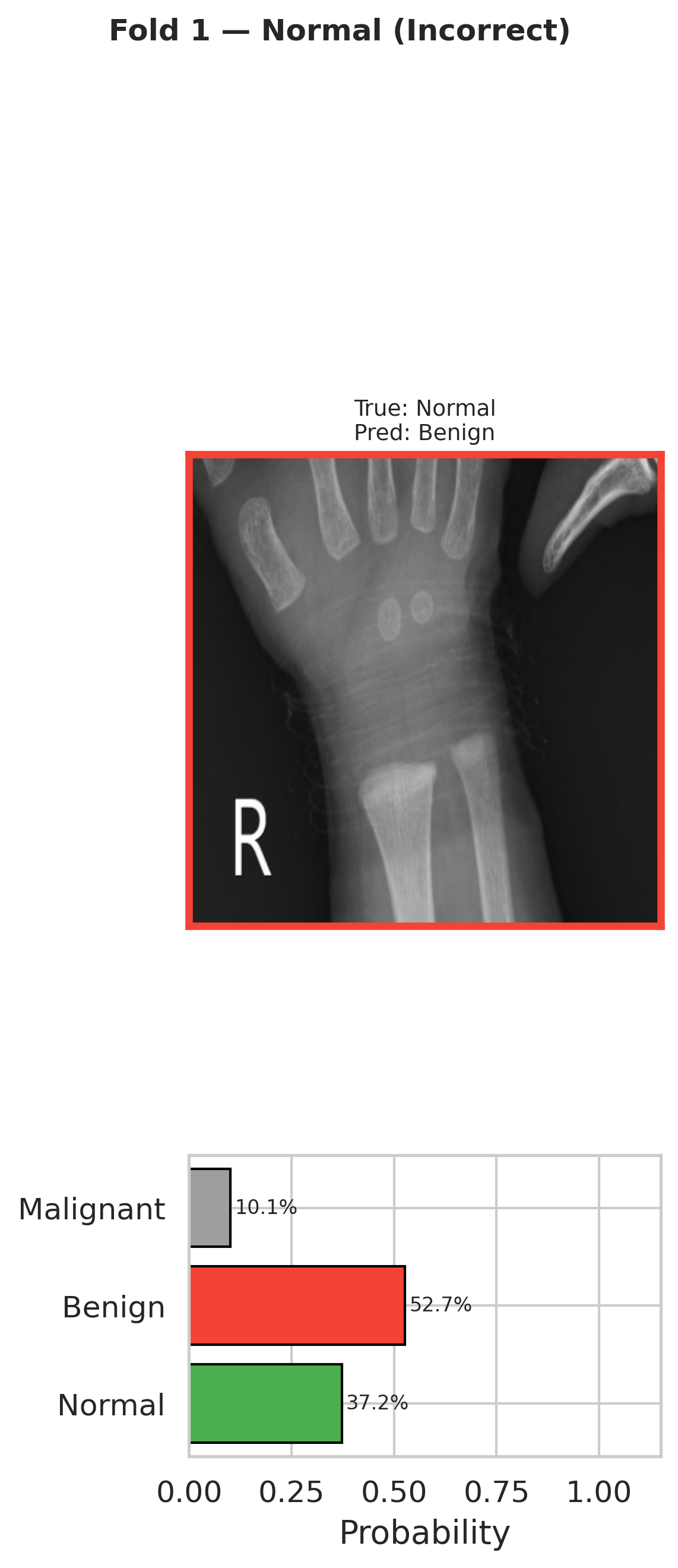

Supplement: Supplementary file 1 [file diagnostics-16-01811-s001.zip › Figure_S4_fold1_Normal_incorrect.png]

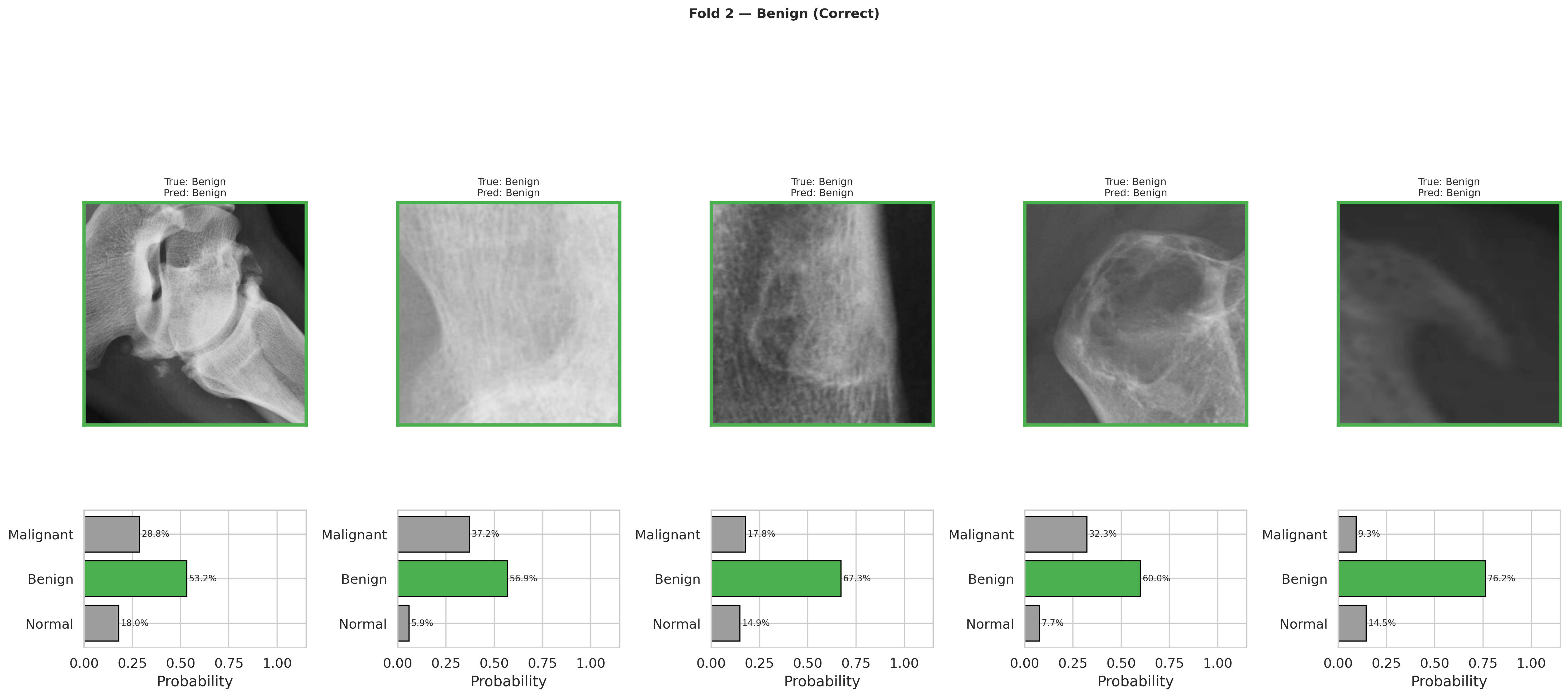

Supplement: Supplementary file 1 [file diagnostics-16-01811-s001.zip › Figure_S5_fold2_Benign_correct.png]

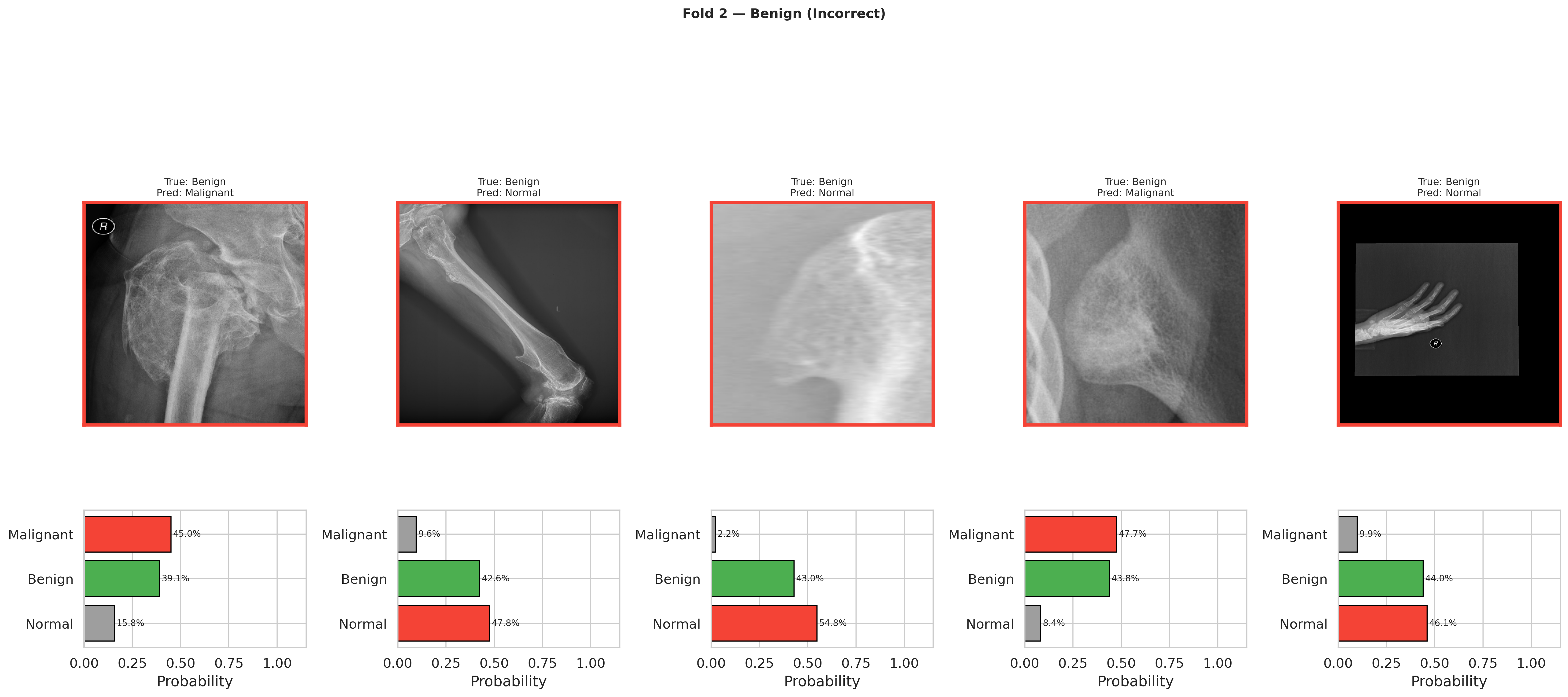

Supplement: Supplementary file 1 [file diagnostics-16-01811-s001.zip › Figure_S5_fold2_Benign_incorrect.png]

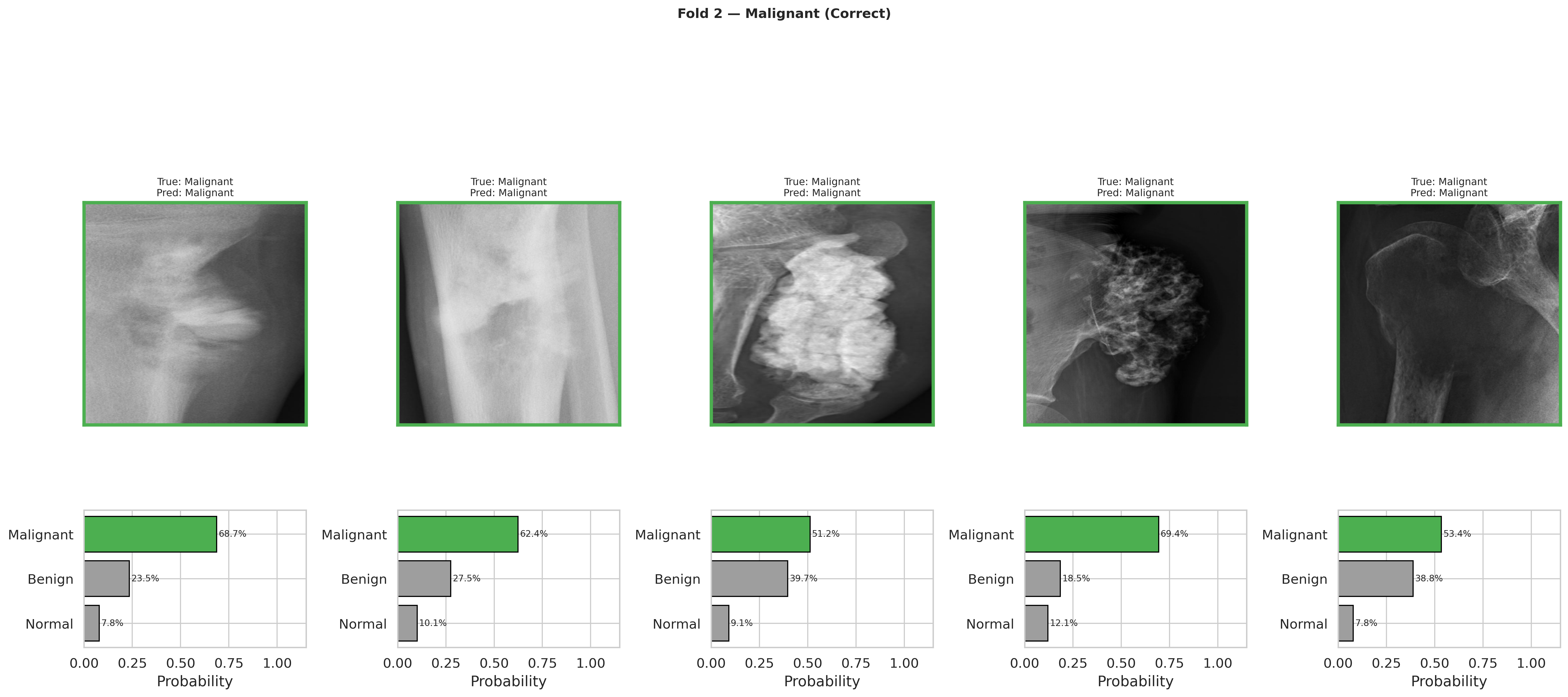

Supplement: Supplementary file 1 [file diagnostics-16-01811-s001.zip › Figure_S5_fold2_Malignant_correct.png]

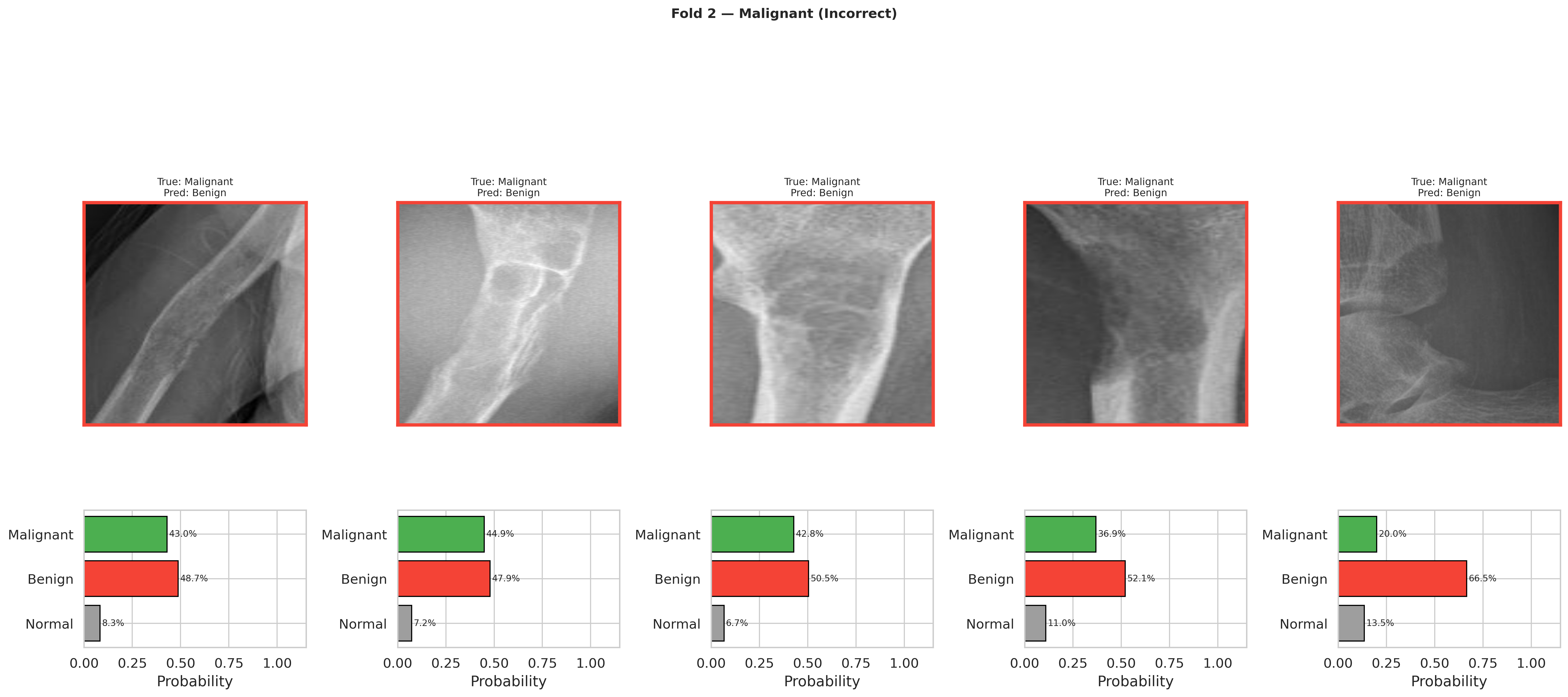

Supplement: Supplementary file 1 [file diagnostics-16-01811-s001.zip › Figure_S5_fold2_Malignant_incorrect.png]

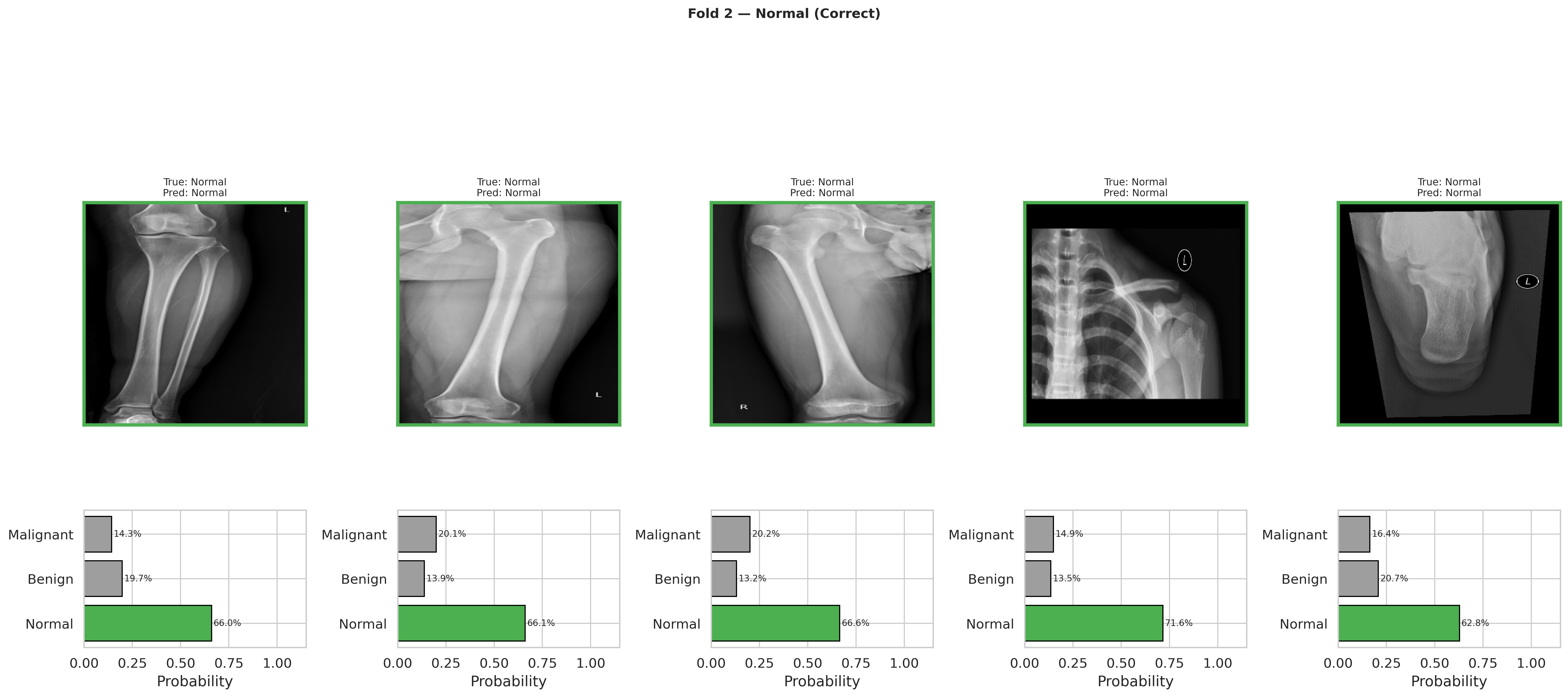

Supplement: Supplementary file 1 [file diagnostics-16-01811-s001.zip › Figure_S5_fold2_Normal_correct.png]

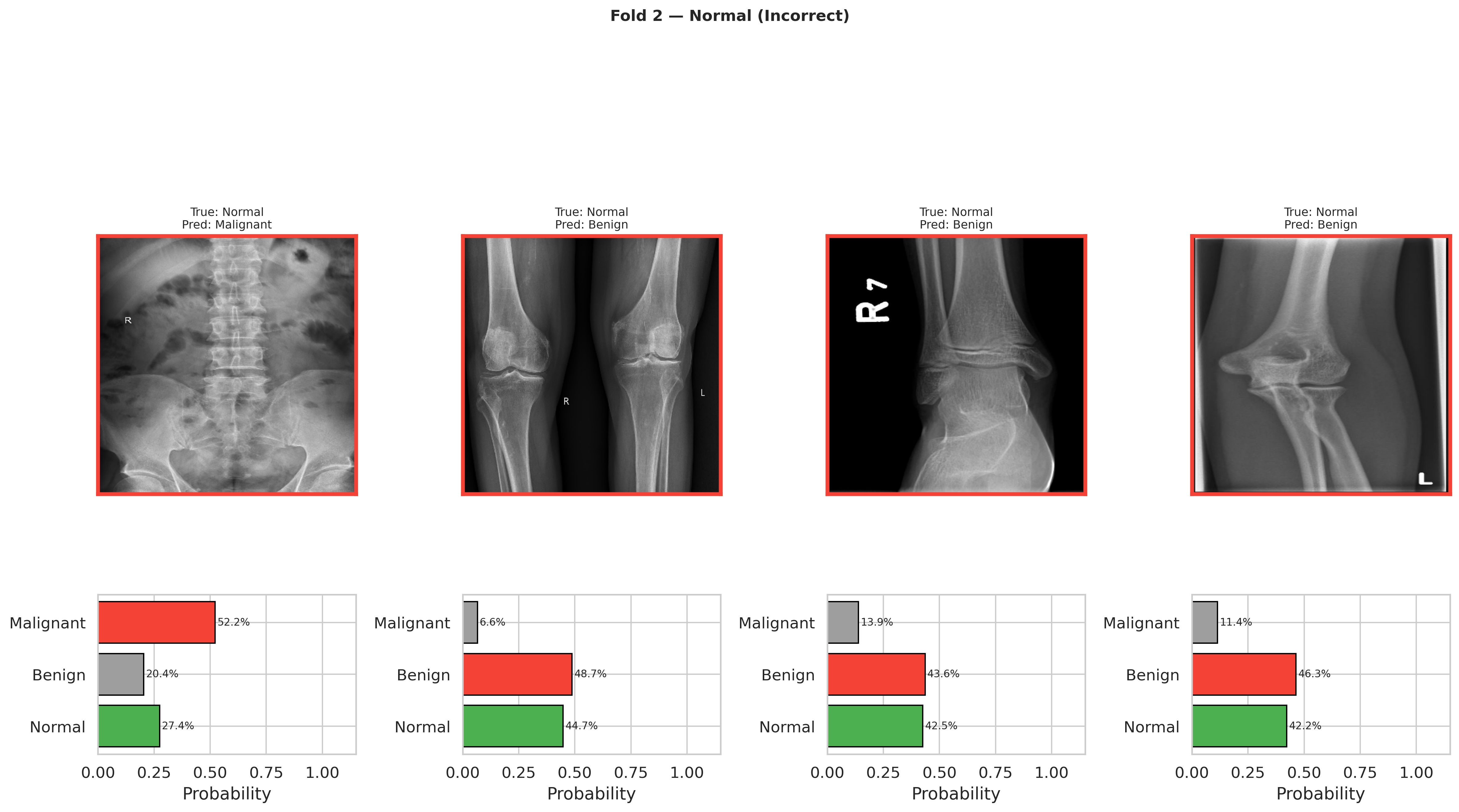

Supplement: Supplementary file 1 [file diagnostics-16-01811-s001.zip › Figure_S5_fold2_Normal_incorrect.png]

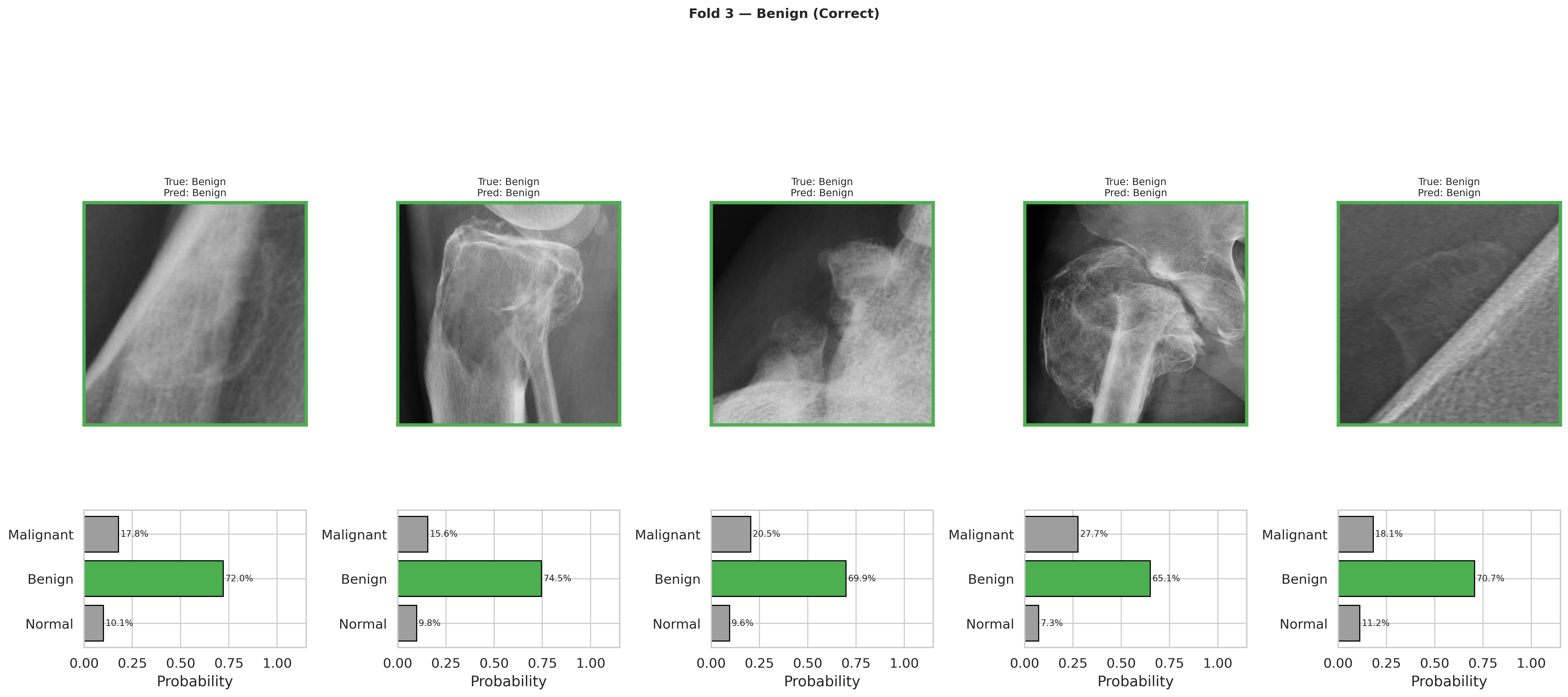

Supplement: Supplementary file 1 [file diagnostics-16-01811-s001.zip › Figure_S6_fold3_Benign_correct.png]

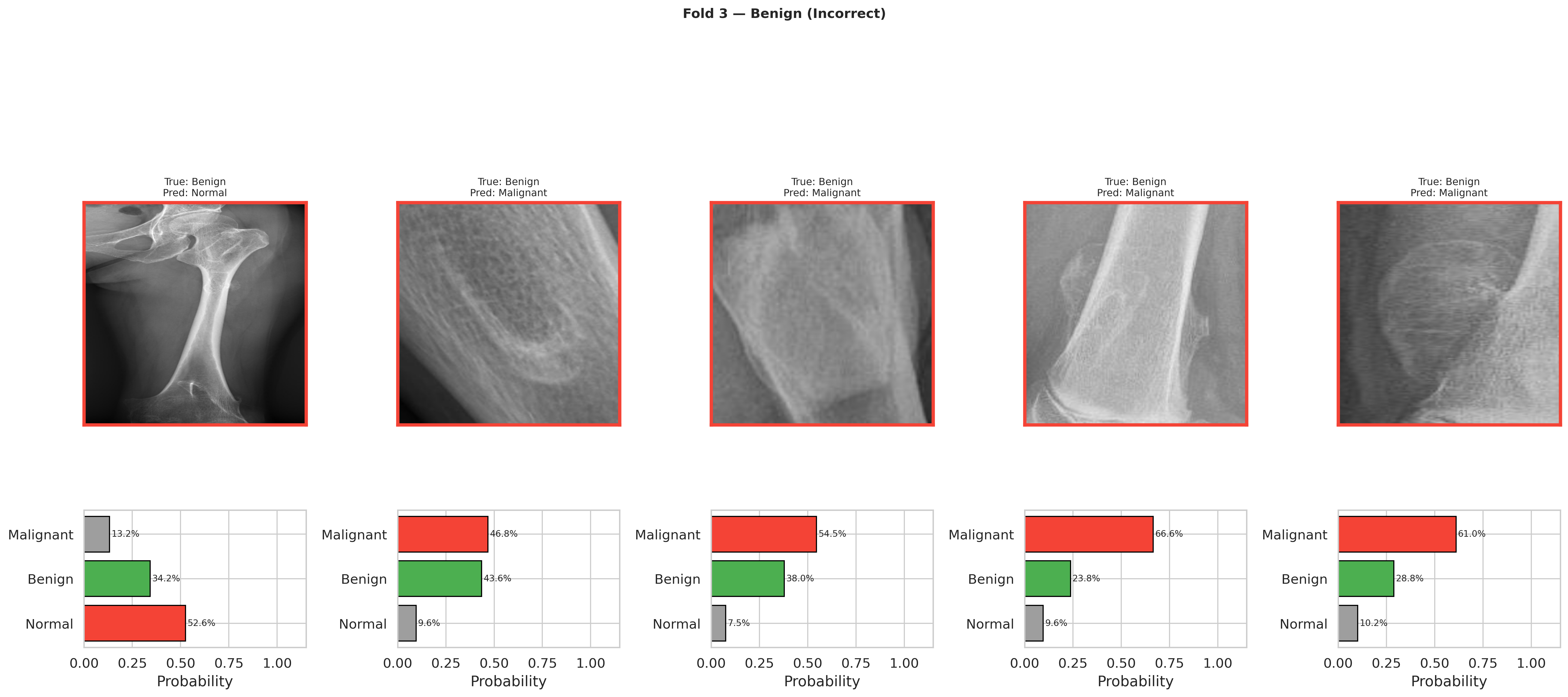

Supplement: Supplementary file 1 [file diagnostics-16-01811-s001.zip › Figure_S6_fold3_Benign_incorrect.png]

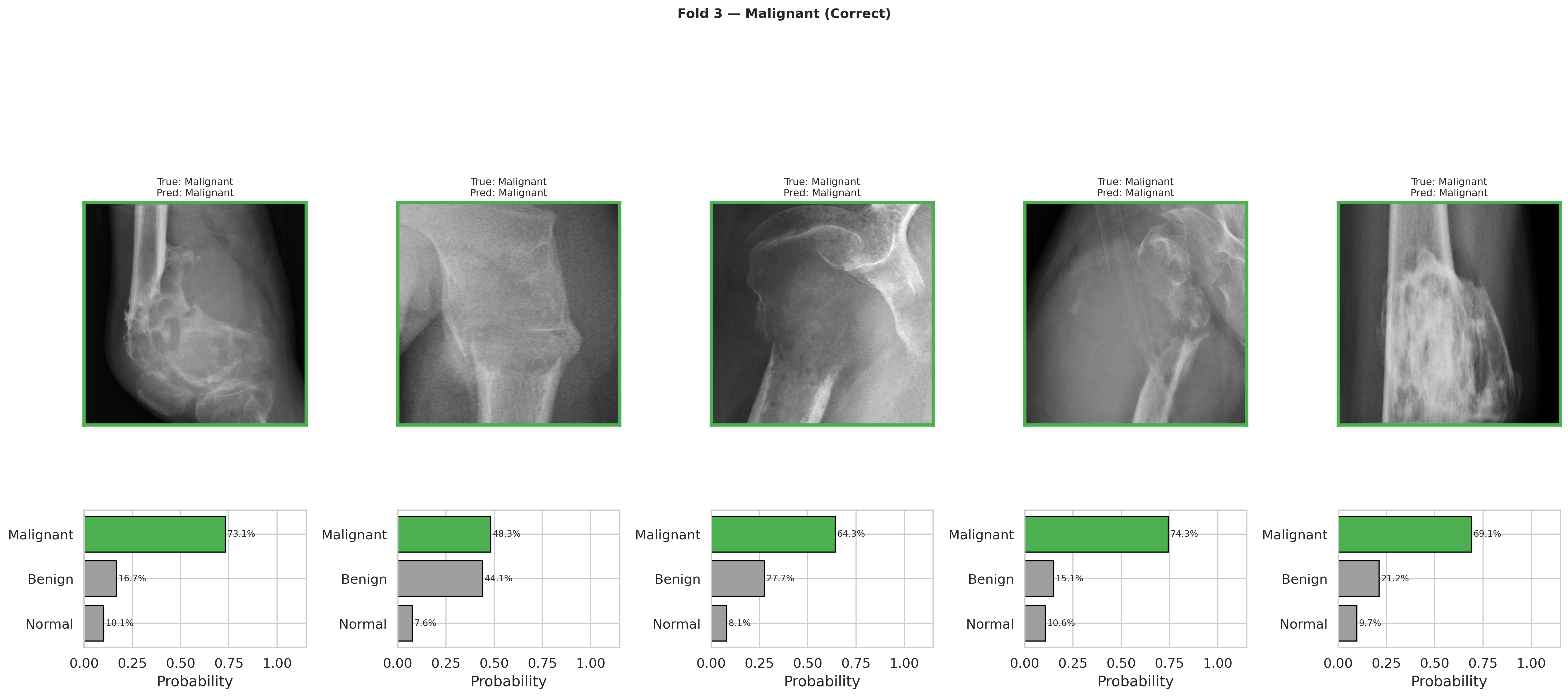

Supplement: Supplementary file 1 [file diagnostics-16-01811-s001.zip › Figure_S6_fold3_Malignant_correct.png]

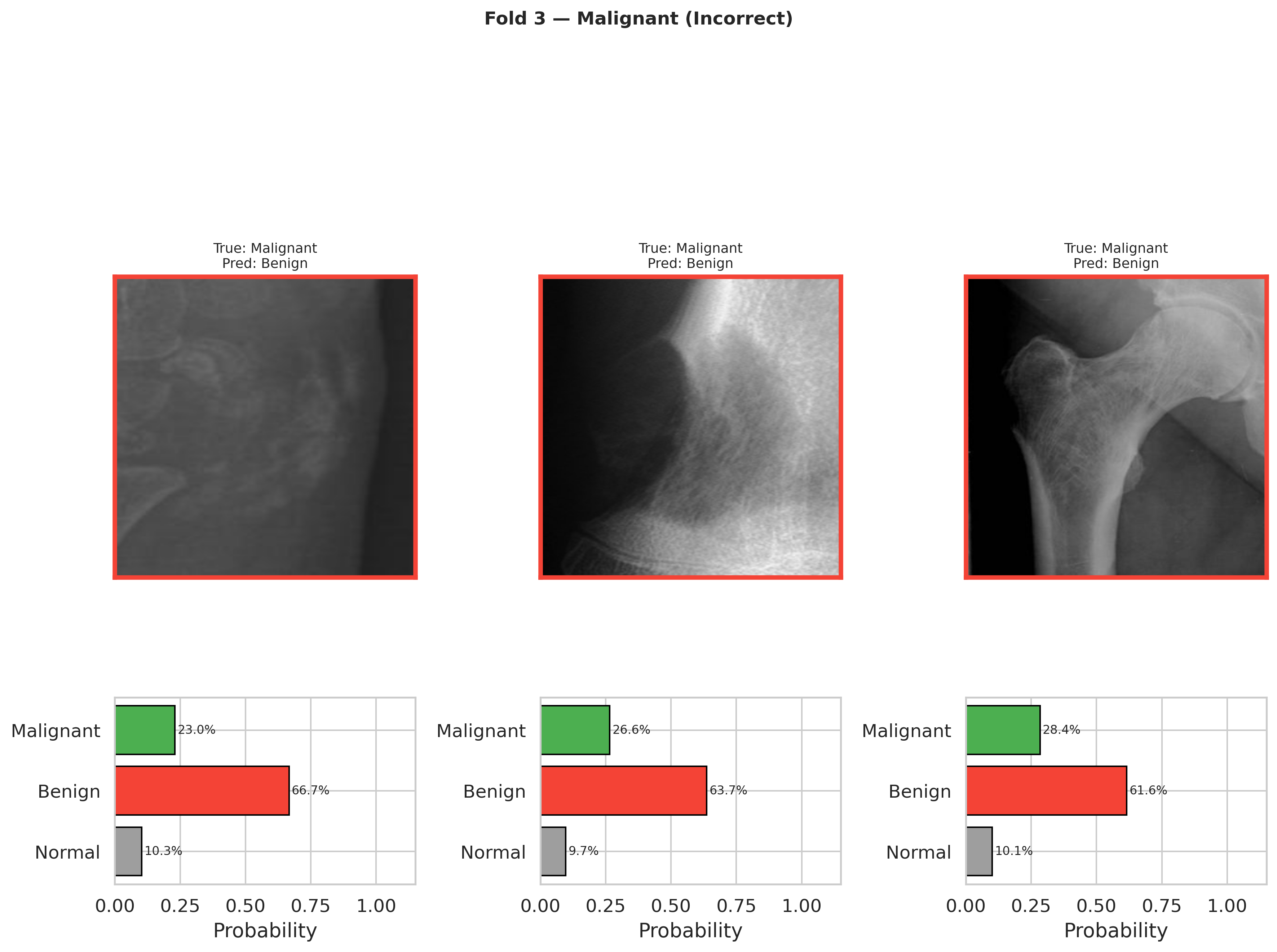

Supplement: Supplementary file 1 [file diagnostics-16-01811-s001.zip › Figure_S6_fold3_Malignant_incorrect.png]

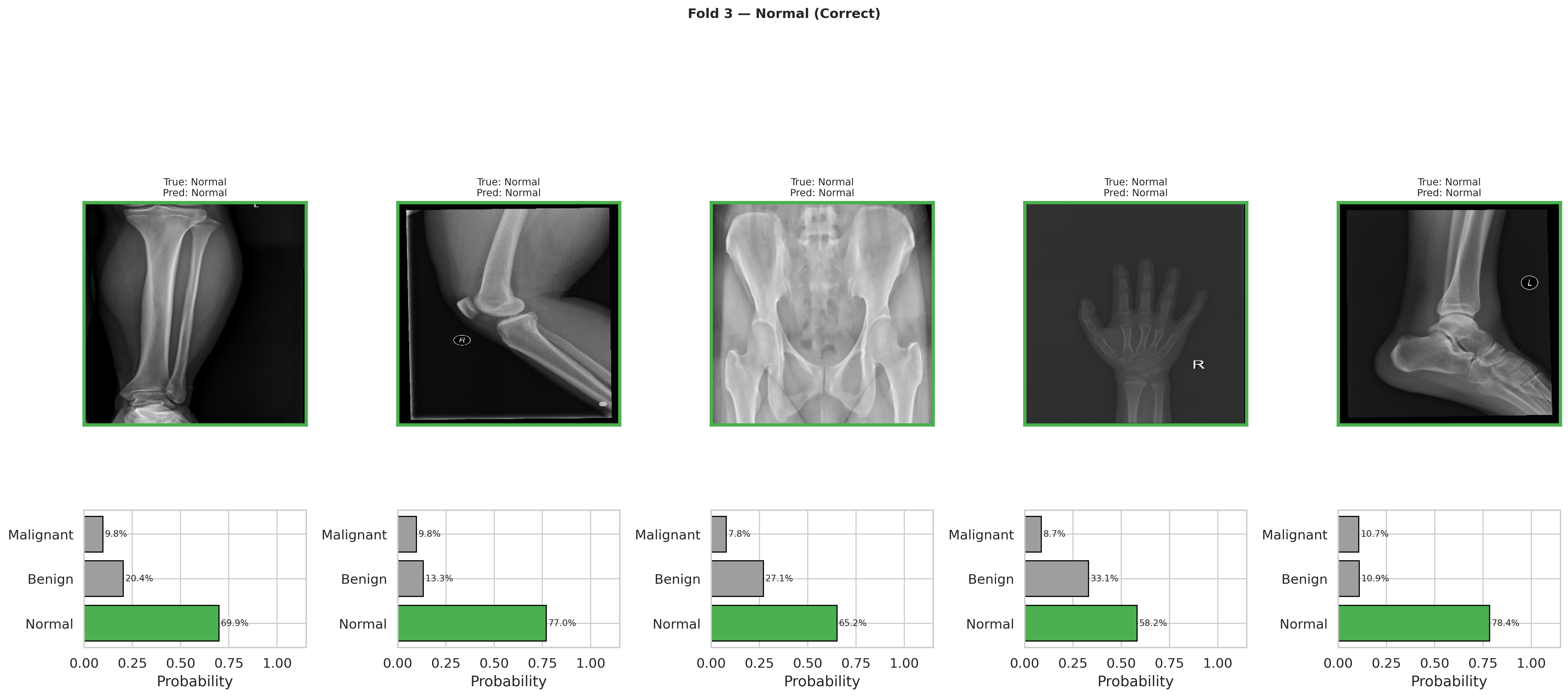

Supplement: Supplementary file 1 [file diagnostics-16-01811-s001.zip › Figure_S6_fold3_Normal_correct.png]

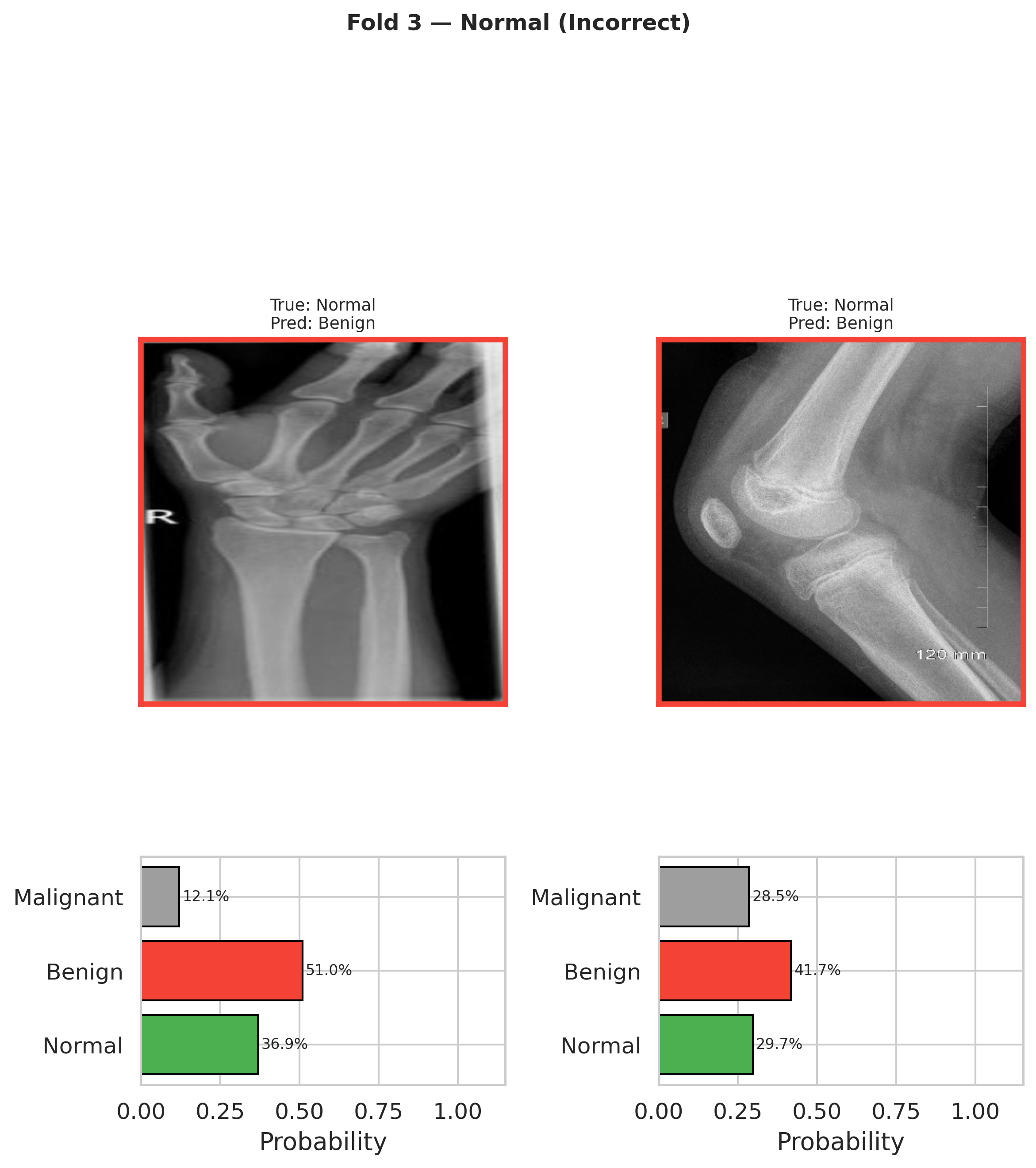

Supplement: Supplementary file 1 [file diagnostics-16-01811-s001.zip › Figure_S6_fold3_Normal_incorrect.png]

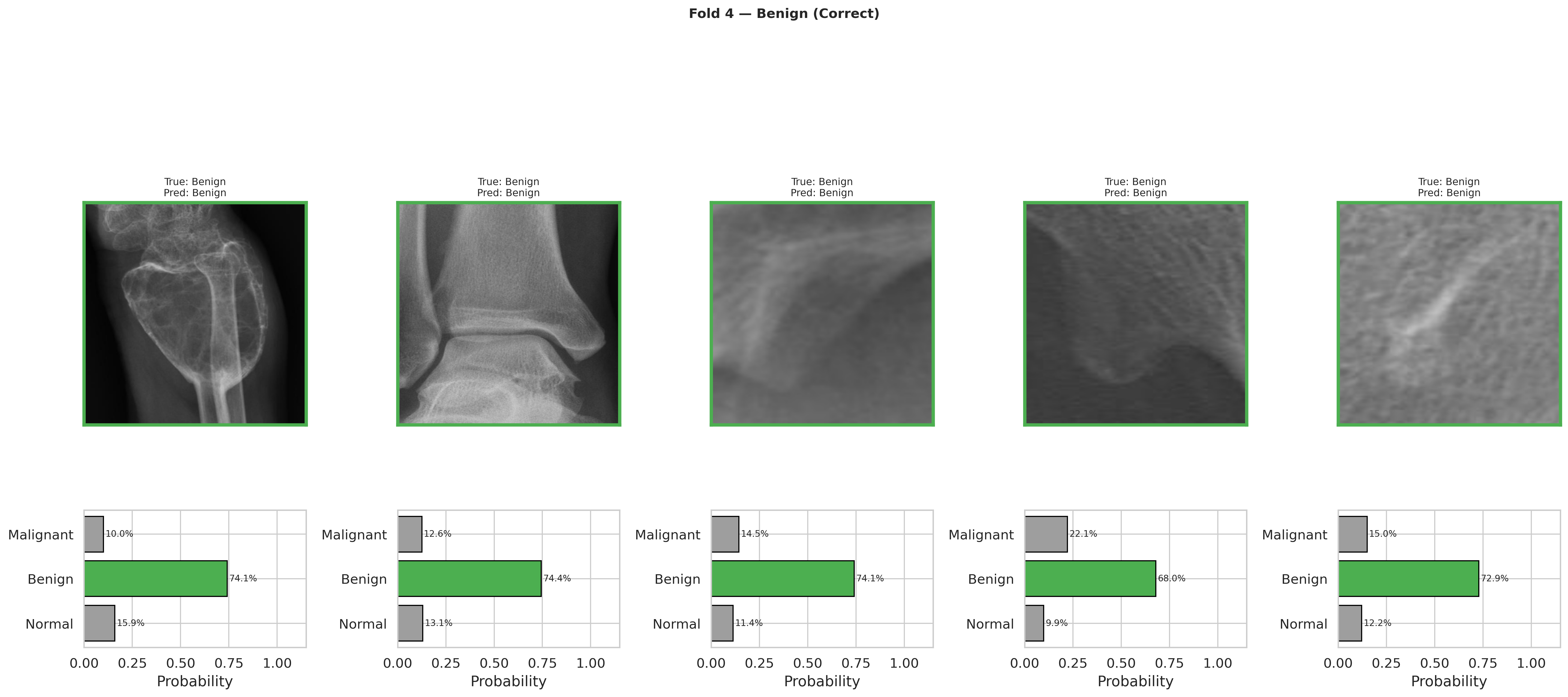

Supplement: Supplementary file 1 [file diagnostics-16-01811-s001.zip › Figure_S7_fold4_Benign_correct.png]

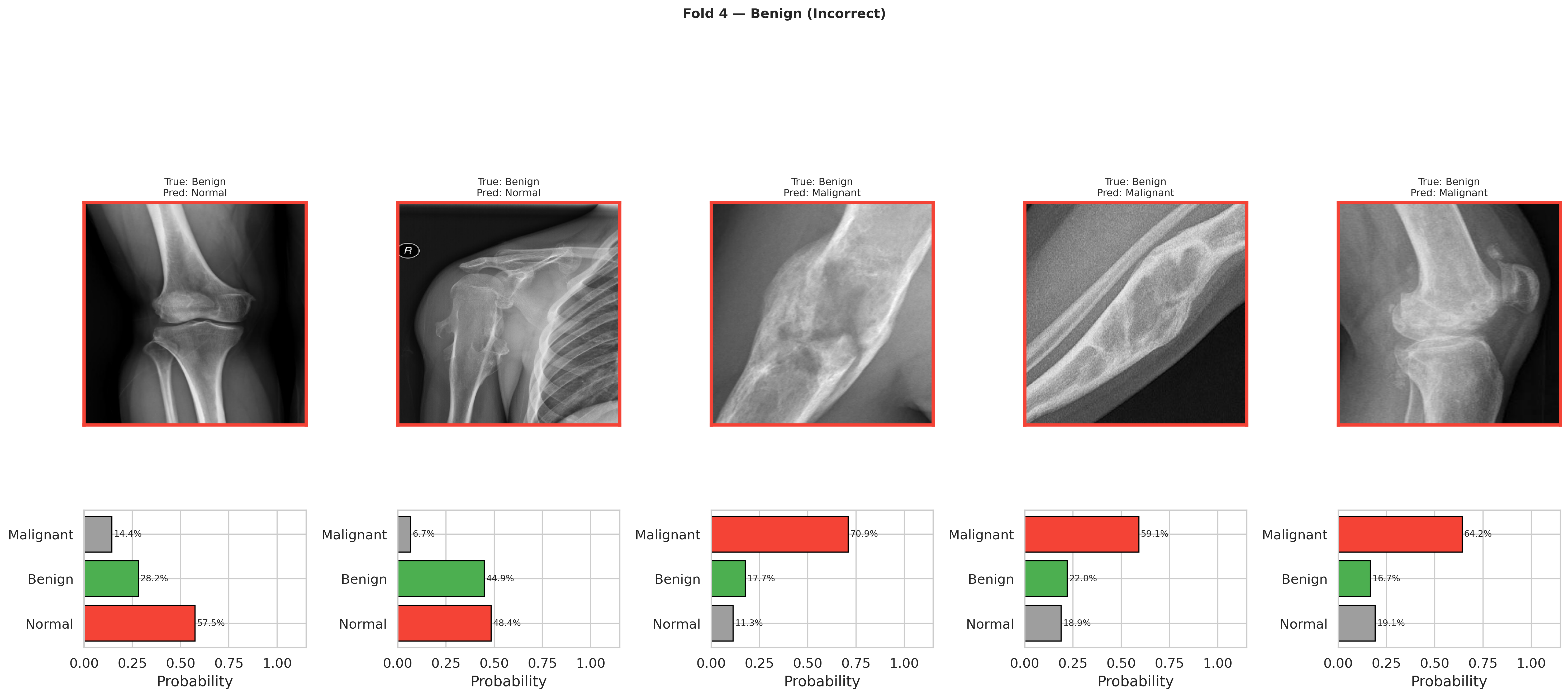

Supplement: Supplementary file 1 [file diagnostics-16-01811-s001.zip › Figure_S7_fold4_Benign_incorrect.png]

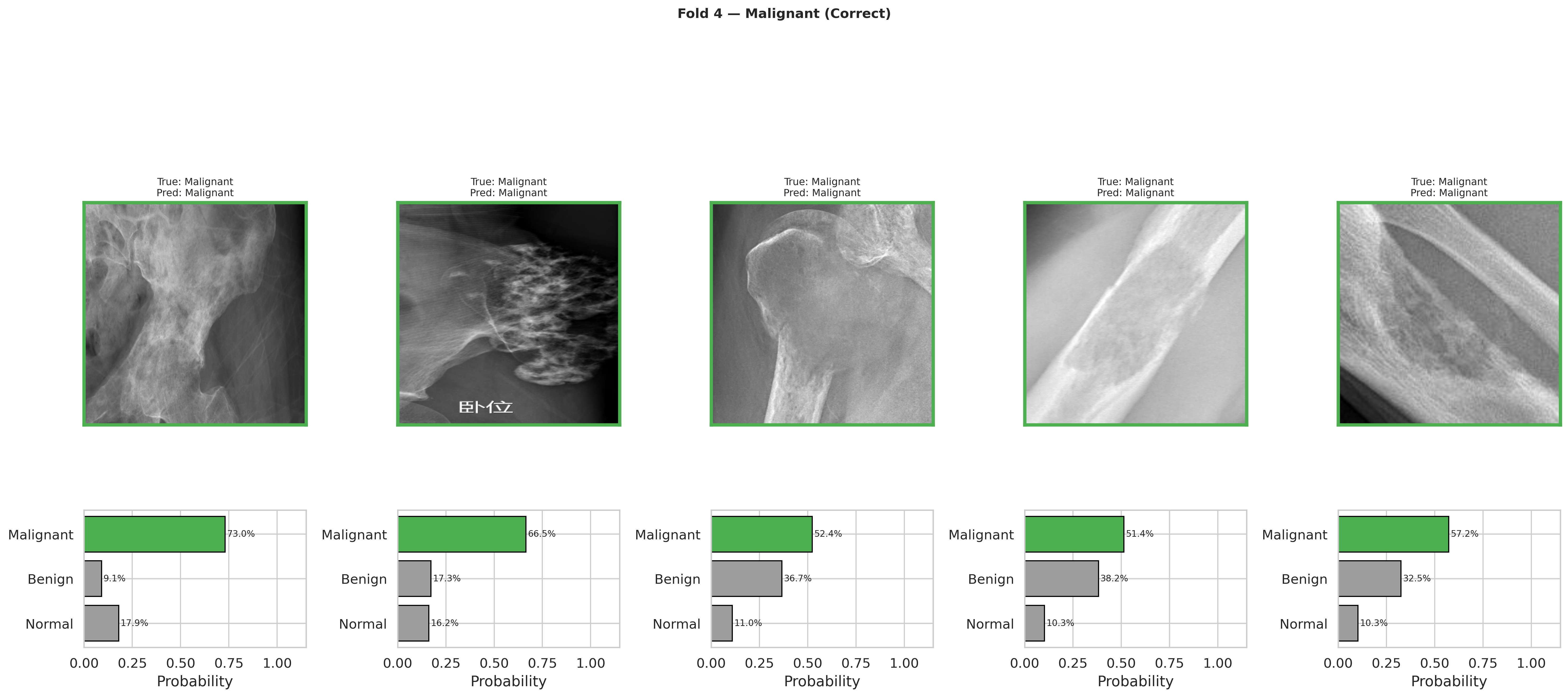

Supplement: Supplementary file 1 [file diagnostics-16-01811-s001.zip › Figure_S7_fold4_Malignant_correct.png]

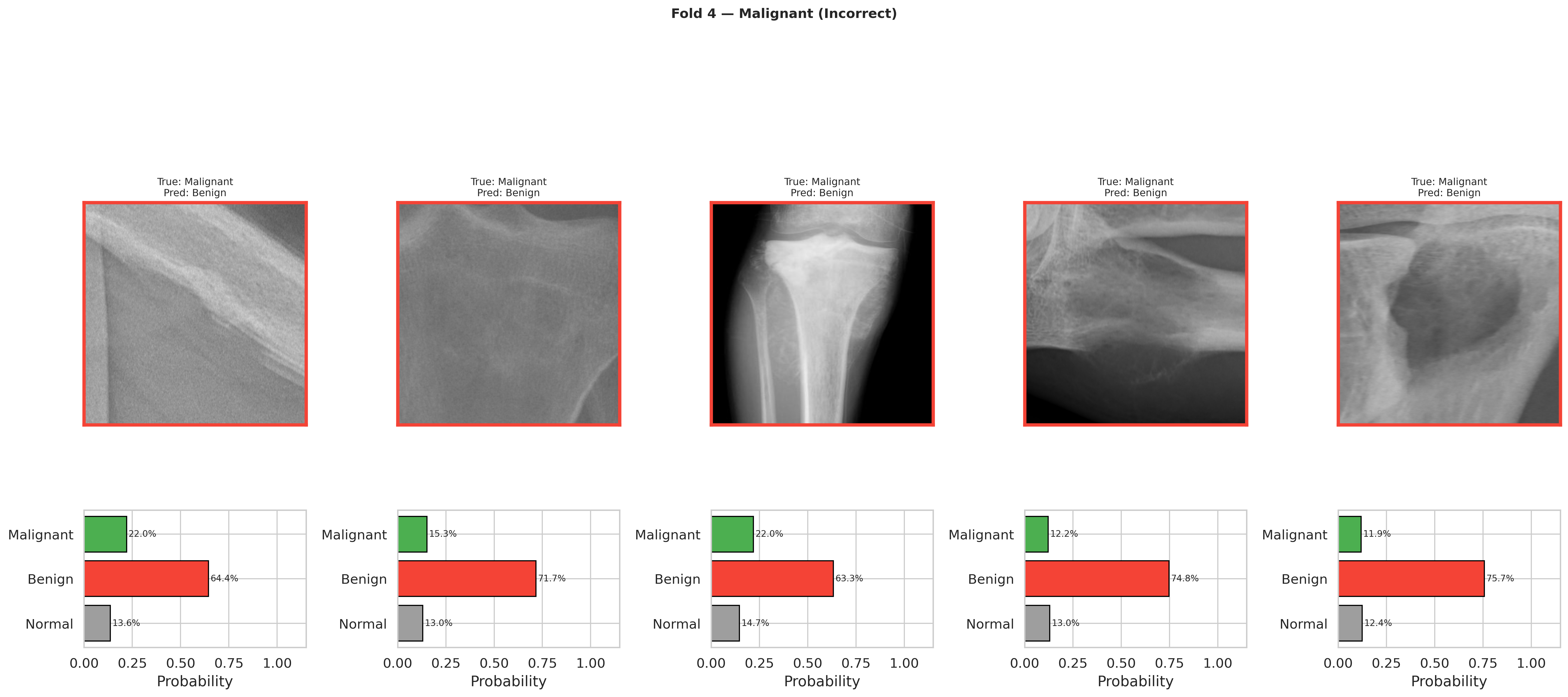

Supplement: Supplementary file 1 [file diagnostics-16-01811-s001.zip › Figure_S7_fold4_Malignant_incorrect.png]

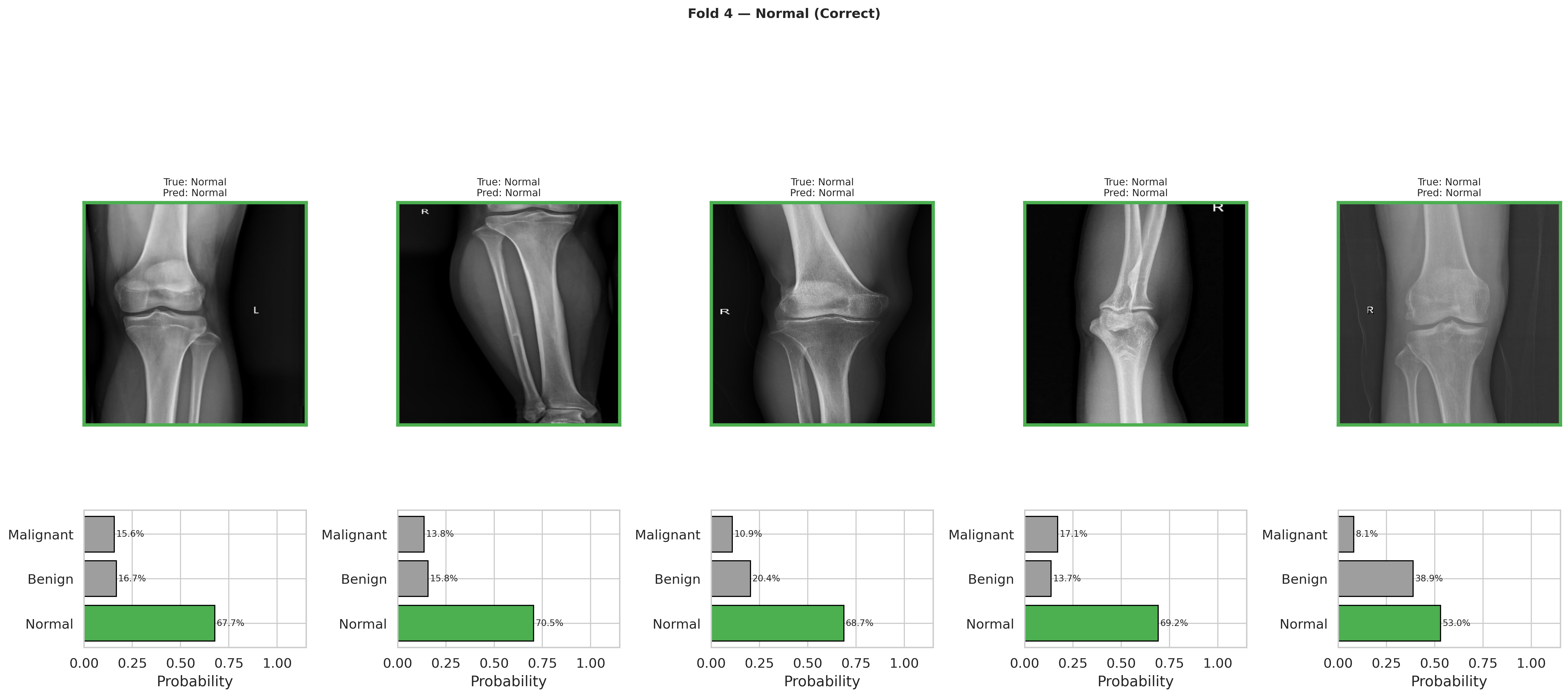

Supplement: Supplementary file 1 [file diagnostics-16-01811-s001.zip › Figure_S7_fold4_Normal_correct.png]

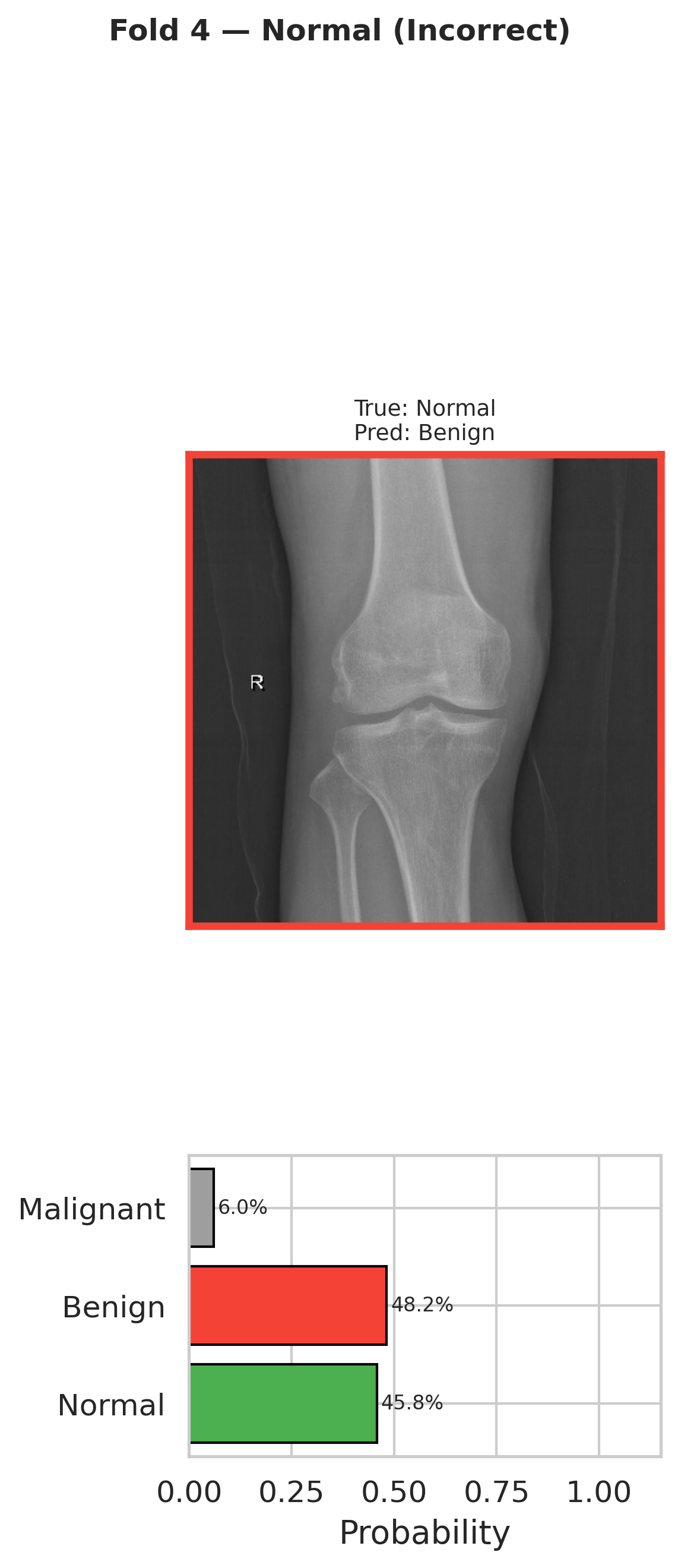

Supplement: Supplementary file 1 [file diagnostics-16-01811-s001.zip › Figure_S7_fold4_Normal_incorrect.png]

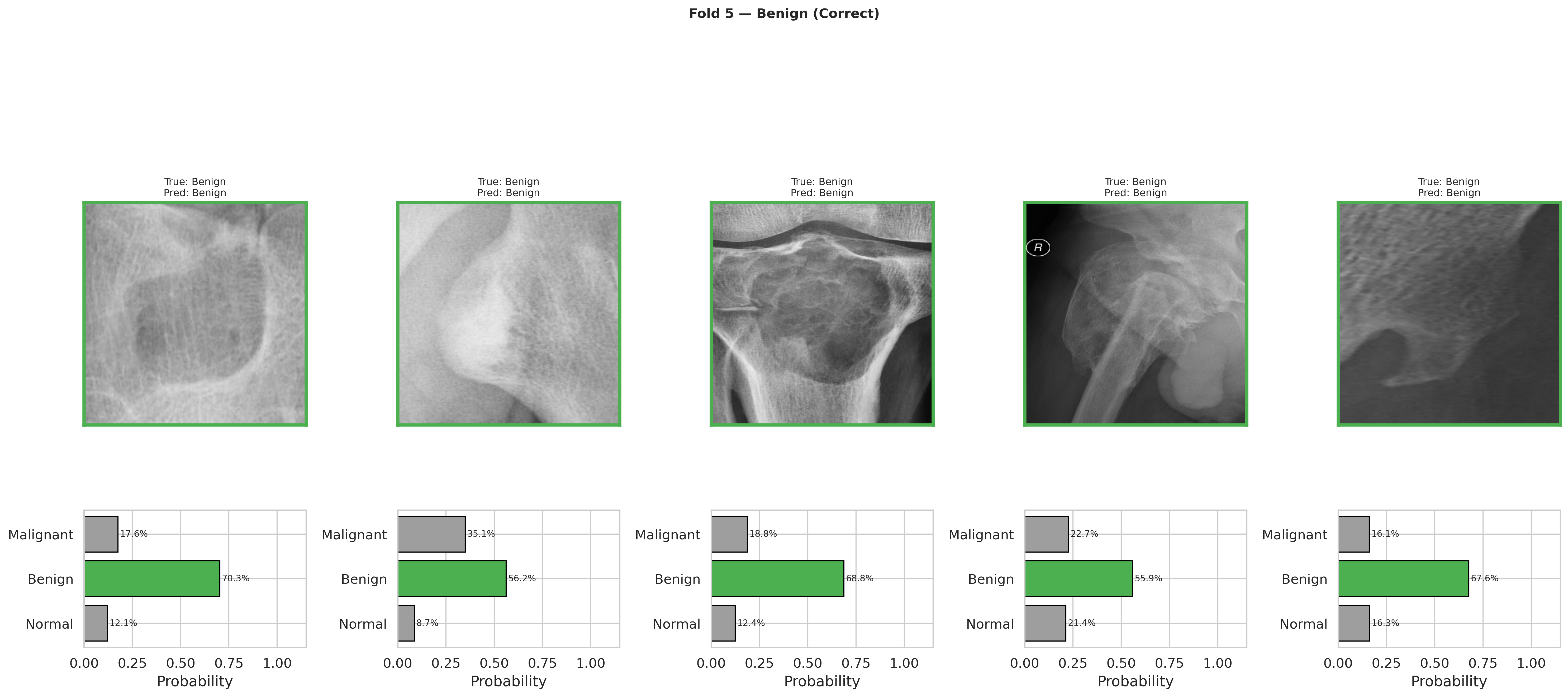

Supplement: Supplementary file 1 [file diagnostics-16-01811-s001.zip › Figure_S8_fold5_Benign_correct.png]

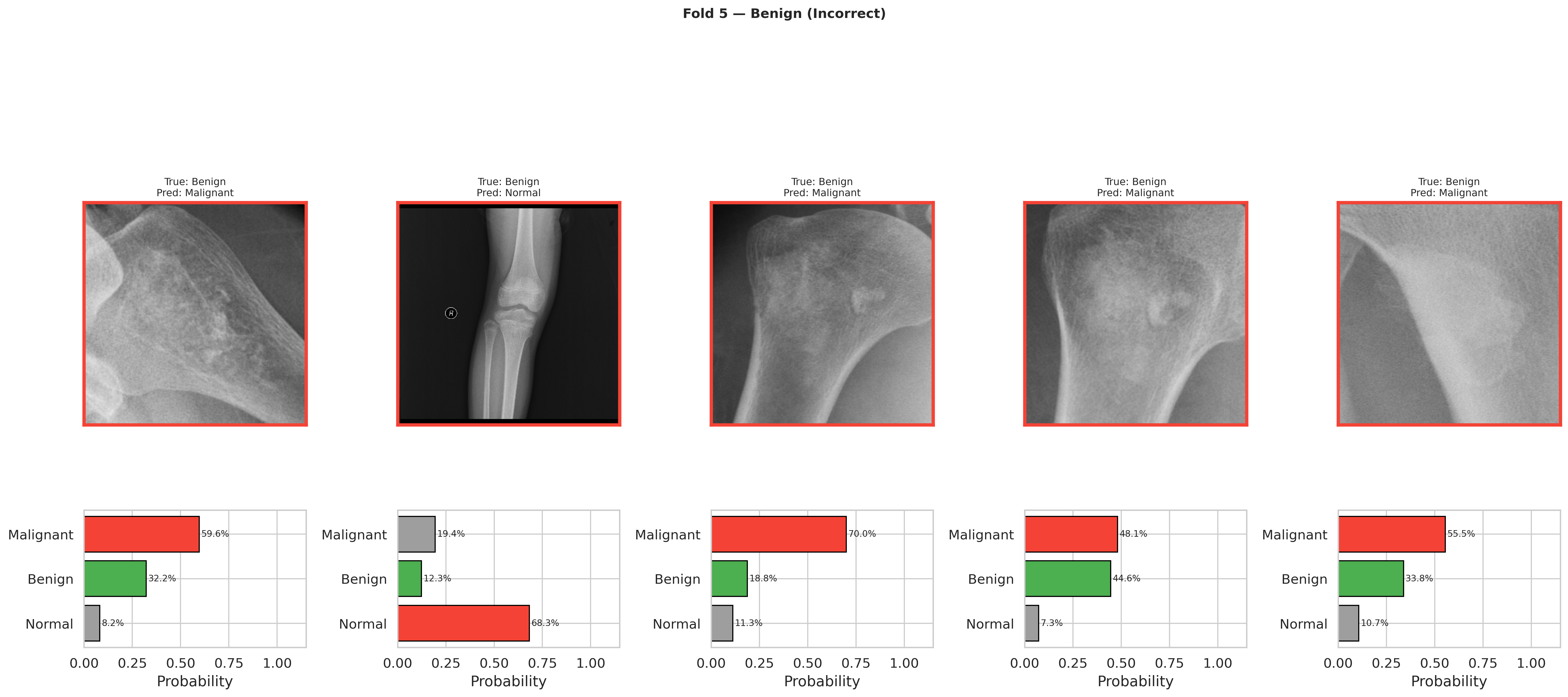

Supplement: Supplementary file 1 [file diagnostics-16-01811-s001.zip › Figure_S8_fold5_Benign_incorrect.png]

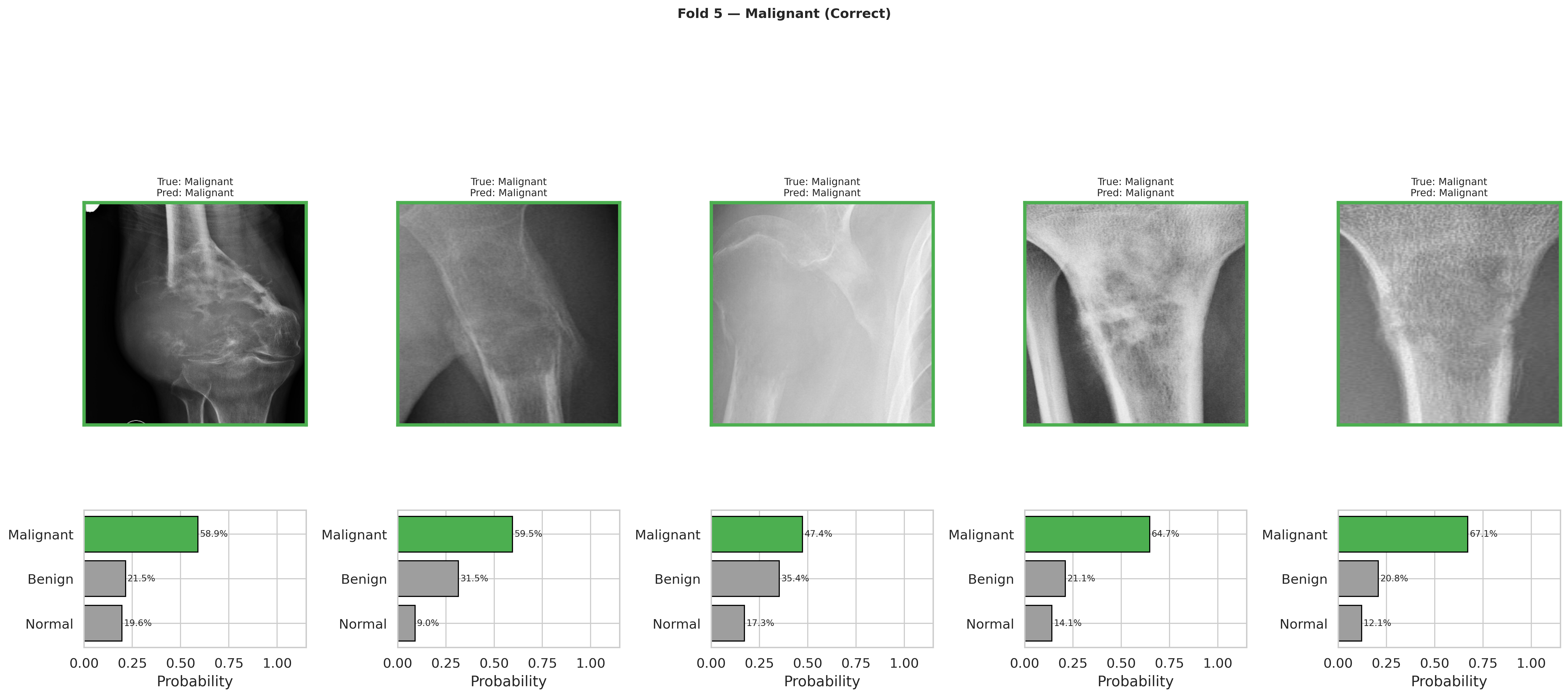

Supplement: Supplementary file 1 [file diagnostics-16-01811-s001.zip › Figure_S8_fold5_Malignant_correct.png]

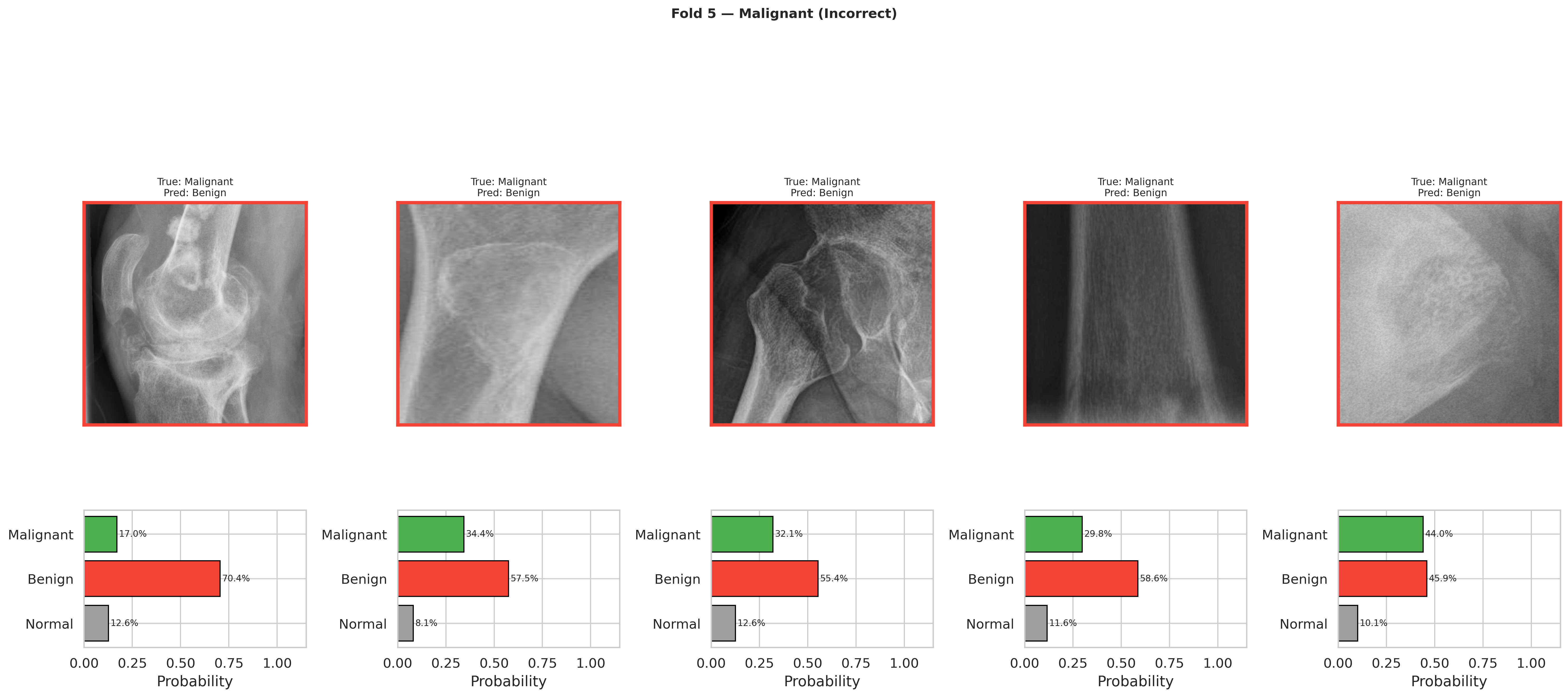

Supplement: Supplementary file 1 [file diagnostics-16-01811-s001.zip › Figure_S8_fold5_Malignant_incorrect.png]

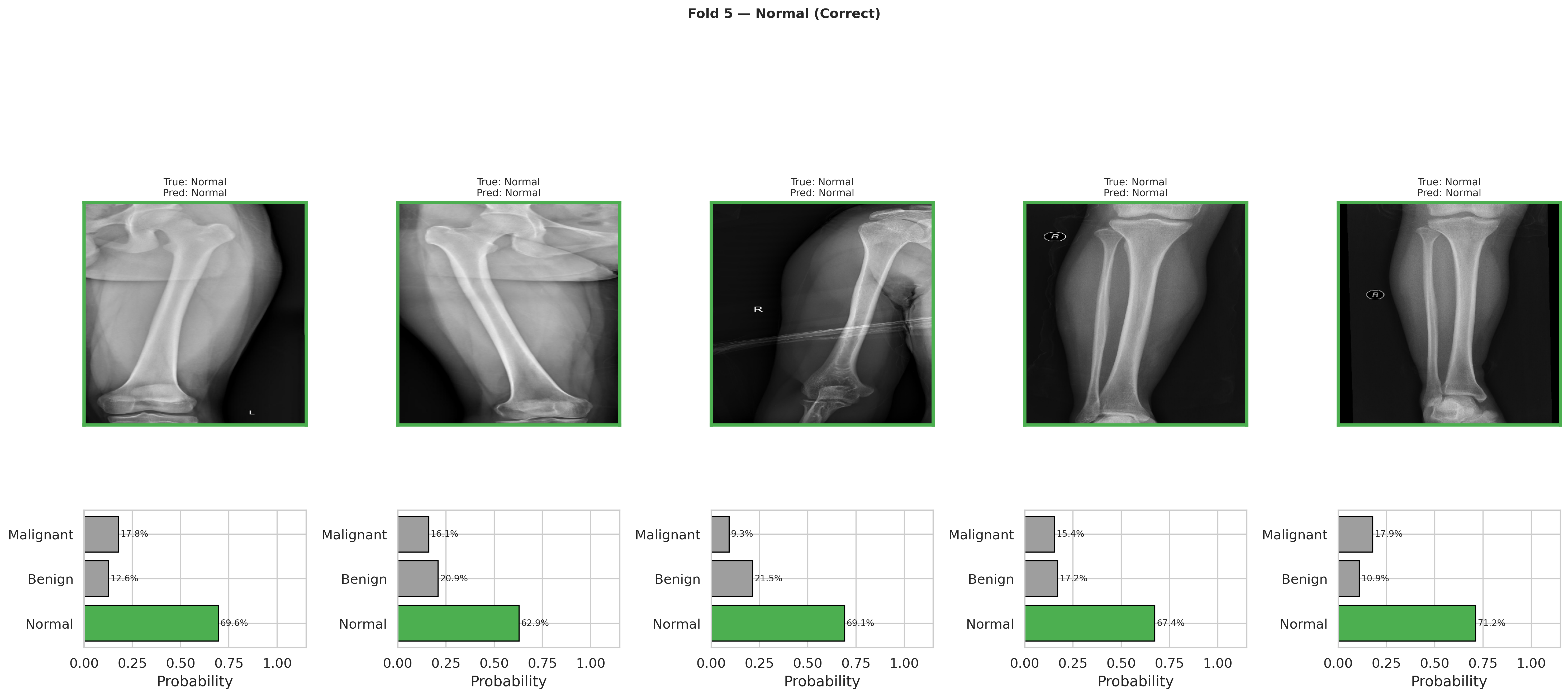

Supplement: Supplementary file 1 [file diagnostics-16-01811-s001.zip › Figure_S8_fold5_Normal_correct.png]

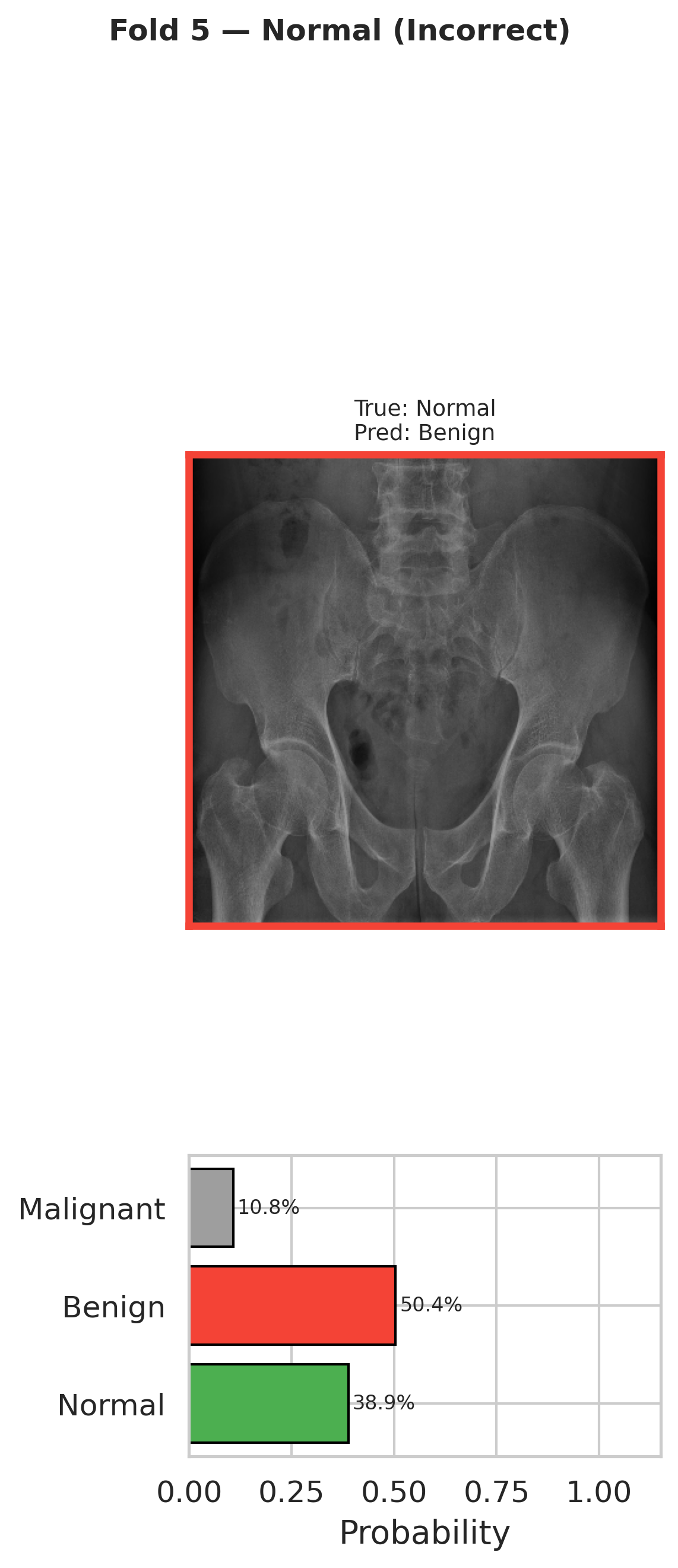

Supplement: Supplementary file 1 [file diagnostics-16-01811-s001.zip › Figure_S8_fold5_Normal_incorrect.png]

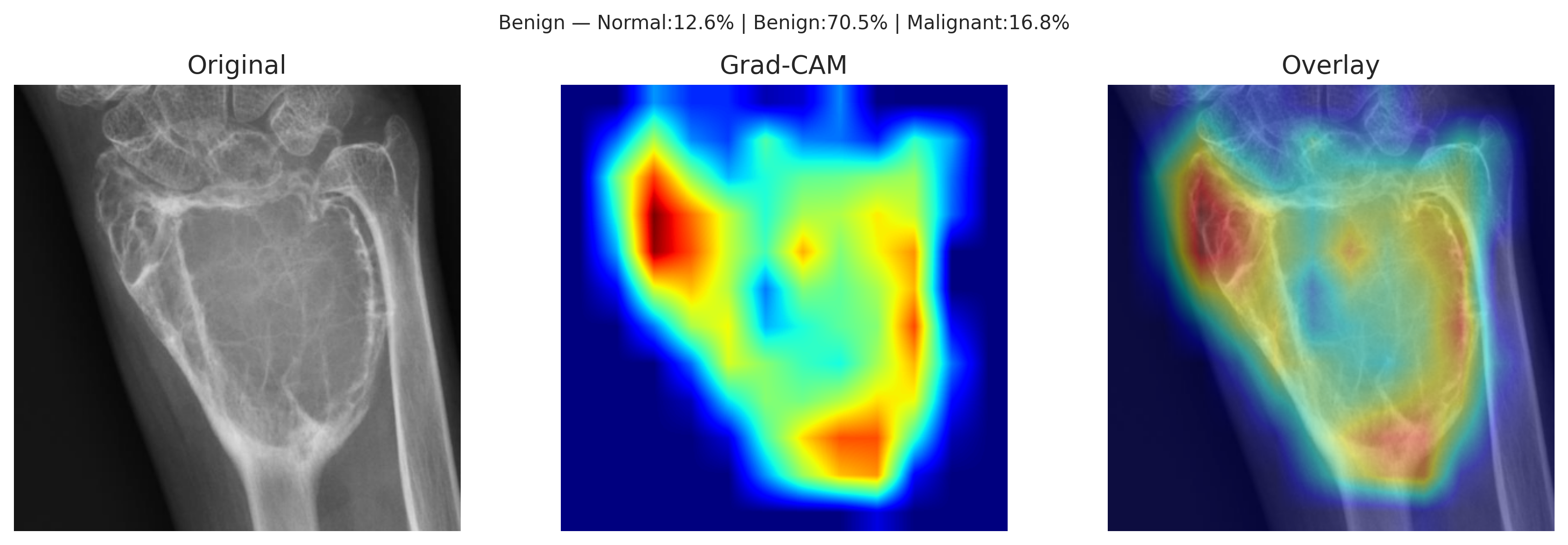

Supplement: Supplementary file 1 [file diagnostics-16-01811-s001.zip › Figure_S9_fold1_Benign_0.png]

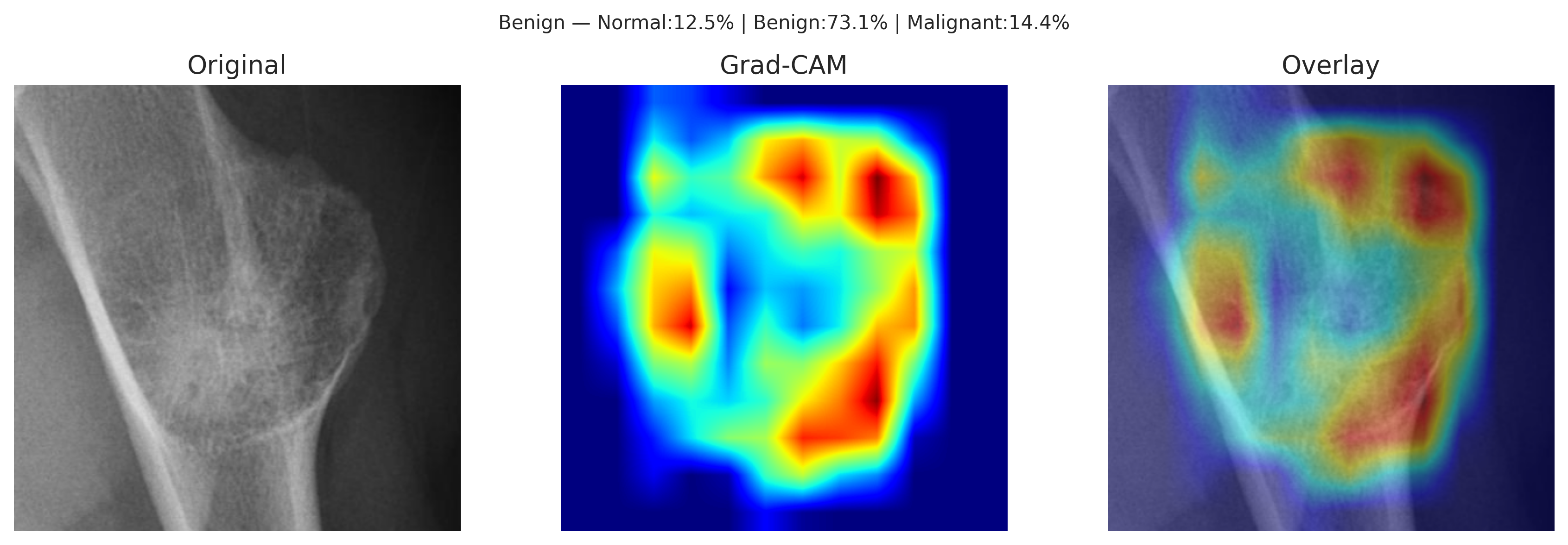

Supplement: Supplementary file 1 [file diagnostics-16-01811-s001.zip › Figure_S9_fold1_Benign_1.png]

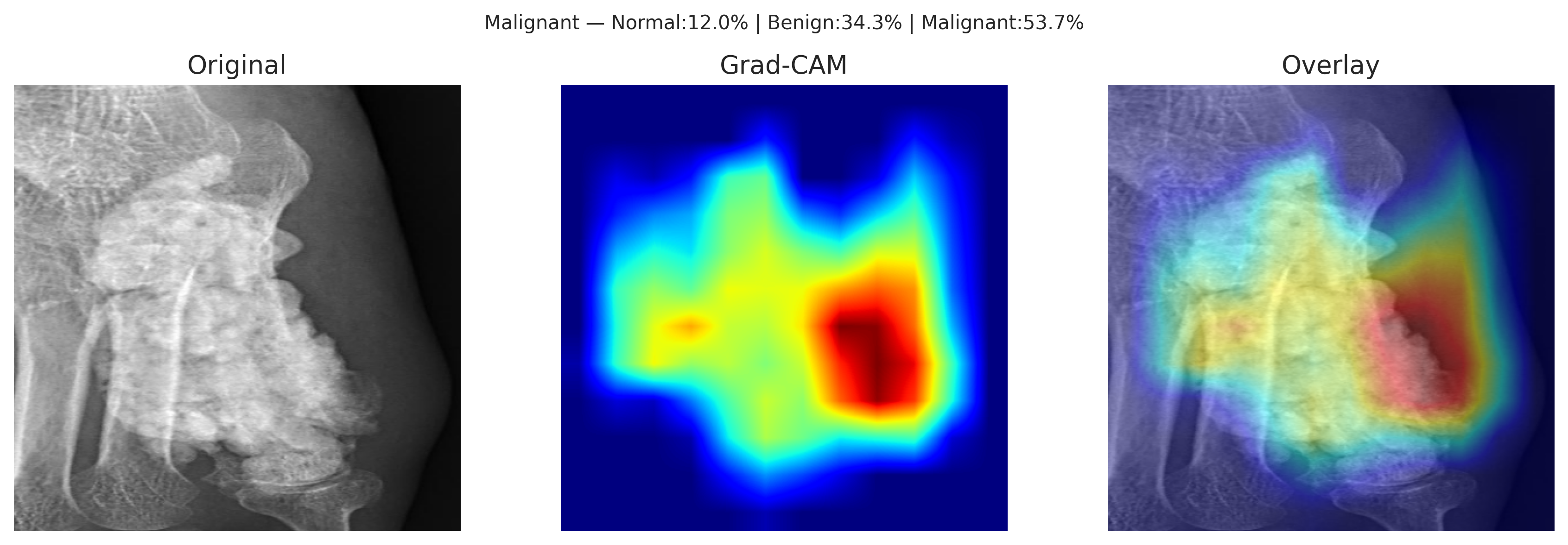

Supplement: Supplementary file 1 [file diagnostics-16-01811-s001.zip › Figure_S9_fold1_Malignant_0.png]

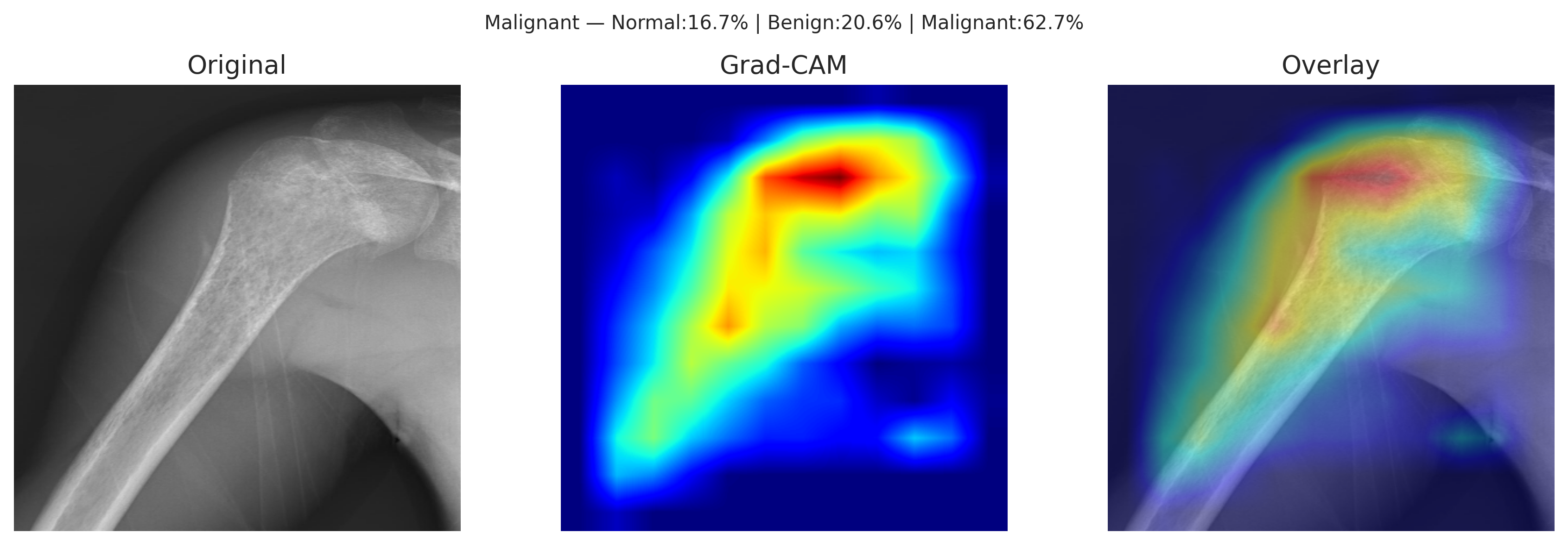

Supplement: Supplementary file 1 [file diagnostics-16-01811-s001.zip › Figure_S9_fold1_Malignant_1.png]

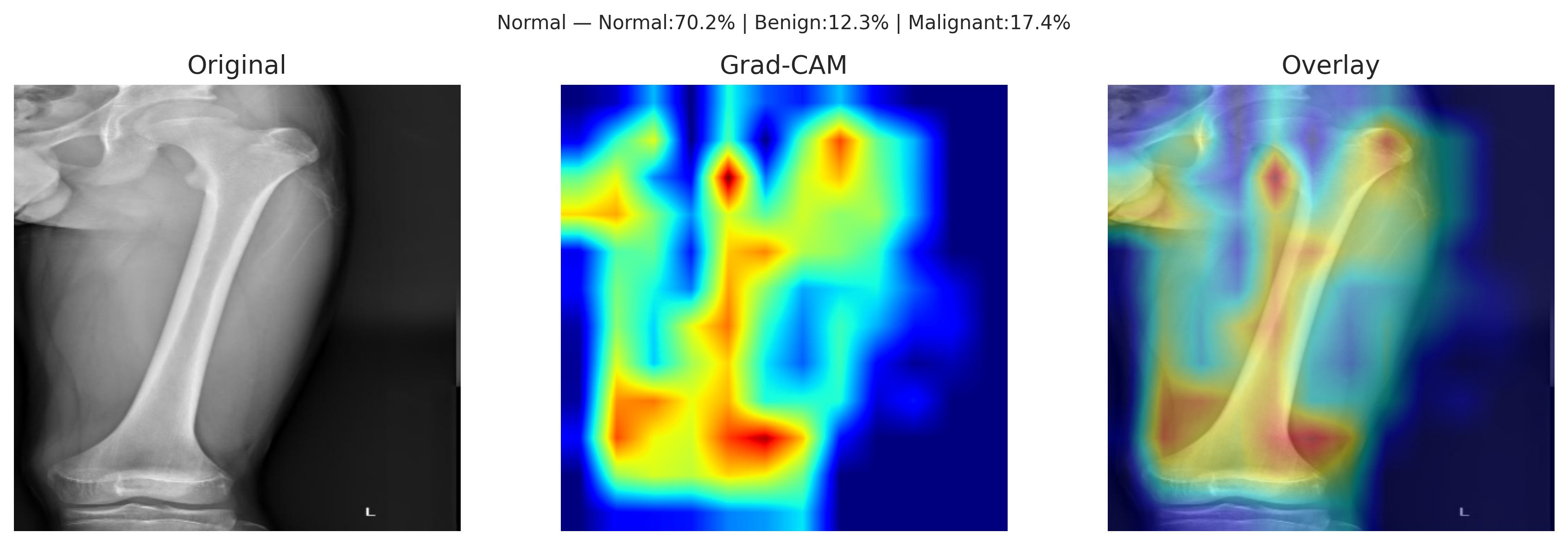

Supplement: Supplementary file 1 [file diagnostics-16-01811-s001.zip › Figure_S9_fold1_Normal_0.png]

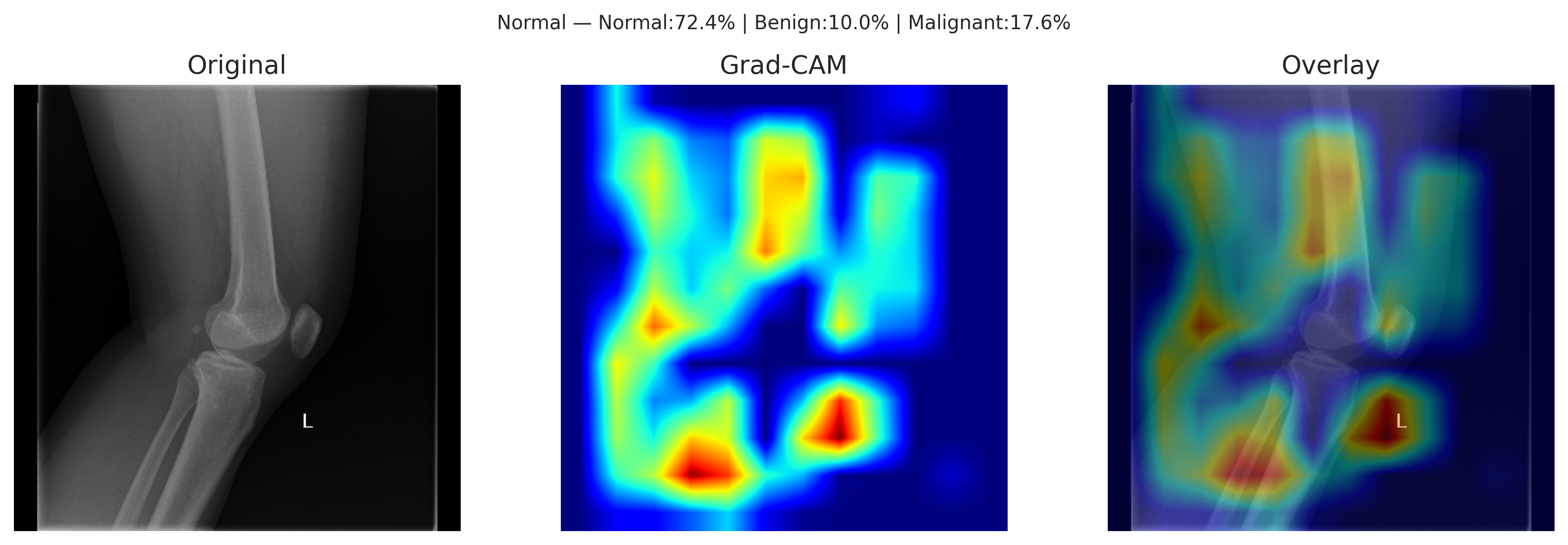

Supplement: Supplementary file 1 [file diagnostics-16-01811-s001.zip › Figure_S9_fold1_Normal_1.png]

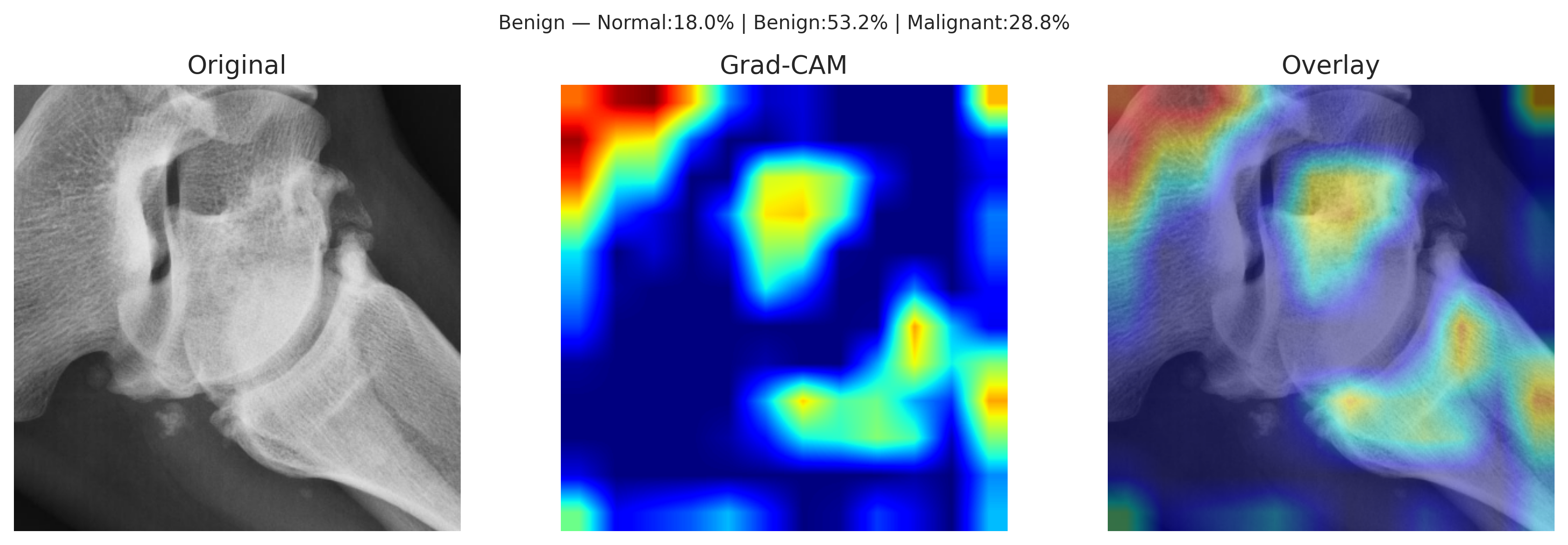

Supplement: Supplementary file 1 [file diagnostics-16-01811-s001.zip › Figure_S10_fold2_Benign_0.png]

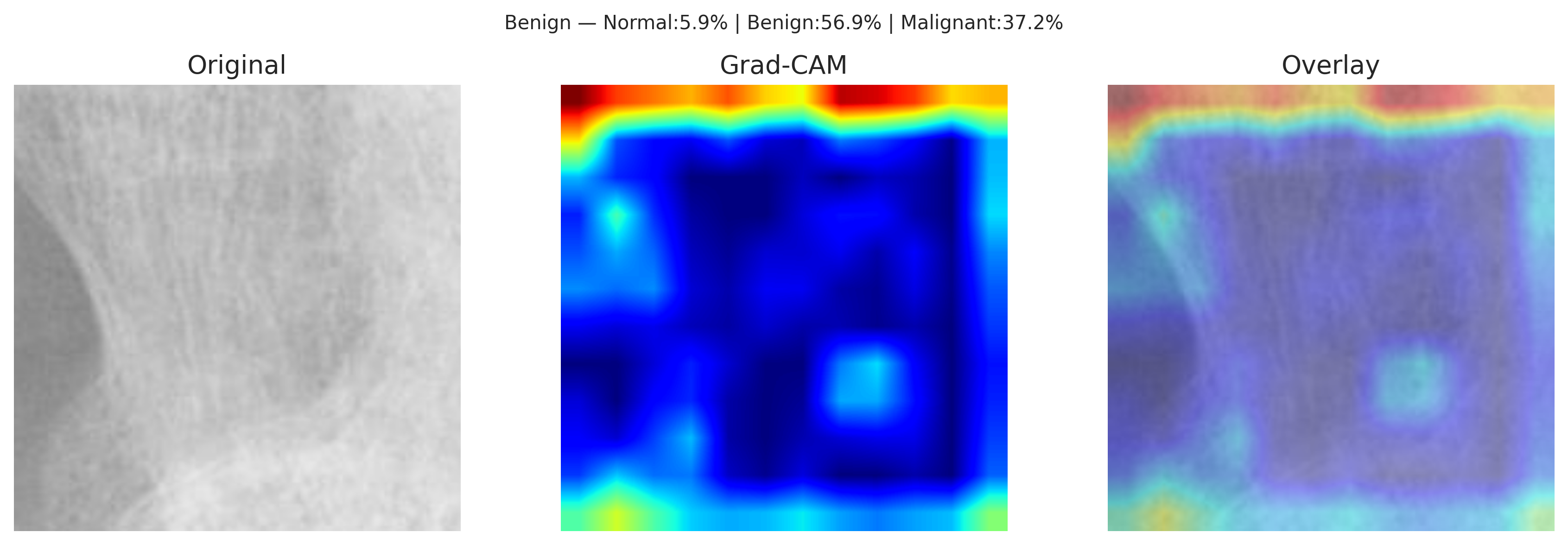

Supplement: Supplementary file 1 [file diagnostics-16-01811-s001.zip › Figure_S10_fold2_Benign_1.png]

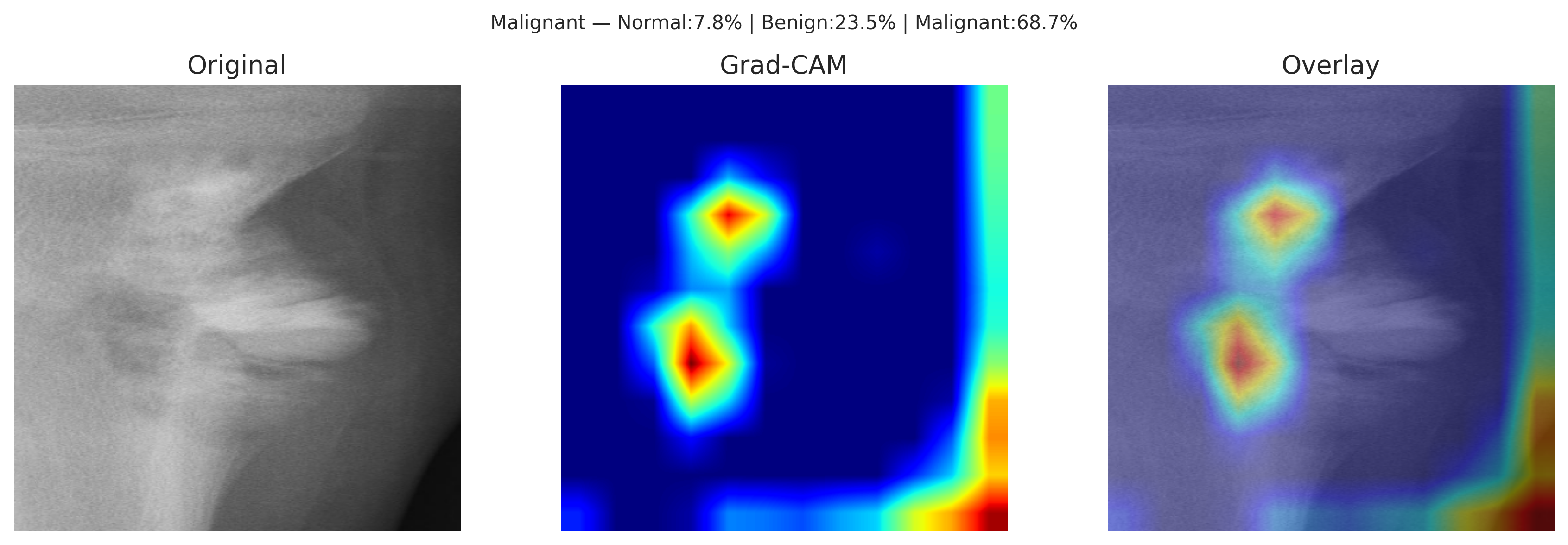

Supplement: Supplementary file 1 [file diagnostics-16-01811-s001.zip › Figure_S10_fold2_Malignant_0.png]

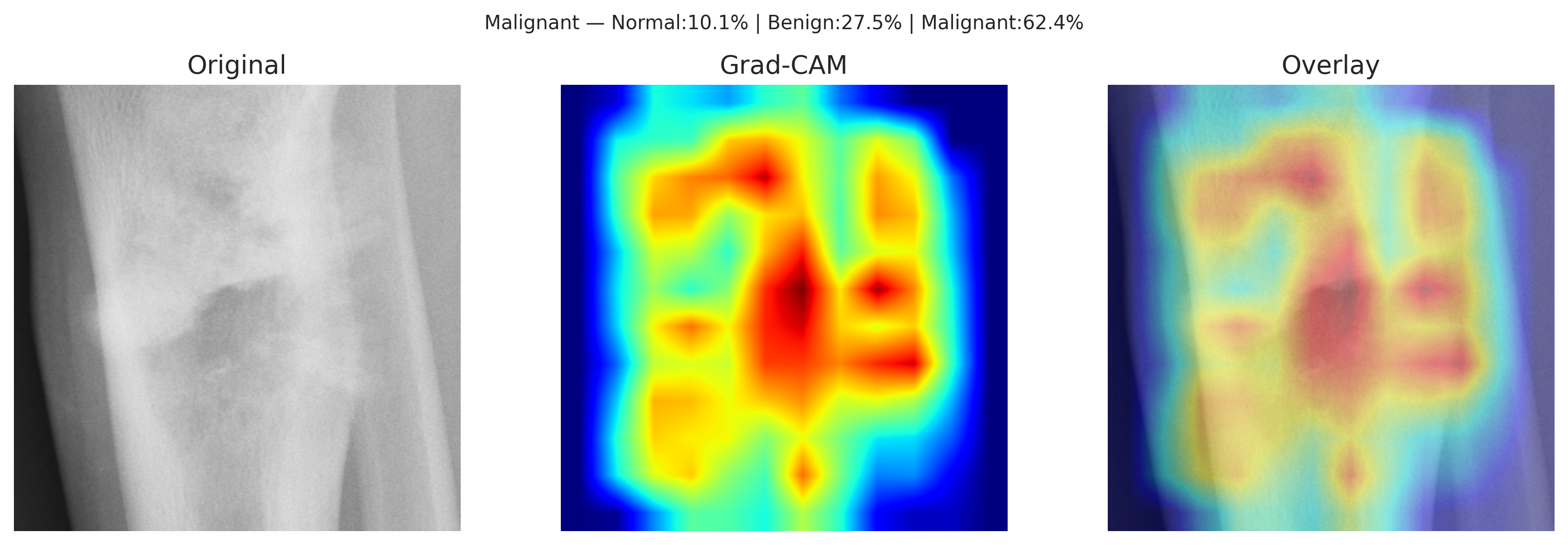

Supplement: Supplementary file 1 [file diagnostics-16-01811-s001.zip › Figure_S10_fold2_Malignant_1.png]

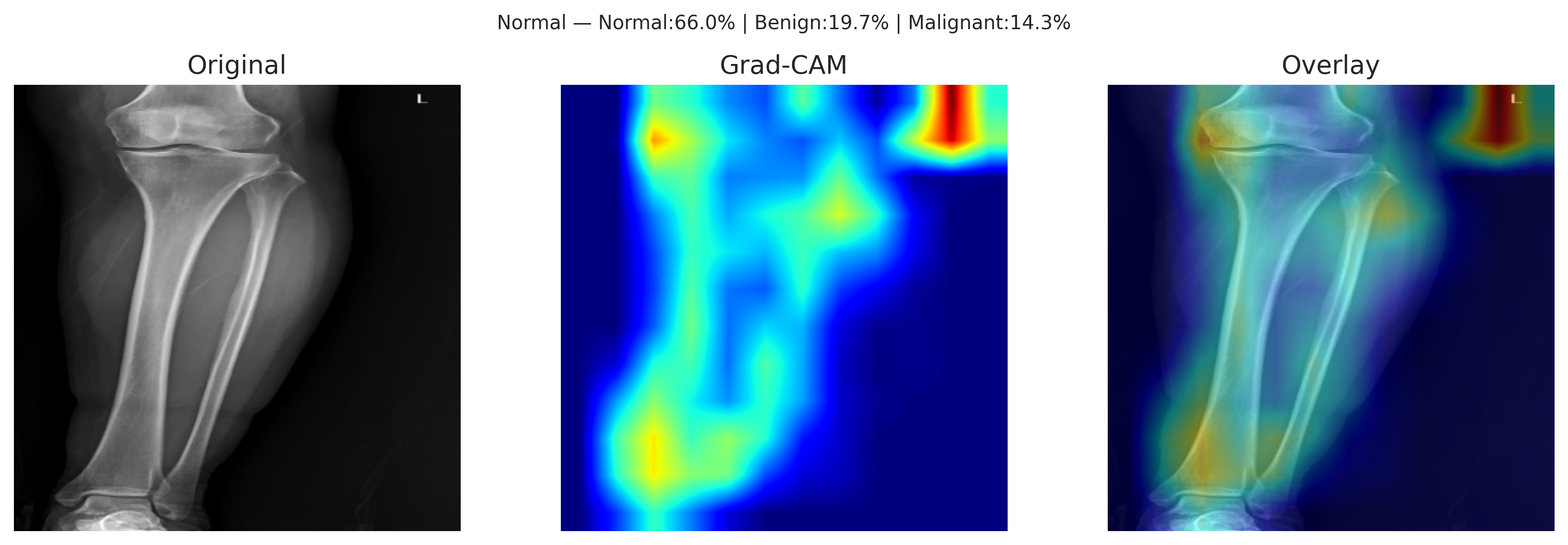

Supplement: Supplementary file 1 [file diagnostics-16-01811-s001.zip › Figure_S10_fold2_Normal_0.png]

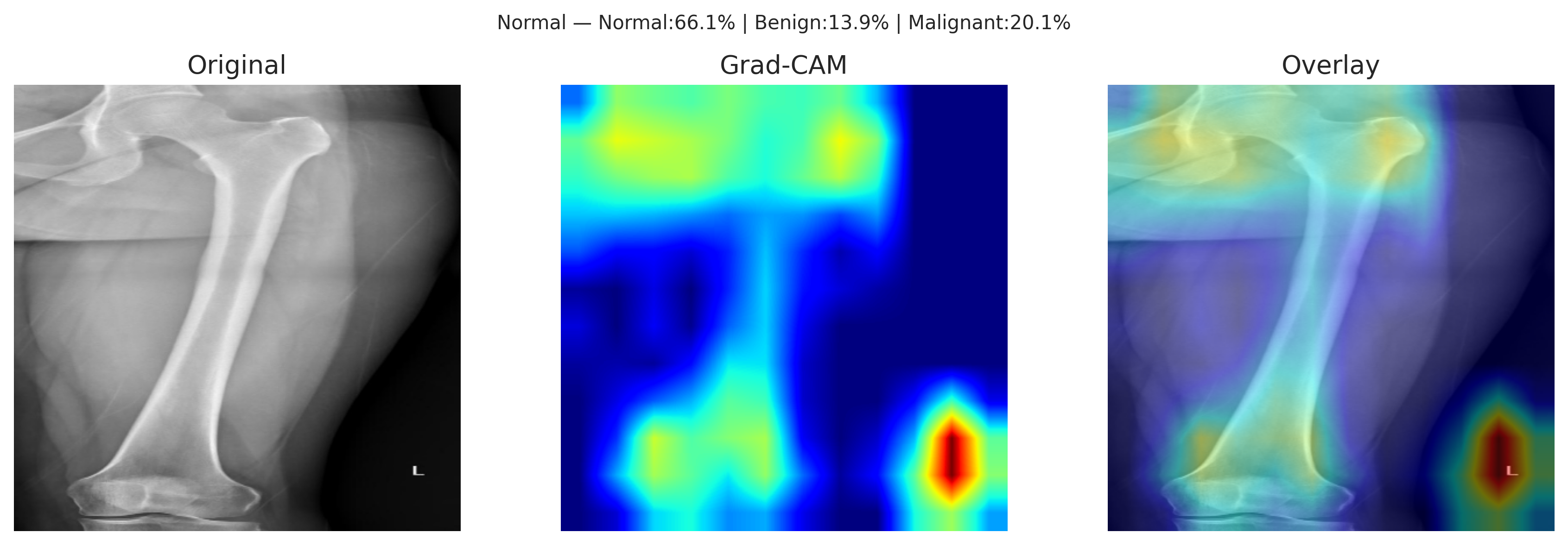

Supplement: Supplementary file 1 [file diagnostics-16-01811-s001.zip › Figure_S10_fold2_Normal_1.png]

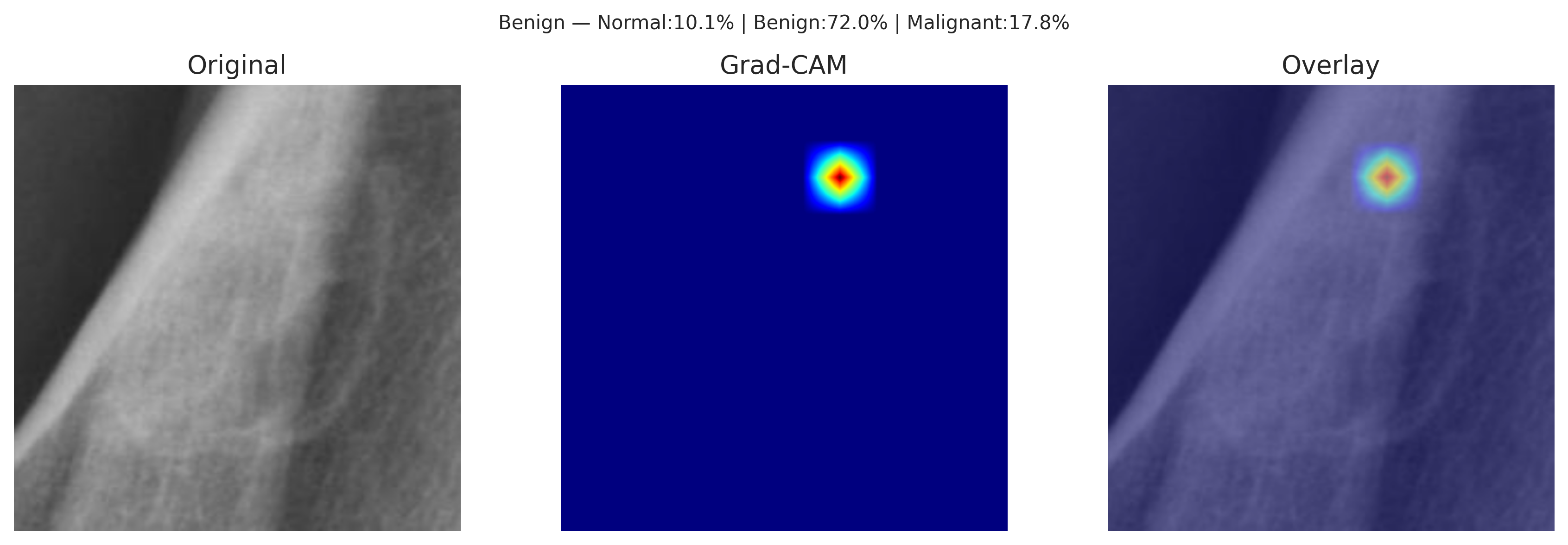

Supplement: Supplementary file 1 [file diagnostics-16-01811-s001.zip › Figure_S11_fold3_Benign_0.png]

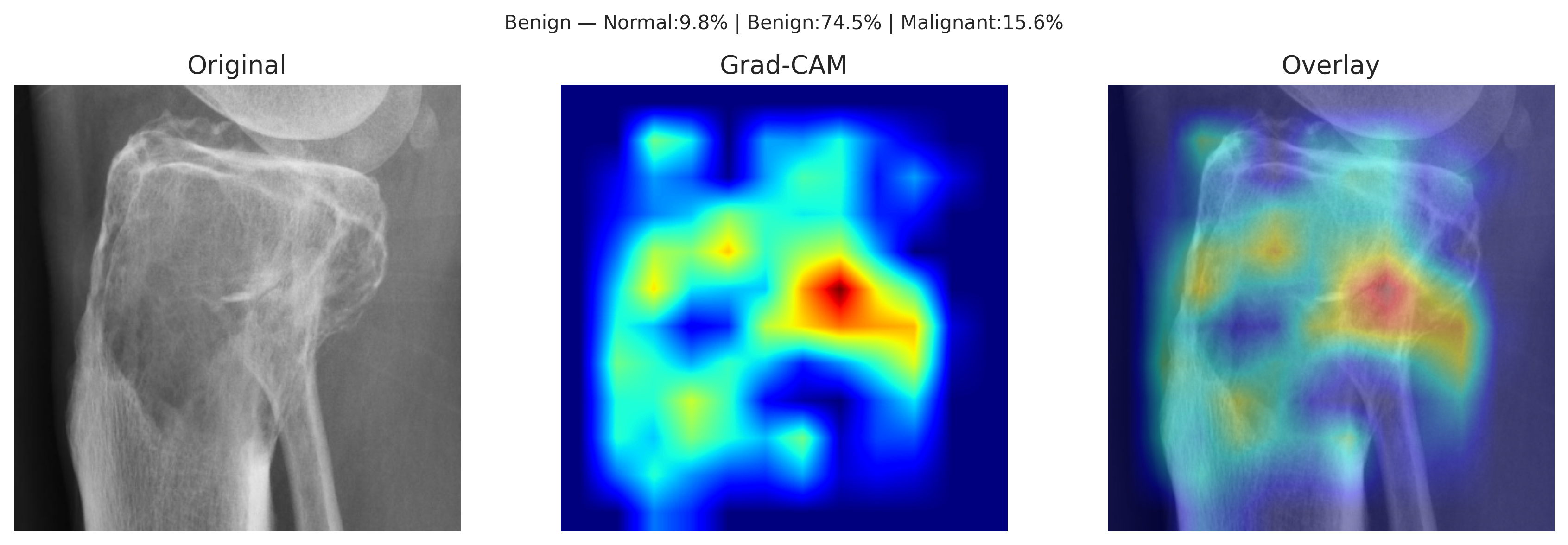

Supplement: Supplementary file 1 [file diagnostics-16-01811-s001.zip › Figure_S11_fold3_Benign_1.png]

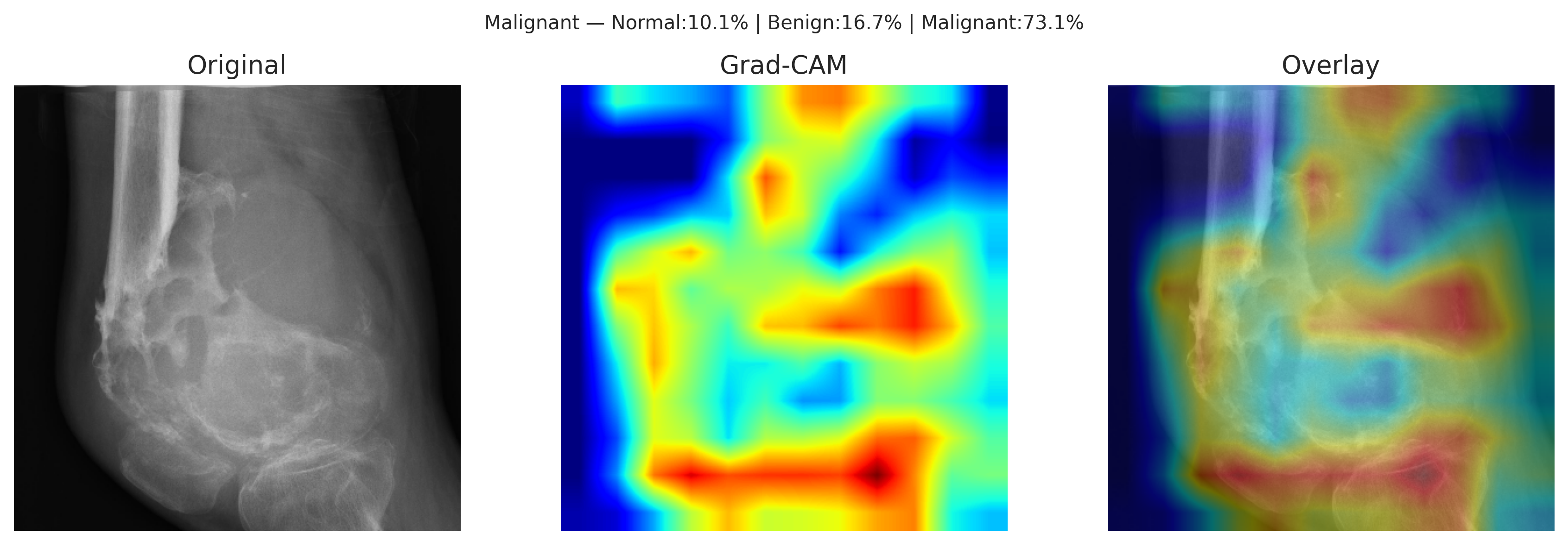

Supplement: Supplementary file 1 [file diagnostics-16-01811-s001.zip › Figure_S11_fold3_Malignant_0.png]

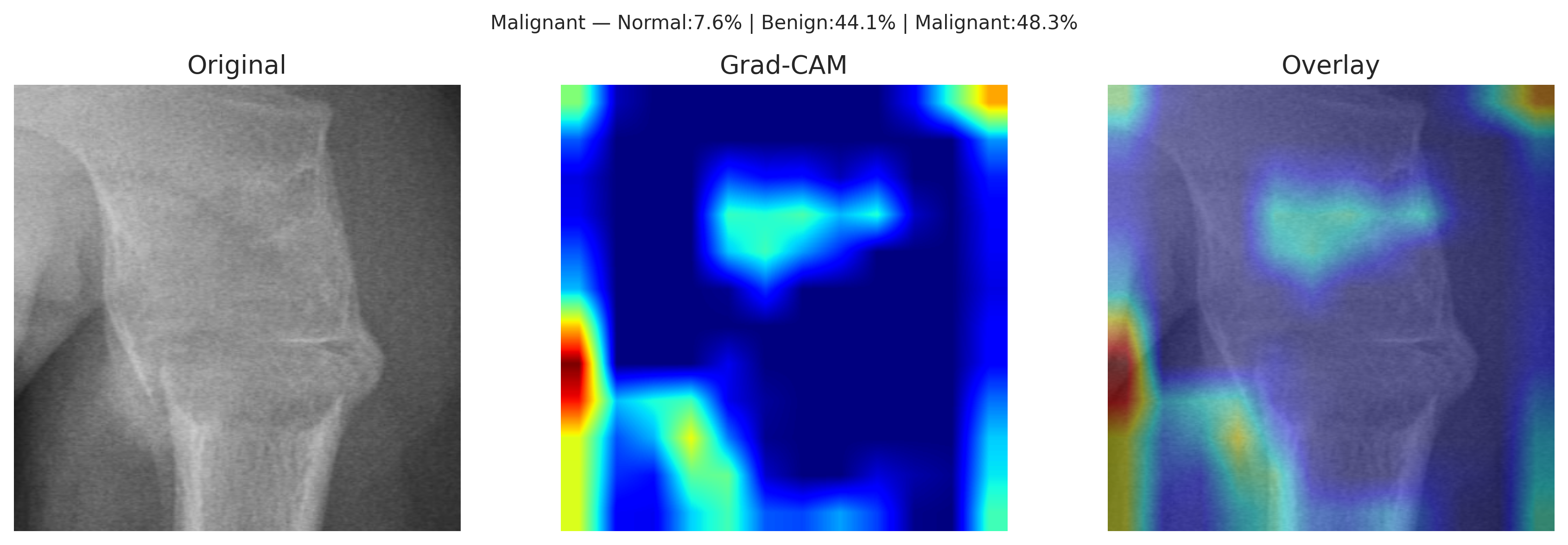

Supplement: Supplementary file 1 [file diagnostics-16-01811-s001.zip › Figure_S11_fold3_Malignant_1.png]

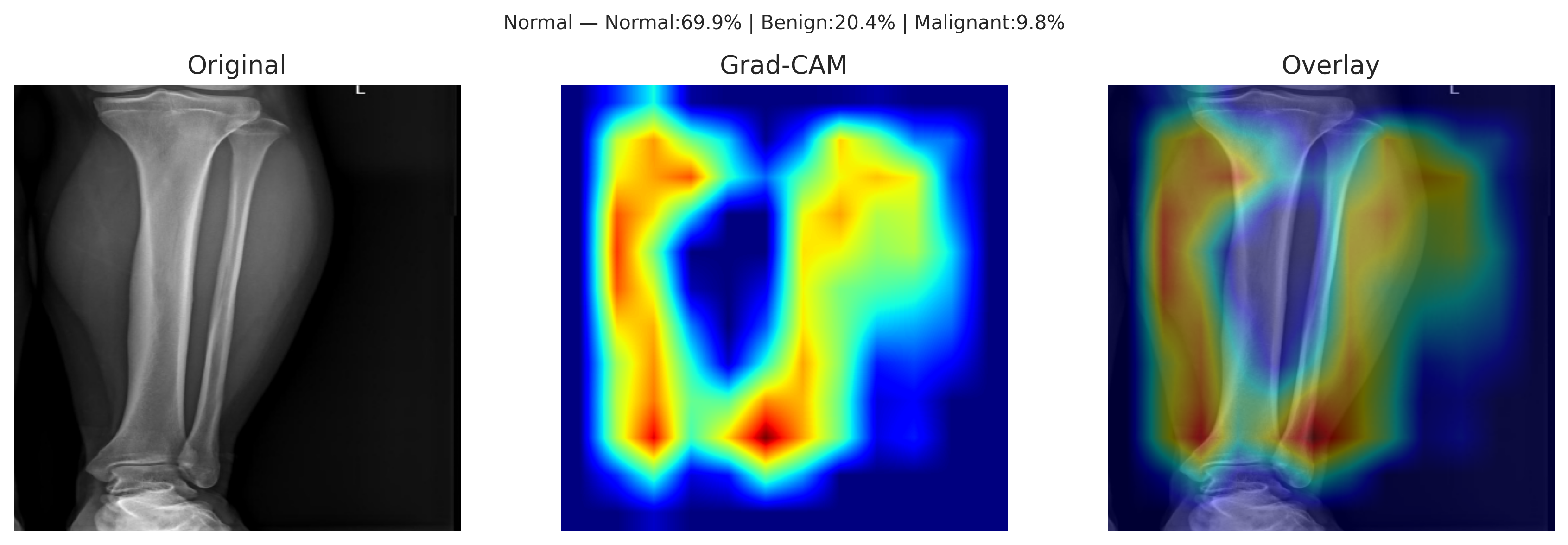

Supplement: Supplementary file 1 [file diagnostics-16-01811-s001.zip › Figure_S11_fold3_Normal_0.png]

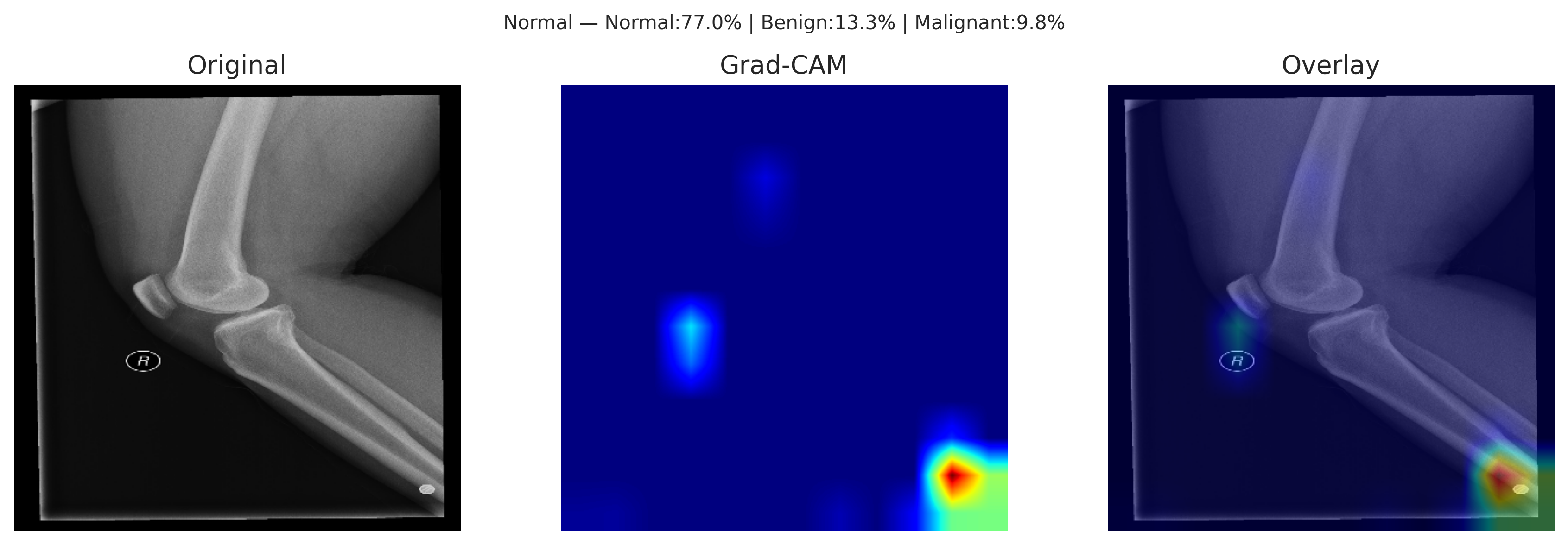

Supplement: Supplementary file 1 [file diagnostics-16-01811-s001.zip › Figure_S11_fold3_Normal_1.png]

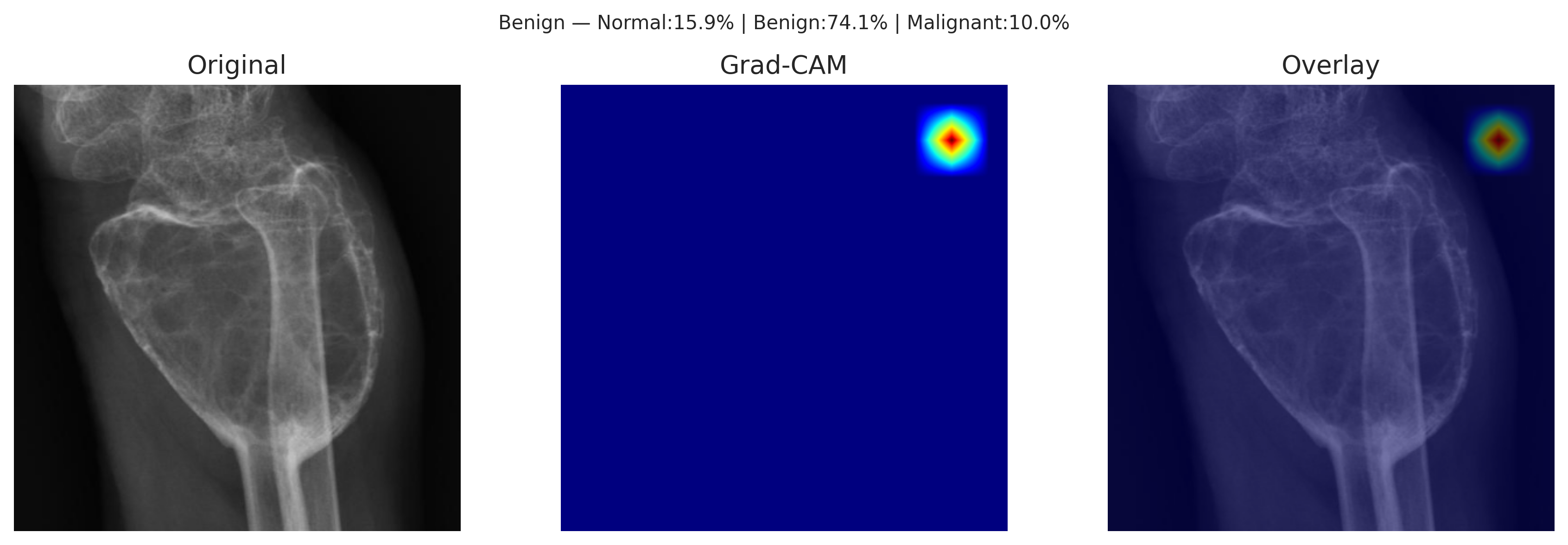

Supplement: Supplementary file 1 [file diagnostics-16-01811-s001.zip › Figure_S12_fold4_Benign_0.png]

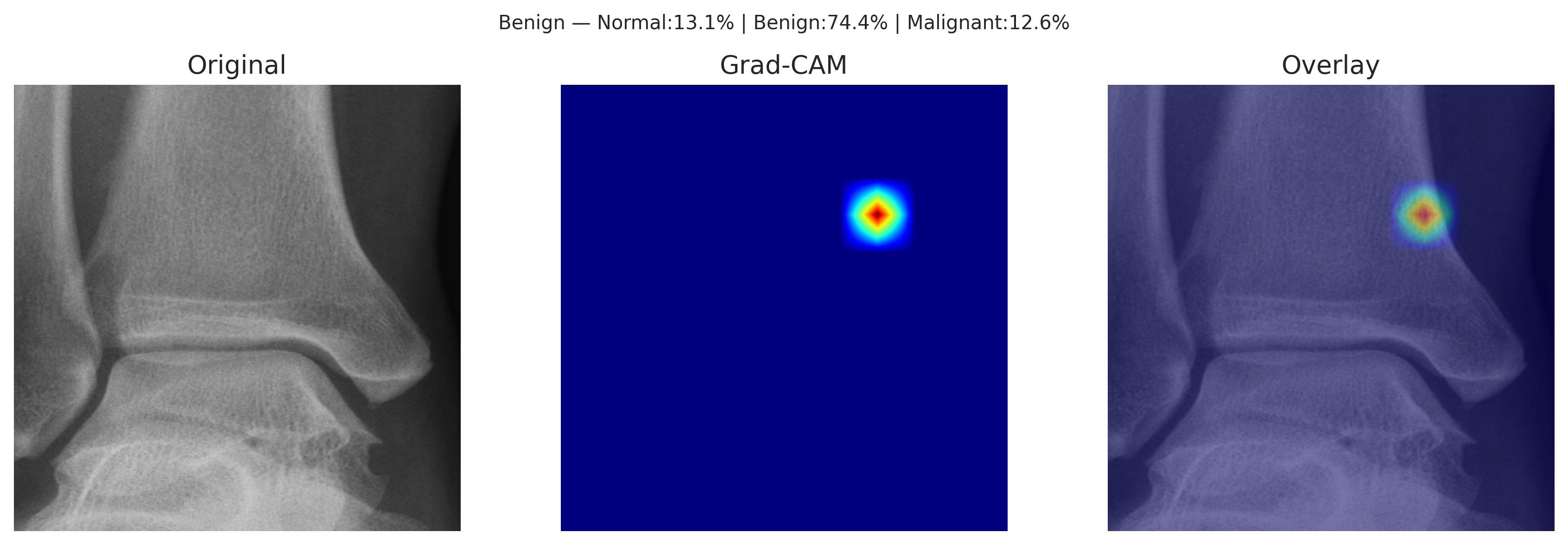

Supplement: Supplementary file 1 [file diagnostics-16-01811-s001.zip › Figure_S12_fold4_Benign_1.png]

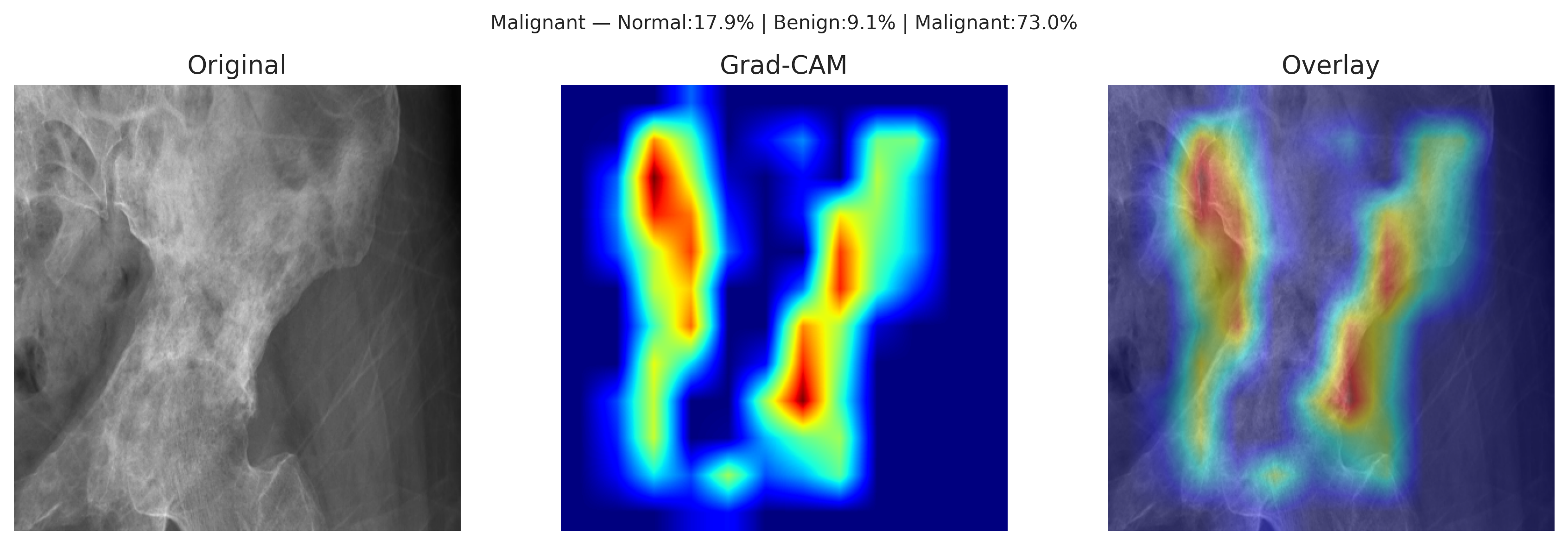

Supplement: Supplementary file 1 [file diagnostics-16-01811-s001.zip › Figure_S12_fold4_Malignant_0.png]

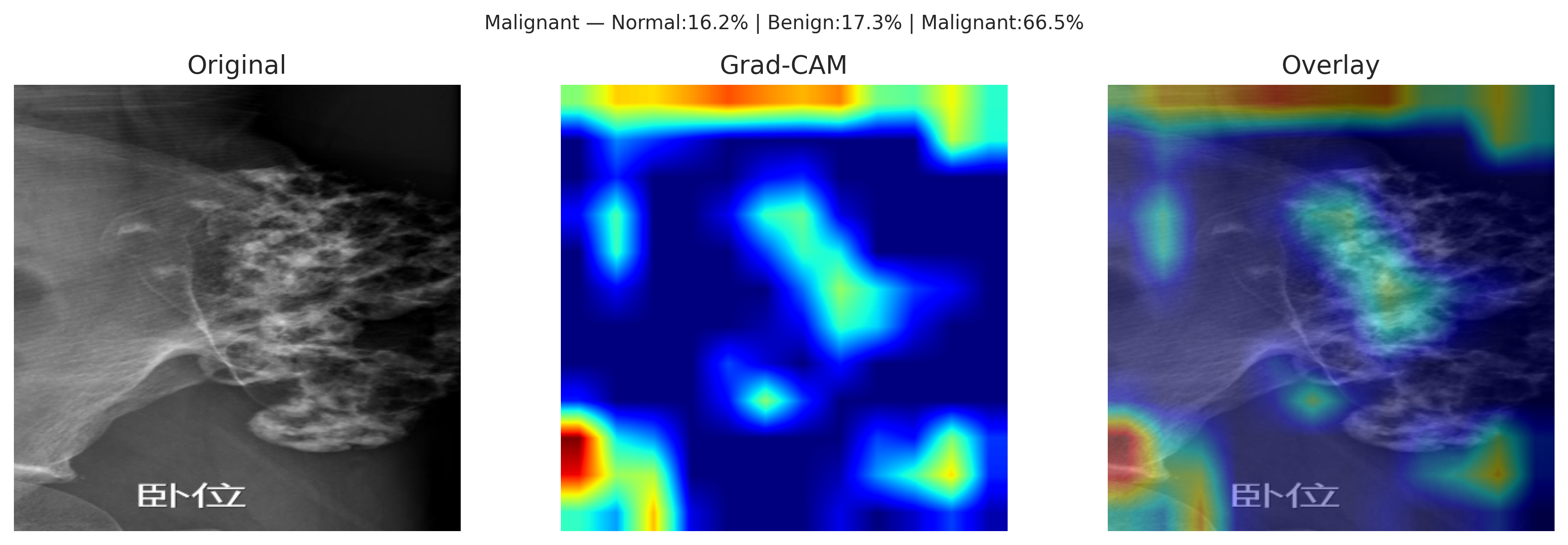

Supplement: Supplementary file 1 [file diagnostics-16-01811-s001.zip › Figure_S12_fold4_Malignant_1.png]

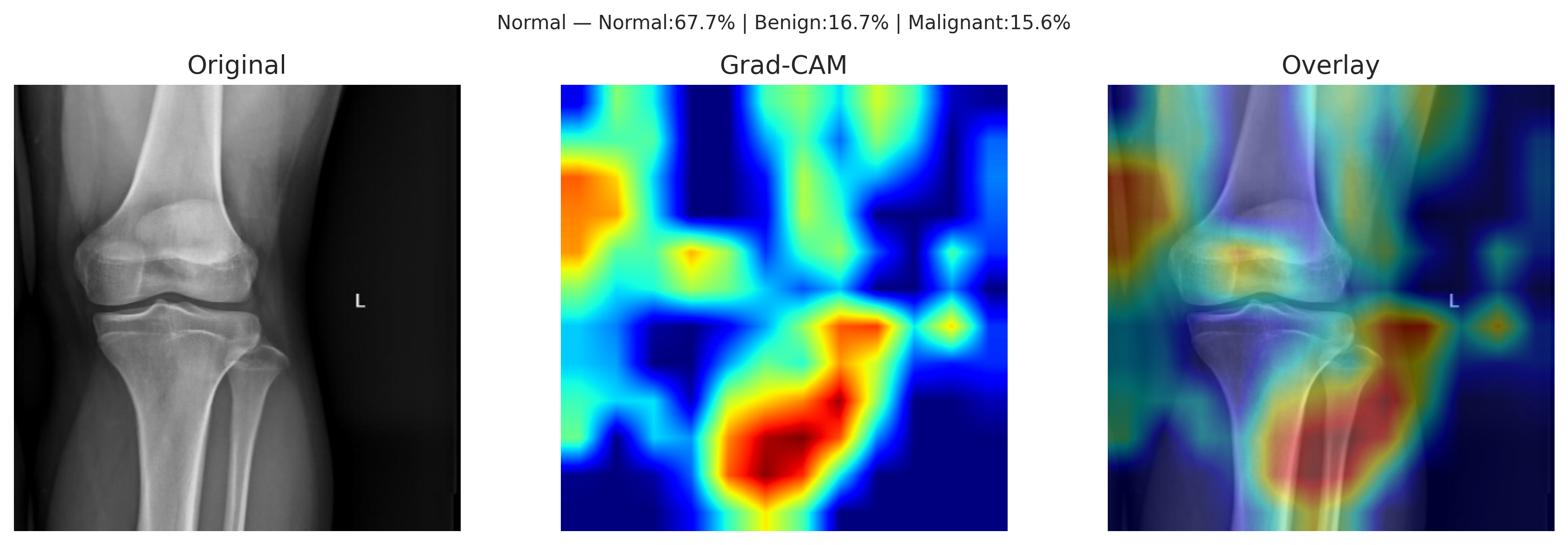

Supplement: Supplementary file 1 [file diagnostics-16-01811-s001.zip › Figure_S12_fold4_Normal_0.png]

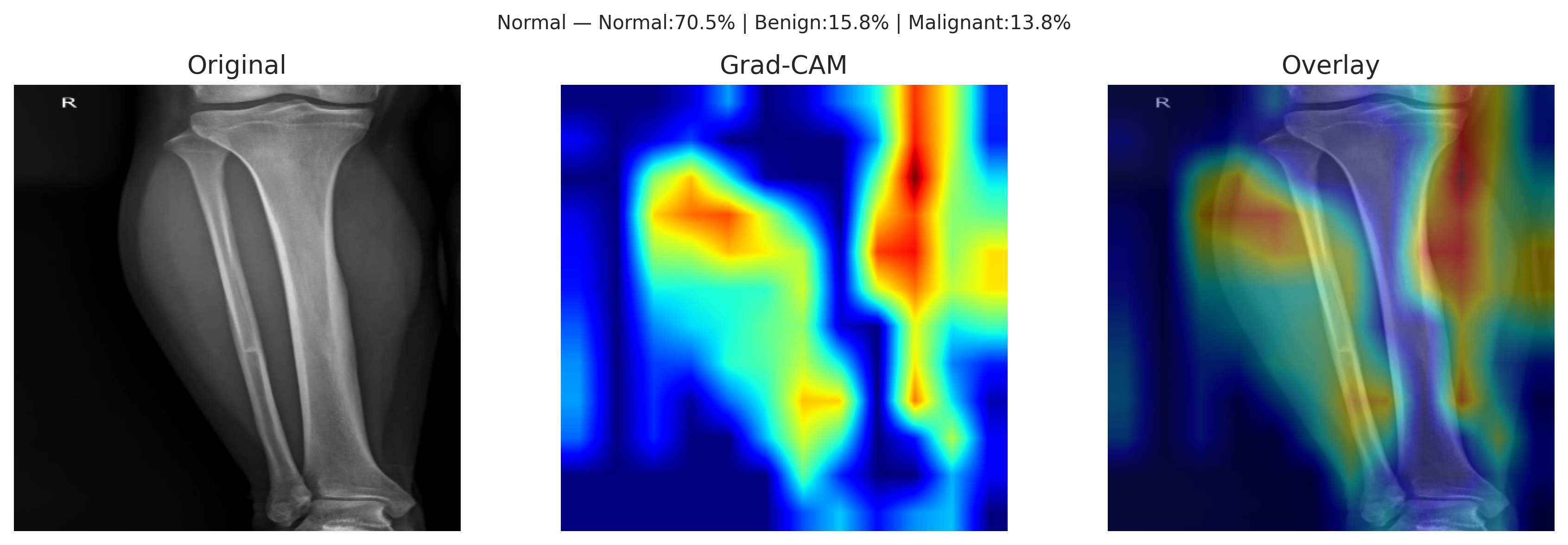

Supplement: Supplementary file 1 [file diagnostics-16-01811-s001.zip › Figure_S12_fold4_Normal_1.png]

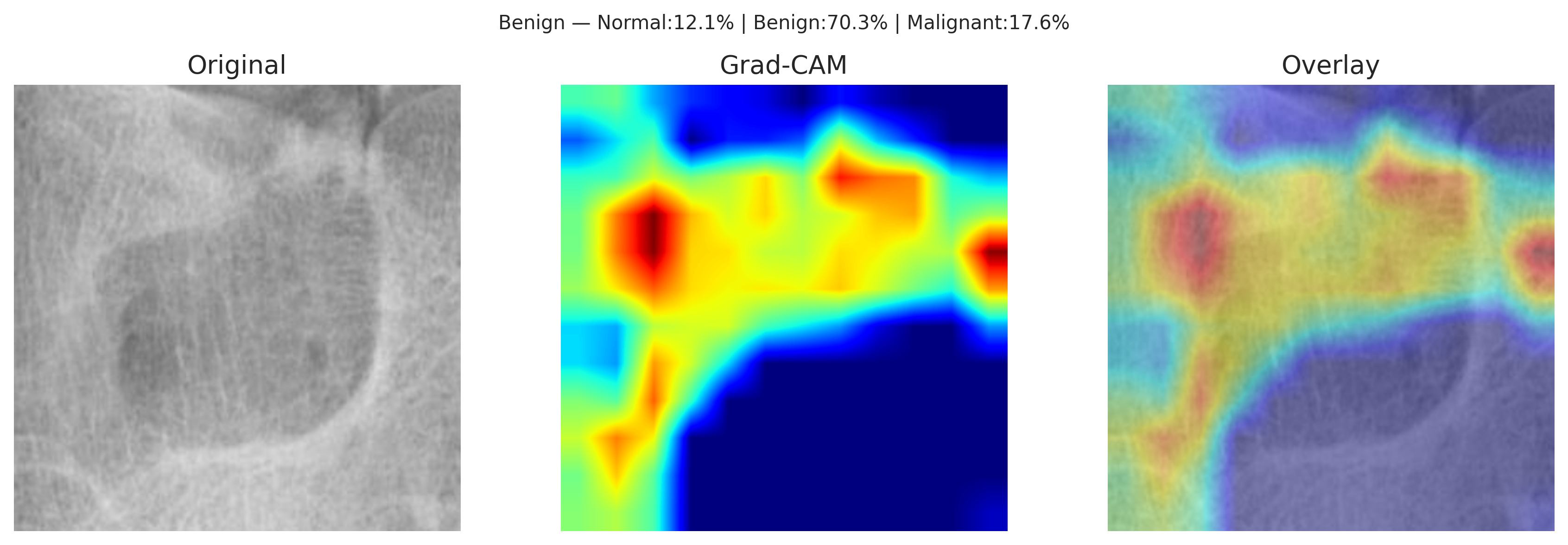

Supplement: Supplementary file 1 [file diagnostics-16-01811-s001.zip › Figure_S13_fold5_Benign_0.png]

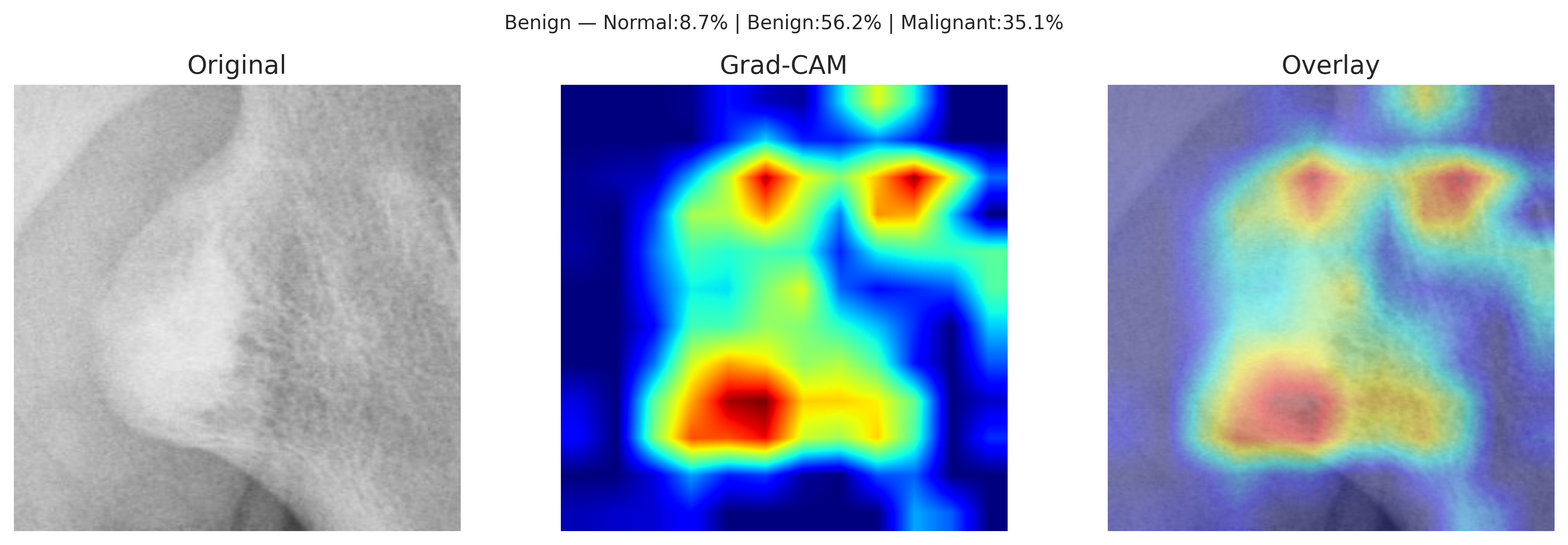

Supplement: Supplementary file 1 [file diagnostics-16-01811-s001.zip › Figure_S13_fold5_Benign_1.png]

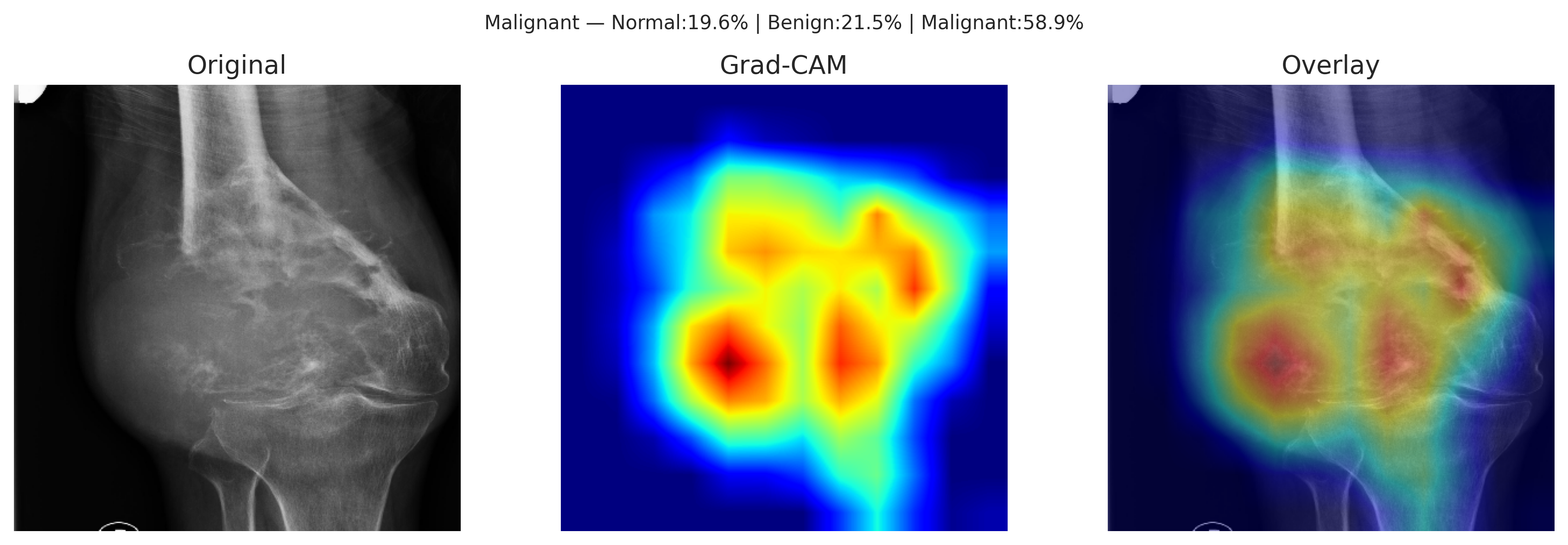

Supplement: Supplementary file 1 [file diagnostics-16-01811-s001.zip › Figure_S13_fold5_Malignant_0.png]

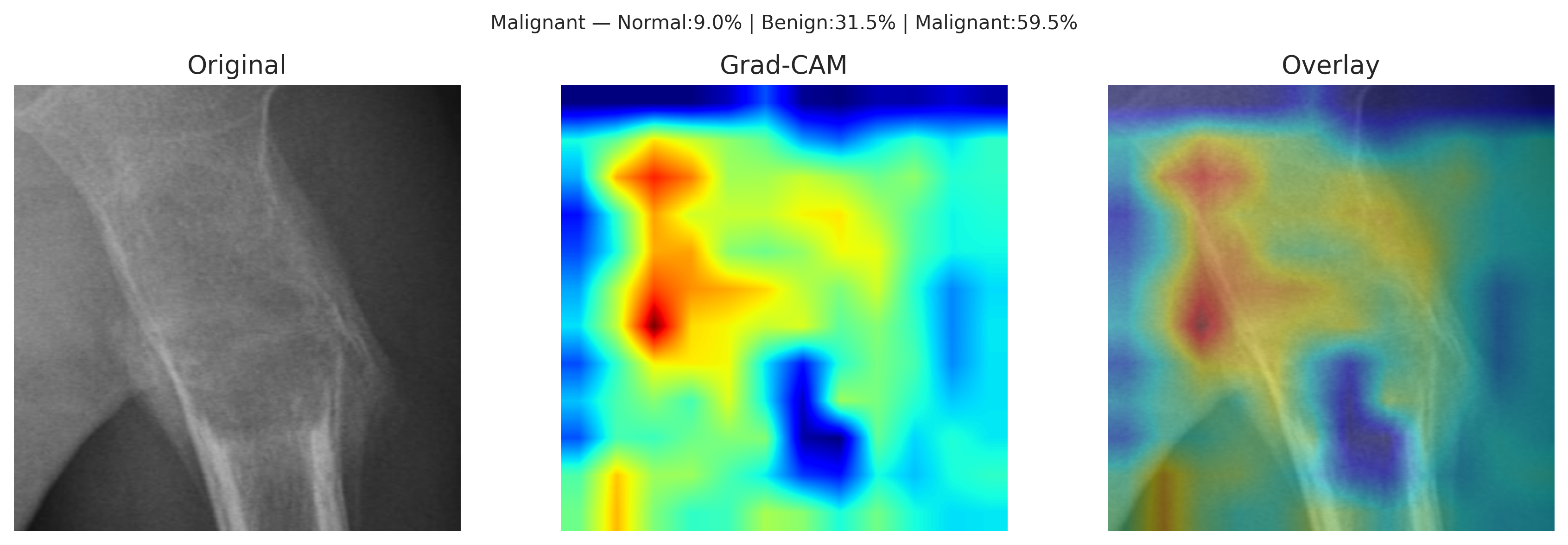

Supplement: Supplementary file 1 [file diagnostics-16-01811-s001.zip › Figure_S13_fold5_Malignant_1.png]

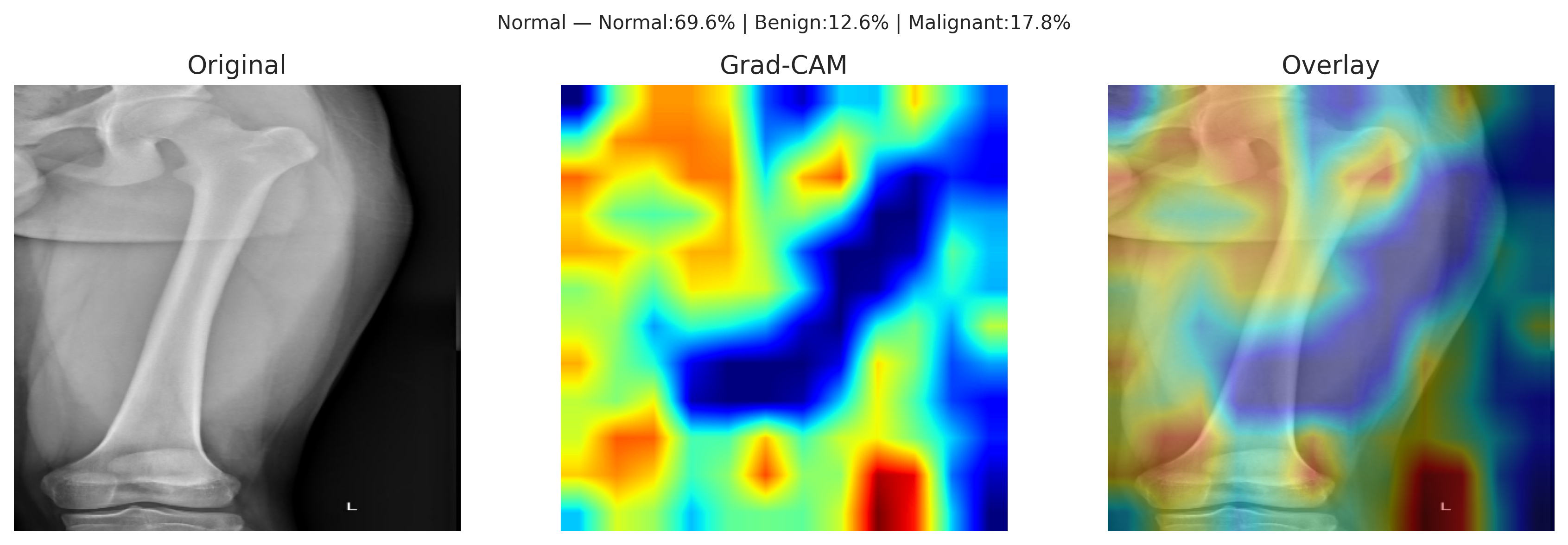

Supplement: Supplementary file 1 [file diagnostics-16-01811-s001.zip › Figure_S13_fold5_Normal_0.png]

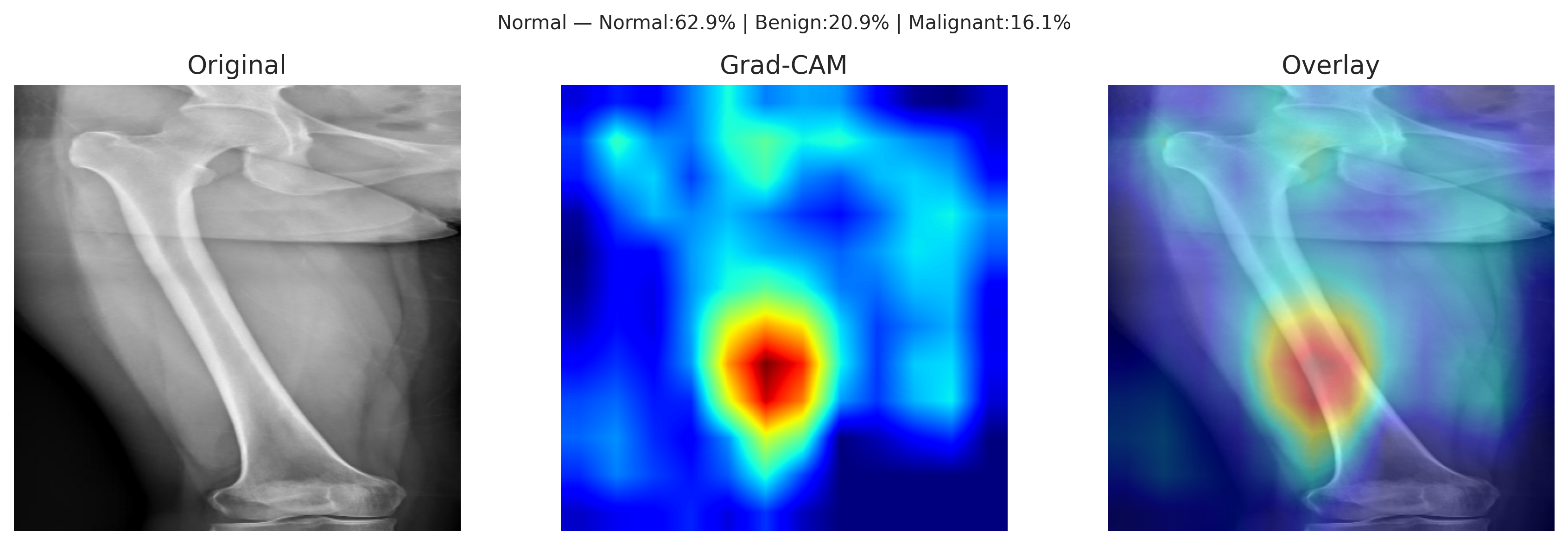

Supplement: Supplementary file 1 [file diagnostics-16-01811-s001.zip › Figure_S13_fold5_Normal_1.png]

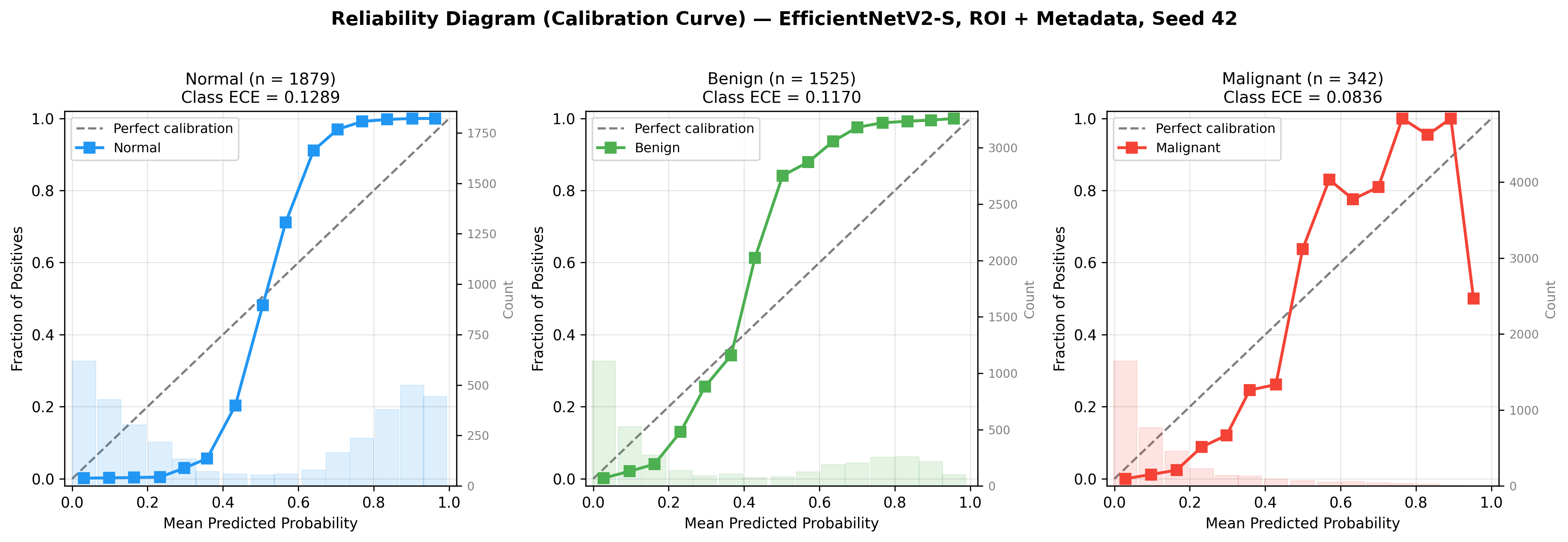

Supplement: Supplementary file 1 [file diagnostics-16-01811-s001.zip › Figure_S14_reliability_diagram.png]

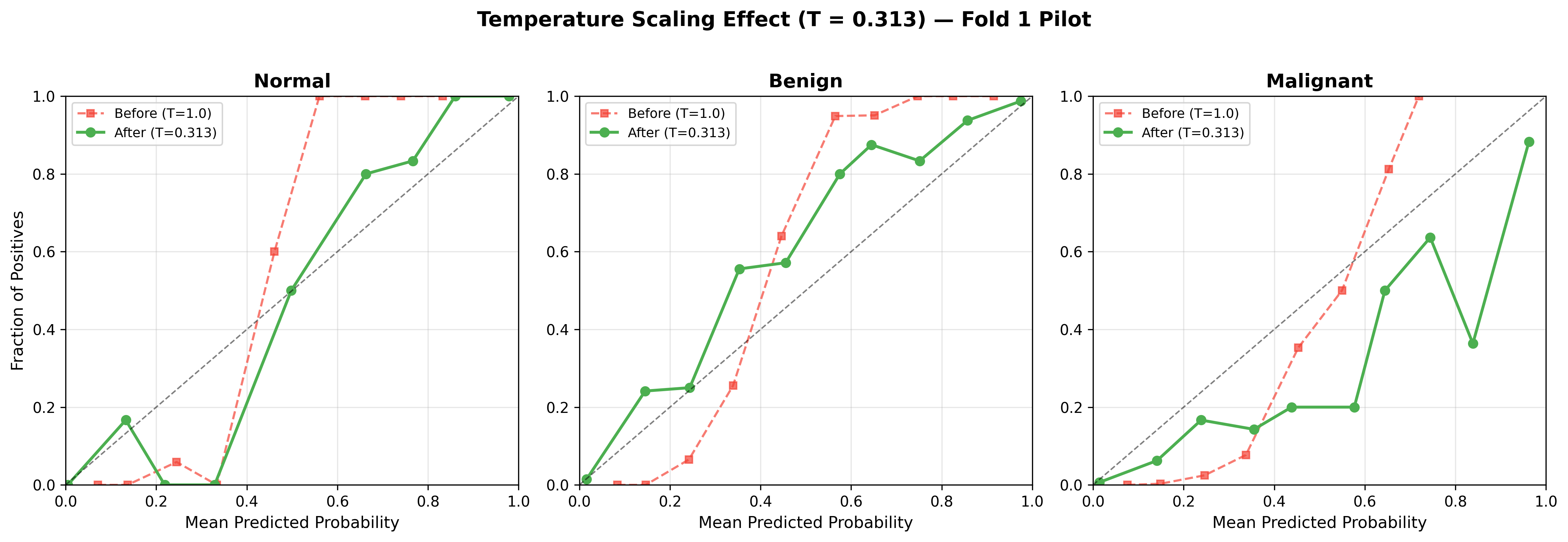

Supplement: Supplementary file 1 [file diagnostics-16-01811-s001.zip › Figure_S15_temperature_scaling_effect.png]

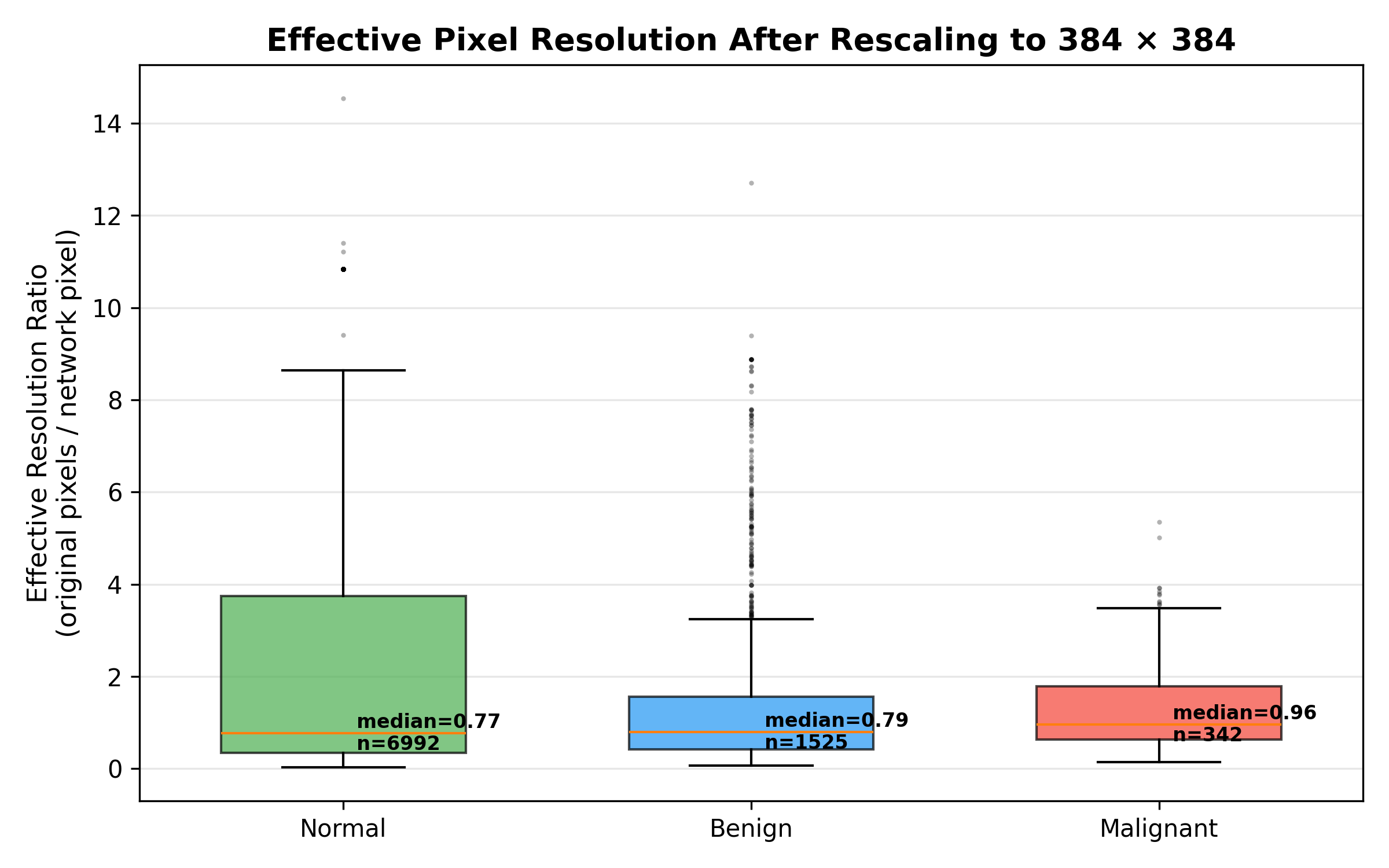

Supplement: Supplementary file 1 [file diagnostics-16-01811-s001.zip › Figure_S16_resolution_boxplot.png]

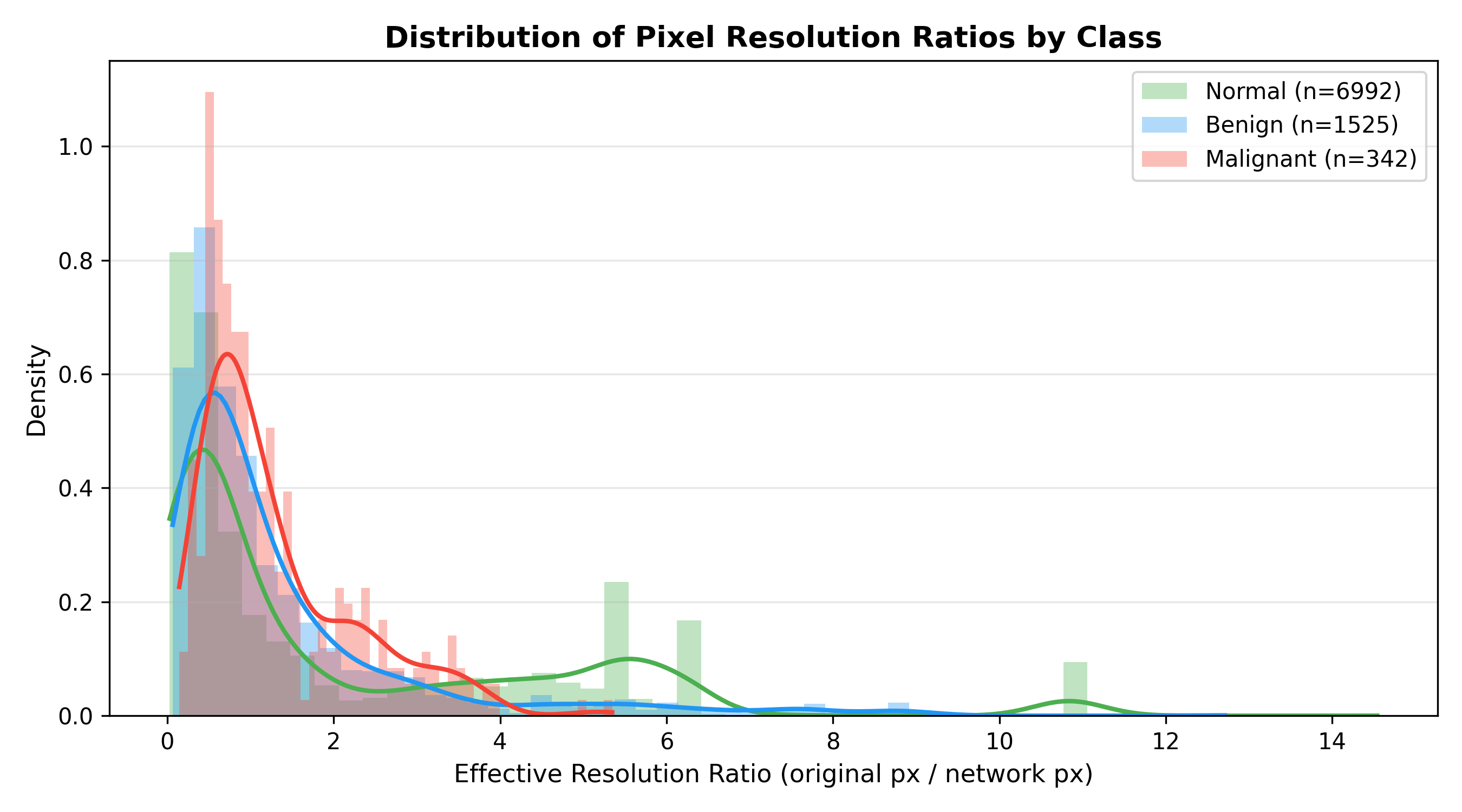

Supplement: Supplementary file 1 [file diagnostics-16-01811-s001.zip › Figure_S17_resolution_histogram.png]
